# Replication-independent eviction of the histone variant H2B.8 reveals chromatin reprogramming during seed imbibition

**DOI:** 10.1038/s41467-026-74230-6

**Published:** 2026-06-29

**Authors:** Lauriane Simon, Stefania Paltrinieri, Manon Verdier, Qingyi Wang, Sylviane Cotterell, David Latrasse, Aude Maugarny, Gilles Sireta, Sophie Desset, Simon Amiard, Christophe Bailly, Kentaro Tamura, Moussa Benhamed, Christophe Tatout, Samuel Le Goff, Aline V. Probst

**Affiliations:** 1 iGReD, Université Clermont Auvergne, CNRS, Inserm, BP 38, 63001 Clermont-Ferrand, France; 2 Université Paris-Saclay, CNRS, INRAE, Univ Evry, Institute of Plant Sciences Paris-Saclay (IPS2), 91405 Orsay, France; 3 Sorbonne Université, CNRS, Inserm, Development, Adaptation and Ageing, Dev2A, F-75005 Paris, France; 4 Sorbonne Université, CNRS, Inserm, Institut de Biologie Paris-Seine, IBPS, F-75005 Paris, France; 5 Department of Environmental and Life Sciences, University of Shizuoka, Shizuoka, 422-8526 Japan; 6 Institut Universitaire de France (IUF), Orsay, France

**Keywords:** Plant molecular biology, Epigenetics, Seed development

## Abstract

The transition from seed to seedling involves major changes in nuclear organization and gene expression, yet the extent to which this developmental transition requires chromatin reprogramming remains largely unexplored. Here, we report that Arabidopsis dry seed embryos accumulate the histone variant H2B.8, which contributes to higher-order chromatin organization by forming spatial clusters that structure the 3D nuclear space. H2B.8 predominantly assembles into heterotypic nucleosomes, is enriched at euchromatic transposons and lowly expressed genes and, during imbibition, modulates the transcriptional activation of a subset of these genes. Water uptake triggers a decrease in *H2B.8* transcripts and the eviction of the H2B.8 variant from chromatin, in a process that operates independently of DNA replication but requires de novo transcription. Histone eviction is not restricted to H2B.8, as imbibition also induces turnover of the H3.3 histone variant and therefore initiates a replication-independent chromatin reprogramming process. These findings highlight a fundamental mechanism of epigenetic regulation during early plant development.

## Abstract (web summary)

The transition from seed to seedling requires chromatin reprogramming. By studying the histone variant H2B.8, the authors show that it organizes silent chromatin in dry seeds and reveal that water uptake triggers replication-independent histone turnover.

## Introduction

Seeds were a major innovation of photosynthetic organisms, contributing to the reproductive success of angiosperms. As seeds mature, they accumulate nutrients and reserves and acquire tolerance to desiccation, enabling long-term survival at low moisture levels. Despite their largely quiescent state, during which little transcriptional activity can be observed^1,2^, seeds remain able to sense the environment and time germination to coincide with favorable environmental conditions, compatible with the completion of the plant’s life cycle, such as temperature and soil moisture content. Reserve proteins and mRNA molecules accumulate during seed maturation and serve as templates for de novo protein synthesis^3^. Protein biosynthesis from these stored mRNA resumes upon water uptake during seed imbibition, as well as various metabolic and physiological activities, such as the mobilization of reserves to restart respiration and energy production, and the initiation of repair mechanisms^4,5^. Imbibition also triggers changes in nuclear organization, as the volume of the small nuclei with highly condensed chromatin in the dry seeds of *Arabidopsis thaliana* progressively increases during water uptake and germination^6^. The transition from seed to seedling further requires the transcription of new mRNA, and more than 20,000 genes show differential expression^7–9^, revealing important transcriptome reprogramming during this developmental transition.

Gene expression as a readout of the eukaryotic genome is strongly influenced by its chromatin organization. However, the contribution of local or higher-order chromatin organization to the observed transcriptome reprogramming during this developmental transition is only partly understood. At the level of the nucleosome, the basic building block of chromatin, post-translational modifications (PTMs) of histone proteins signal repressive or permissive chromatin states and may directly influence nucleosome stability. The incorporation of specific histone variants further modifies nucleosome stability or DNA accessibility and can influence the setting of histone PTMs^10–12^. The Arabidopsis genome codes for multiple variants of the core histones H3, H2A, and H2B and the linker histone H1. This permits important combinatorial complexity, defining nucleosomes with different characteristics^13,14^. The deposition, maintenance, and exchange of histone variants are coordinated by a network of histone chaperones, which may exhibit specificity for a histone type or a particular variant. Histone chaperones, such as FACILITATES CHROMATIN TRANSCRIPTION (FACT), ensure the recycling of existing parental histones at the replication fork or during transcription^15,16^, thereby permitting the retention of existing histone PTMs. Others, such as CHROMATIN ASSEMBLY FACTOR 1 (CAF-1) and HISTONE REGULATOR A (HIRA), have been shown to coordinate de novo nucleosome assembly, thereby contributing to the reestablishment of specific histone modifications on the newly assembled nucleosome chains^12^ or the maintenance of nucleosomal occupancy genome wide, respectively^17–19^. The genomic distribution of histone variants has been extensively investigated in seedlings, revealing correlations with specific chromatin states. For instance, the replacement histone variant H3.3 is enriched at the 3’ end of transcriptionally active genes in seedling tissue, while H2A.W marks transposable elements^20–23^. During development, certain cell types express a particular histone variant repertoire, such as the small, highly condensed sperm nuclei of pollen, which are enriched in H3.10 and the angiosperm-specific H2B variant H2B.8^24,25^. While neither histone variant is essential for male fertility, closer investigation has shown that they fulfill specific functions. Due to amino acid variations around the lysine 27 site, H3.10 is a poor substrate for histone methyltransferases, and its incorporation in sperm chromatin contributes to reprogramming of the repressive H3K27me3 mark in paternal chromatin^24^. H2B.8, in turn, is involved in nuclear and chromatin condensation of sperm nuclei, mainly through its phase separation capacity mediated by its long and intrinsically disordered N-terminal tail, though it does not influence the sperm cell transcriptome^25^. Besides sperm nuclei, the H2B.8 variant is expressed during seed maturation^23,26^ during which chromatin condenses, and nuclear size progressively decreases^6^. Throughout this developmental time window, as DNA replication has ceased and *H3.1* expression is low, H2B.8 accumulates concomitantly with the replacement variant H3.3^27,28^. In dry seeds, H3.3 is also enriched at the 5’ ends of developmentally regulated genes and is required for their full activation during germination^27^. Consequently, H3.3 depletion in *H3.3* knockouts or reduced H3.3 incorporation in *HIRA* mutants leads to defects in germination and seed vigor^27,28^. These findings led to the hypothesis that the seed-specific chromatin organization plays a role in protecting the genome and maintaining a quiescent state. This further suggests that changes in local and higher-order chromatin organization are required to achieve full transcriptome reprogramming during germination and seedling establishment.

Here, we have investigated the organization of dry seed chromatin and determined the genomic distribution of the H2B histone variant H2B.8. We find that H2B.8 can form heterotypic nucleosomes containing one H2B.8 and one other H2B variant at genes and transposable elements on chromosome arms but is depleted from pericentromeric heterochromatin. The H2B.8-marked genes are globally repressed in dry seeds and co-enriched with gene-body H2A.Z and H3K27me3. While loss of H2B.8 does not affect the stored mRNA in dry seeds, the expression of a subset of H2B.8-marked genes is reduced in the absence of H2B.8 during imbibition. 3D microscopy coupled with Hi-C analysis further reveals that H2B.8 contributes to genome organization in the dry seed embryos, with H2B.8-enriched chromatin regions clustering in a H2B.8-dependent manner. Imbibition triggers reduction in stored *H2B.8* transcript levels, and the eviction of this histone H2B variant in a process that is independent of DNA replication but requires de novo transcription. We find that histone eviction is not restricted to H2B.8, revealing global replication-independent chromatin reprogramming during the early stages of seed germination.

## Results

### H2B.8 occupies silent genes marked by H3K27me3 and H2A.Z

**Fig. 1:**
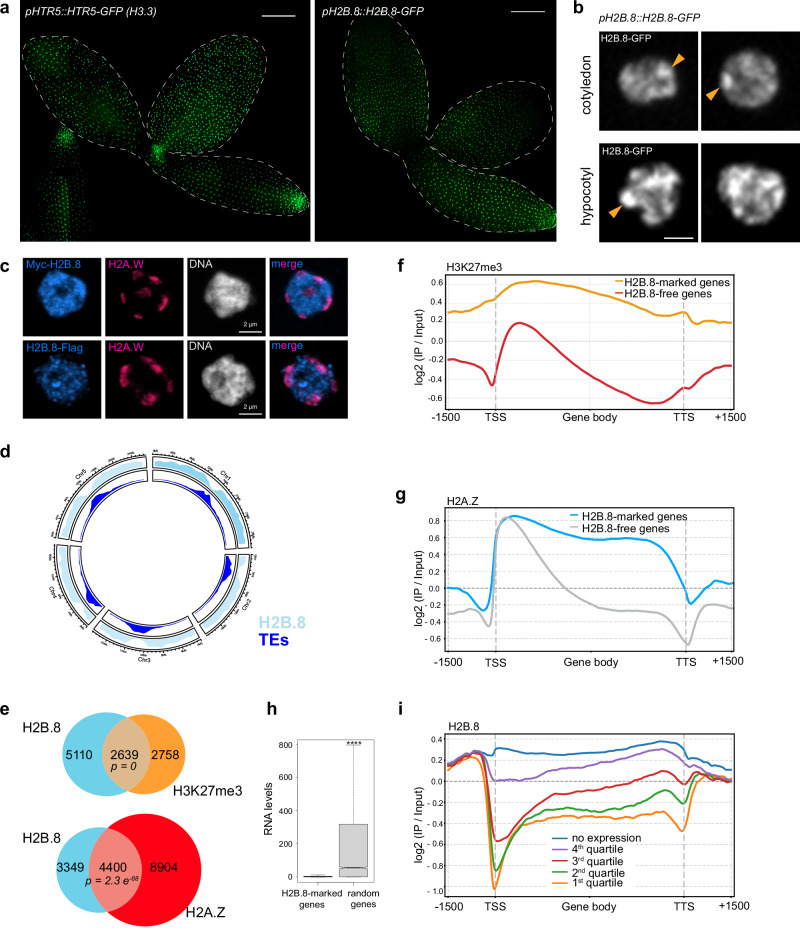
H2B.8 marks silent genes enriched in H3K27me3 and gene-body H2A.Z. **a** Maximum intensity projections of dissected dry seed (DS) embryos expressing H3.3 (HTR5) or H2B.8 as a GFP fusion protein under the respective endogenous promoters. Embryos were dissected from the seed coat and imaged with a Spinning confocal microscope, 25x objective, and mosaics stitched together using the Zeiss software. Scale bar represents 100 μm. **b** Selected DS nuclei from the *pH2B.8::H2B.8-GFP* transgenic line imaged with a Spinning confocal microscope using a 63x objective. Arrowheads mark nuclear domains enriched in H2B.8-GFP both in cotyledons (top) and hypocotyl nuclei (bottom). Scale bars correspond to 2 μm. **c** Isolated DS embryo nuclei stained with DAPI (grey), in which H2A.W (magenta) and H2B.8 (blue) as a C-terminal Flag or N-terminal Myc fusion were revealed by immunostaining. Scale bars correspond to 2 μm. **d** Circos plot showing enrichment in H2B.8 (sky-blue) along the 5 chromosomes and relative to the centromeric/pericentromeric regions enriched in transposable elements (dark blue). **e** Venn diagrams revealing a significant overlap between H2B.8-marked genes and genes enriched in H3K27me3 or H2A.Z in DS; p-values were determined with a one-sided hypergeometric test. Metagene plots of H3K27me3 (**f**) and H2A.Z (**g**) distribution over H2B.8-marked genes (*n* = 7749 genes) and an equivalent number of random H2B.8-free genes. **h** Mean mRNA levels (DESeq2 normalization) from 3 biological replicates of H2B.8-enriched genes and an equivalent number (*n* = 7546 genes) of random H2B.8-free genes, determined by RNA-seq in DS, **** p < 0.0001, two-tailed Mann Whitney test. The boxplot is centered on the median, the box boundaries encompass the second and third quartile, and the whiskers present the 1.5x interquartile range. **i** Metagene plot showing profiles of H2B.8 enrichment over silent (absence of reads) and genes with different expression levels in DS.

To study the distribution of the H2B.8 histone variant in dry seeds, we generated transgenic plants that express H2B.8-GFP under the control of its endogenous promoter. Like H3.3-GFP^29^, H2B.8-GFP is highly visible in the majority of the small nuclei in dry seed embryos, except in the shoot apical meristem (Fig. 1a). In both cotyledons and hypocotyls, H2B.8 is present throughout the nucleus and in H2B.8-enriched chromatin domains (Fig. 1b, Supplementary Fig. 1a-b). Given the size and localization of some of these H2B.8-enriched domains at the nuclear periphery, they may coincide with chromocenters that comprise pericentromeric heterochromatin and nucleolus organizer regions (NORs). To investigate this and ensure that the genomic distribution of H2B.8 was not affected by the position of the epitope tag, we generated additional transgenic lines, in which the variant is fused either to an N-terminal Myc tag or to a C-terminal Flag tag. Simultaneous detection by immunostaining of the H2B.8 and the H2A.W histone variants, the latter marking pericentromeric heterochromatin^21^, in isolated nuclei from dry seed embryos, revealed that H2B.8 is excluded from the H2A.W-enriched domains (Fig. 1c, Supplementary Fig. 1c-d). In dry seeds, H2B.8 is therefore enriched in larger euchromatic domains, while being globally depleted from pericentromeric heterochromatin.

To more precisely determine the genomic localization of this histone variant in dry seeds, we carried out ChIP-seq using a transgenic line that expresses Myc-H2B.8 under the control of its endogenous promoter. We obtained a set of common peaks from two independent replicates obtained with two different anti-Myc antibodies (Supplementary Fig. 2a). H2B.8 peaks occupy approximately 14.6% of the genome in dry seed embryos. Consistent with the immunofluorescence staining, H2B.8 is predominantly found along chromosome arms and is depleted from centromeric and pericentromeric heterochromatin (Fig. 1d). Despite the depletion of H2B.8 from the transposon (TE)-rich pericentromeric heterochromatin, 8713 TEs on the chromosome arms show H2B.8 occupancy, with no preference for DNA transposons or retrotransposons (Supplementary Fig. 2b). Besides TEs, we identified 7749 genes with H2B.8 peaks. Forty percent and 33% of these H2B.8-marked genes are shared, respectively, among H2B.8 targets in sperm cells and seedlings, which ectopically express H2B.8^25^, while the rest are seed-specific (Supplementary Fig. 2c, d). To determine whether H2B.8 colocalizes with other histone variants or specific histone modifications, we reanalyzed available ChIP-seq data from dry seeds^27,30^. A comparison of the ChIP-seq profiles showed that H2B.8 is globally depleted from genes marked by H3.3 or H3K4me3 peaks (Supplementary Fig. 2e). Instead, ~49% of the genes carrying the repressive Polycomb modification H3K27me3 are marked by H2B.8 (Fig. 1e-f); and ~57% of all H2B.8-marked genes show H2A.Z enrichment (Fig. 1e). This partial co-localization of H2B.8 with H2A.Z or H3K27me3 was also observed by immunofluorescence staining in dry seed embryo nuclei (Supplementary Fig. 2f). Closer investigation of the H2A.Z distribution reveals that H2B.8-marked genes show H2A.Z enrichment within the gene body (Fig. 1g), a pattern that is associated with transcriptional repression^31,32^. In addition, H2B.8 is depleted from accessible regions determined by ATAC-seq^9^ (Supplementary Fig. 2g).

Given the co-enrichment of H2B.8-marked genes with H3K27me3 and gene-body H2A.Z, we expected H2B.8 to label genes that were transcriptionally repressed during seed maturation. To ascertain this, we analyzed the stored mRNA pool of wild type (WT) dry seeds. Comparing mRNAs from genes occupied by H2B.8 to a random set of genes, revealed that little to no stored mRNA is present in dry seeds for H2B.8-marked genes (Fig. 1h). Consistently, metagene analyses of H2B.8 enrichment as a function of transcript levels confirm the depletion of H2B.8 from genes, for which mRNA accumulated during seed maturation (Fig. 1i, Supplementary Fig. 2h). Taken together, H2B.8 marks TEs on chromosome arms and repressed genes enriched in H3K27me3 and/or gene-body H2A.Z.

### H2B.8 forms predominantly heterotypic nucleosomes

**Fig. 2:**
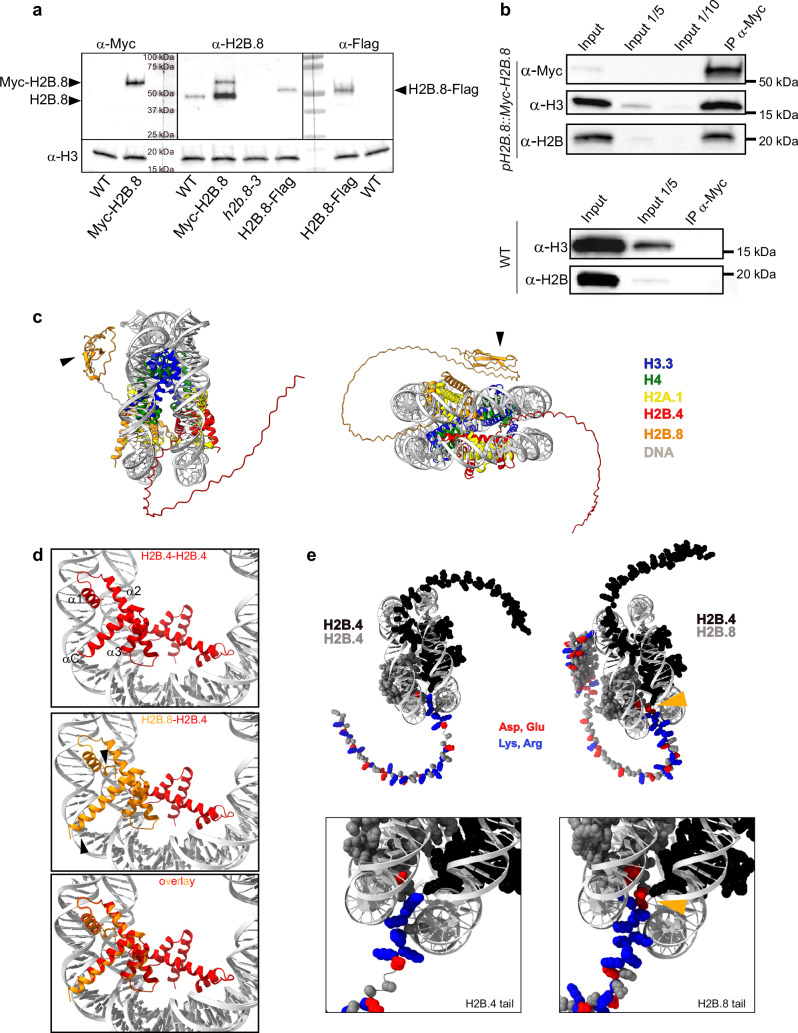
H2B.8 mainly forms heterotypic nucleosomes. **a** Western blot using an *in house* generated anti-H2B.8 antibody. Nuclear extracts from seeds expressing Myc-H2B.8 or H2B.8-Flag under the endogenous *H2B.8* promoter in the WT background, *h2b.8-3* mutant and WT seeds. The anti-Myc antibody (left) detects Myc-tagged H2B.8 and the anti-Flag antibody (right) the Flag-tagged H2B.8. Myc-tagged H2B.8, Flag-tagged H2B.8 and endogenous H2B.8, revealed by the anti-H2B.8 antibody, are indicated with arrowheads. H3 serves as loading control. **b** Immunoprecipitation of nucleosomes with an anti-Myc antibody from dry seeds expressing Myc-H2B.8 (*top*) or WT dry seeds (*bottom*). Detection with anti-Myc, anti-H3 and anti-H2B antibody reveals the presence of H3 and smaller H2B variants in the Myc-H2B.8 containing nucleosomes. Input was also deposited as a 1/5 or 1/10 dilution. **c** Structure of the heterotypic nucleosome predicted by AlphaFold 3 comprising H3.3 (blue), H4 (green), H2A.1 (yellow), H2B.4 (red) and H2B.8 (orange). Part of the H2B.8 N-terminal tail (AA8-65) is predicted to fold into β-sheets (arrowheads). The N- and C-terminal tails of all histones except H2B.4 and H2B.8 have been hidden for clarity. **d** Sub-fraction of the predicted nucleosome structures with either two H2B.4 molecules (red, top) or one H2B.4 and one H2B.8 (red and orange, middle) and the overlay of the two structures (bottom). H2B.8 is predicted to form a longer α1 and an extended C-terminal helix (arrowheads). Only the α-helical, structured parts of the H2B histone variants are shown. **e** Sub-fraction of the predicted nucleosome structures with either two H2B.4 molecules (left) or one H2B.4 and one H2B.8 (right) centered on the H2B N-terminal tail that protrudes between the two DNA gyres. Acidic amino acids (ASP, GLU) and basic amino acids (LYS, ARG) are colored in red and blue respectively. The arrowhead indicates additional acidic amino acids in the H2B.8 tail.

To characterize endogenous H2B.8, we generated a polyclonal antibody against a peptide in the N-terminal tail specific to H2B.8 (Supplementary Fig. 3a). By western blot analysis, this antibody detects the endogenous protein in WT plants as well as the Myc- or Flag-tagged version of H2B.8 in transgenic lines (Fig. 2a). We noticed that both the endogenous as well as the epitope-tagged H2B.8 migrate at a considerably higher molecular weight than the predicted 27 kDa for the endogenous protein. To test whether the unexpected electrophoretic mobility is caused by post-translational modifications specifically set *in planta*^33^, we expressed full-length H2B.8 or only its N-terminal tail as Gal4-DNA binding domain fusions with a Myc-tag in yeast. Both fusion proteins migrate at a similar molecular weight and slower compared to the H2B.4 variant (Supplementary Fig. 3b). Apparent higher molecular weight was also confirmed when the tail sequence was expressed as a GST-fusion in bacteria (Supplementary Fig. 3c), revealing that the reduced electrophoretic mobility of H2B.8 in dry seeds is conferred by the tail sequence rather than by post-translational modifications.

The incorporation of different histone variants into nucleosomes results in important combinatorial complexity^13^. However, not all combinations are favorable as for example H2A variants form exclusively homotypic nucleosomes^34^. To investigate whether H2B.8 nucleosomes in the dry seed are homotypic (two H2B.8 molecules per nucleosome) or heterotypic (one H2B.8 and another H2B variant), we immunoprecipitated nucleosomes from dry seeds expressing Myc-H2B.8. Mononucleosomes were released by MNase digestion (Supplementary Fig. 3d) and H2B.8-containing nucleosomes immunoprecipitated with an anti-Myc antibody. Histone H3 was detected only in the immunoprecipitated fraction from seeds expressing Myc-H2B.8, but not from WT seeds, showing that nucleosomes were successfully and specifically precipitated (Fig. 2b). Small H2B variants were detected by a generic H2B antibody in a similar immunoprecipitate to input ratio as H3, thus indicating that H2B.8 mostly forms heterotypic nucleosomes, in which H2B.8 is associated with one of the shorter H2B variants.

Furthermore, using seeds expressing both Myc-H2B.8 and a Flag-tagged version of H2B.4, we could reveal Myc-H2B.8 in immunoprecipitated Flag-H2B.4 nucleosomes (Supplementary Fig. 3e-g). Based on this observation, we used AlphaFold 3 to predict structures of nucleosomes comprising a 146 bp long Widom 601 sequence, H3.3, H4, H2A.1, H2B.4 or H2B.8 (Fig. 2c). We chose H3.3, H2A.1 and H2B.4 as these variants are highly expressed in seeds (refs. ^27,28^ and Supplementary Figs. 3h and 5a). AlphaFold predicts, although with low confidence, the presence of a small, structured domain with β-sheets in the very N-terminal region of H2B.8 (AA8-65, Fig. 2c) within the otherwise intrinsically disordered N-terminal tail (AA66-145). Other differences of H2B.8 compared to the other 10 H2B variants include changes from basic to acidic amino acids within the second and third α-helices (Supplementary Fig. 3a), which form contacts in the H2A-H2B dimer and the H4-H2B four-helix bundle^35^ and the presence of an extended C-terminal tail. We therefore analyzed the histone fold domain in the predicted nucleosomes comprising either H2B.4 and H2B.8 or two H2B.4 molecules. Compared to H2B.4, H2B.8 forms a longer structured α1 helix as well as an extended C-terminal α-helix (Fig. 2d). Finally, just N-terminally of the first α-helix and before a longer stretch of basic lysines and arginines, H2B.8 comprises an insertion of 5 amino acids including two acidic amino acids (Supplementary Fig. 3a), which are situated between the two DNA gyres (Fig. 2e*)*.

Thus, H2B.8 in dry seeds forms mostly heterotypic nucleosomes. Compared to nucleosomes containing only the shorter H2B variants, H2B.8-specific features are predicted to lead to distinct histone-histone or histone-DNA interactions in these heterotypic nucleosomes.

### H2B.8 influences 3D chromatin organization in dry seeds

**Fig. 3:**
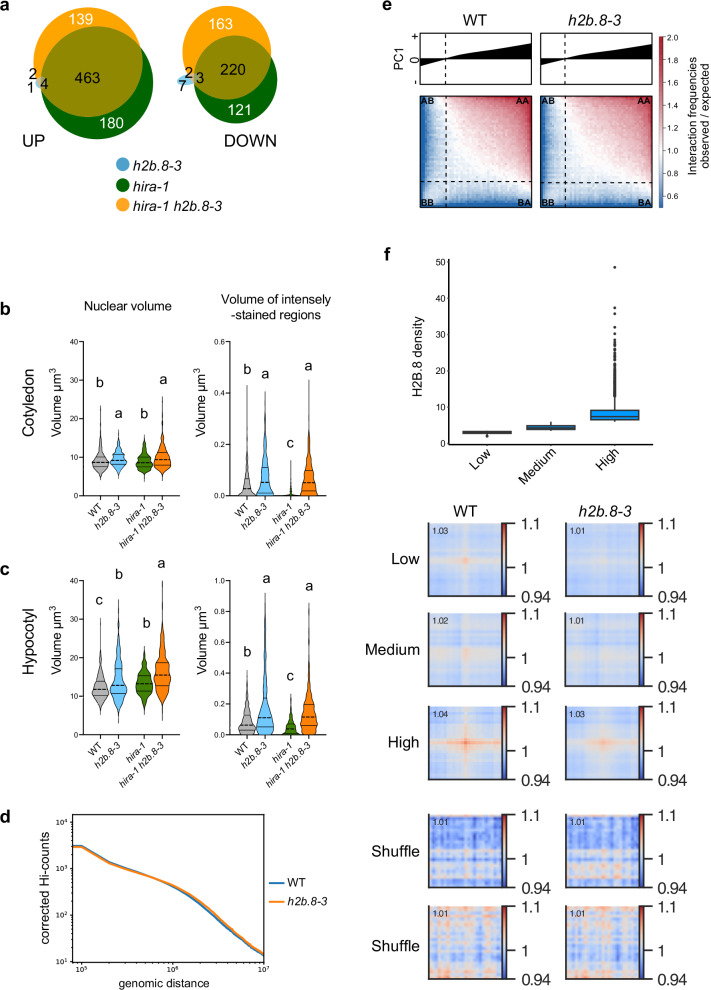
H2B.8 regulates 3D chromatin organization in dry seeds. **a** Venn diagram showing up- and downregulated genes ( | *log2FC* | > *1, padj* < *0,05*) in DS of *h2b.8-3*, *hira-1* and the *hira-1 h2b.8-3* double mutants relative to WT. Significant overlap between misregulated genes in *hira-1* and *hira-1 h2b.8-3* were determined with a one-sided hypergeometric test; *p* = *0*, for down**-**regulated genes, *p* = *0*, for up-regulated genes. Nuclear volume and total volume of intensely stained chromatin regions per nucleus in cotyledon (**b**) and hypocotyl nuclei (**c**) in DS embryos. Cotyledons: WT: *n* (number of nuclei) = 420, *N* (number of embryos) = 21, *h2b.8-3*: *n* = 260, *N* = 13, *hira-1*: *n* = 300, *N* = 15 and *hira-1 h2b.8-3*: *n* = 500, *N* = 25. Hypocotyls: WT: *n* = 440, *N* = 22, *h2b.8-3*: *n* = 300, *N* = 15; *hira-1*: *n* = 340, *N* = 17 and *hira-1 h2b.8-3*: *n* = 415, *N* = 21. Significant differences (*p* < 0.05) between genotypes were determined using a Kruskal-Wallis test coupled to a Dunn’s multiple comparisons test. The exact *p*-values are given in the source data file. **d** Averaged scaling plots with a 100 kb genomic bin size, illustrating how interaction frequencies vary with increasing genomic distance across all chromosomes. **e** Saddle plots showing chromatin compartmentalization in WT and *h2b.8-3* DS. The mean cis observed/expected interaction frequencies between 20 kb bins are plotted, ordered by first principal component (PC1) values from WT. Positive PC1 values represent A compartment regions (red), and negative values represent B compartment regions (blue). **f** Top: Boxplot of H2B.8 density showing three quantiles of H2B.8 peaks, classified as high, medium, and low H2B.8 enrichment based on fold change relative to input in ChIP-seq (mean values of 2 biological replicates). The boxplots are centered on the median, the box boundaries encompass the second and third quartile, and the whiskers present the 1.5x interquartile range. Bottom: Aggregate plots showing the mean contact matrices for WT and *h2b.8-3* at peaks grouped into three H2B.8 enrichment quantiles. Contacts in *h2b.8-3* are noticeably lower compared to WT. The two shuffle rows represent randomly selected genomic regions, serving as background controls. Source data are provided as a Source Data file.

To understand whether H2B.8 affects the transcriptome and shapes the organization of chromatin in dry seeds, we obtained a T-DNA insertion mutant, *h2b.8-3*, in which the T-DNA is situated in the exon, and which lacks the H2B.8 protein (Fig. 2a). After-ripened seeds of the *h2b.8* mutant germinate with kinetics comparable to WT under light (Supplementary Fig. 4a), when stimulated by Gibberellin or Ethylene in the dark (Supplementary Fig. 4b, c), under conditions that induce secondary dormancy, and after seed aging *(*Supplementary Fig. 4d, e). To determine whether H2B.8 deposition affects the stored mRNA pool in dry seeds, we analyzed the transcriptome by RNA-seq in single *h2b.8-3* mutants as well as in the *hira-1* mutant background, in which histone H3 occupancy is reduced^28^. Transcript levels of 991 genes are modified in the absence of HIRA (Fig. 3a), with 344 showing reduced and 647 genes increased transcript levels in the *hira-1* mutant, among the latter numerous histone genes (Supplementary Fig. 5a). Deficient nucleosome assembly therefore affects transcriptional regulation during seed maturation and consequently the stored mRNA pool. Instead, transcript levels of genes, including H2B.8-marked genes, remain unaffected by the loss of H2B.8, as observed in sperm nuclei^25^ (Fig. 3a, Supplementary Fig. 5b). The transcriptome of the *hira-1 h2b.8-3* double mutants strongly resembles that of the *hira-1* single mutant, which is consistent with the observation that H2B.8 is dispensable for the increased dormancy observed upon loss of the histone chaperone HIRA^28^ (Fig. 3a, Supplementary Fig. 5c, d, Supplementary Fig. 4f). Therefore, while deficient H3.3 deposition alters the dry seed transcriptome, H2B.8 deposition is dispensable for the establishment of the mRNA pool in dry seeds and seed germination.

Given that H2B.8 contributes to chromatin condensation in sperm nuclei^25^, we then sought to investigate whether H2B.8 incorporation shapes the nuclear morphology and influences higher-order chromatin organization in dry seed embryos. To quantify nuclear morphology and the condensed chromatin regions that stain intensely with DNA dyes, we obtained 3D images of cotyledon and hypocotyl nuclei from Hoechst-stained dry seed embryos of WT, *h2b.8-3, hira-1* and the *hira-1 h2b.8-3* double mutant. We trained a deep-learning model on a set of manually annotated images from embryos to segment nuclei and intensely stained chromatin regions (Supplementary Fig. 6a). While the shape of the cotyledon and hypocotyl nuclei remains unaltered in absence of H2B.8 (Supplementary Fig. 6b, c), we observed a modest increase in the volume of the hypocotyl nuclei in the *h2b.8* and *hira-1* mutants, as well as in both the cotyledons and the hypocotyls of seeds lacking both HIRA and H2B.8 (Fig. 3b, c*;* Supplementary Fig. 6b, c). Next, we segmented the intensely stained chromatin regions and determined their volume per nucleus. Although fewer chromatin aggregates are detectable in *hira-1* mutants, the loss of H2B.8 in both the WT and the *hira-1* mutant backgrounds results in more discernible heterochromatin regions in cotyledon and hypocotyl nuclei compared to WT (Fig. 3b, c*;* Supplementary Fig. 6c). H2B.8 therefore contributes moderately to nuclear size, and its absence leads to altered chromatin organization in dry seeds. This effect is independent of major changes in chromatin accessibility, as determined by MNase digestion (Supplementary Fig. 7), and dominant over the microscopic changes in chromatin organization induced by loss of HIRA.

Next, to investigate the role of H2B.8 in organizing chromatin in dry seed nuclei in molecular detail, we generated high-resolution maps of 3D chromatin architecture in Arabidopsis by conducting Hi-C experiments from manually dissected WT and *h2b.8-3* mutant dry seed embryos (see Supplementary Data 1 and Methods). Genome-wide analyses of eigenvalues, as well as correlation analyses of the first principal component (PC1) values (Supplementary Fig. 8a) together with the visual inspection of Hi-C interaction maps (Supplementary Fig. 8b, c) confirmed strong consistency between biological replicates. Using cLoops2^36^, we estimated the Hi-C resolution to 5 kb for both control samples and the *h2b.8-3* mutant (Supplementary Fig. 9). To evaluate whether the loss of H2B.8 influences the global higher-order chromatin organization, we computed differential interaction maps, which showed no substantial changes in contact frequency in the mutant compared to the WT dry seeds (Supplementary Fig. 10). As chromatin interaction frequencies typically decrease with genomic distance according to a power-law function^37^, we generated scaling plots for both genotypes, which revealed no detectable differences in the decay of interactions over distance, indicating that both short- and long-range contacts remain largely unaltered upon loss of H2B.8 (Fig. 3d). Furthermore, comparison of first principal component (PC1) profiles between WT and *h2b.8-3* mutant samples showed no marked shifts between A (transcriptionally active) and B (inactive) compartment identities (Fig. 3e). Thus, global chromatin compartmentalization in dry seed embryos appears to be largely stable in the absence of functional H2B.8. Previous work in both plant and animal systems suggests that chromatin regions marked by similar histone modifications or bound by common regulatory factors preferentially associate in 3D space^38^, and H2B.8 has previously been shown to be involved in the condensation of chromatin in sperm nuclei^25^. To determine whether H2B.8 contributes to such homotypic interactions in dry seed embryos, we stratified H2B.8-enriched regions in WT samples into three categories (low, medium, and high) based on ChIP-seq signal intensity. We then constructed aggregate Hi-C submatrices to quantify the mean contact enrichment between regions similarly marked by H2B.8. Following observed versus expected normalization, we found that WT samples showed enriched contacts among H2B.8-marked loci, whereas this enrichment was reduced in the *h2b.8-3* mutant (Fig. 3f). Together with the microscopy analysis of dry seed nuclei, these findings suggest that H2B.8 plays a role in facilitating spatial proximity among specific euchromatin domains.

### H2B.8 is evicted upon imbibition and impacts transcriptional reprogramming

**Fig. 4:**
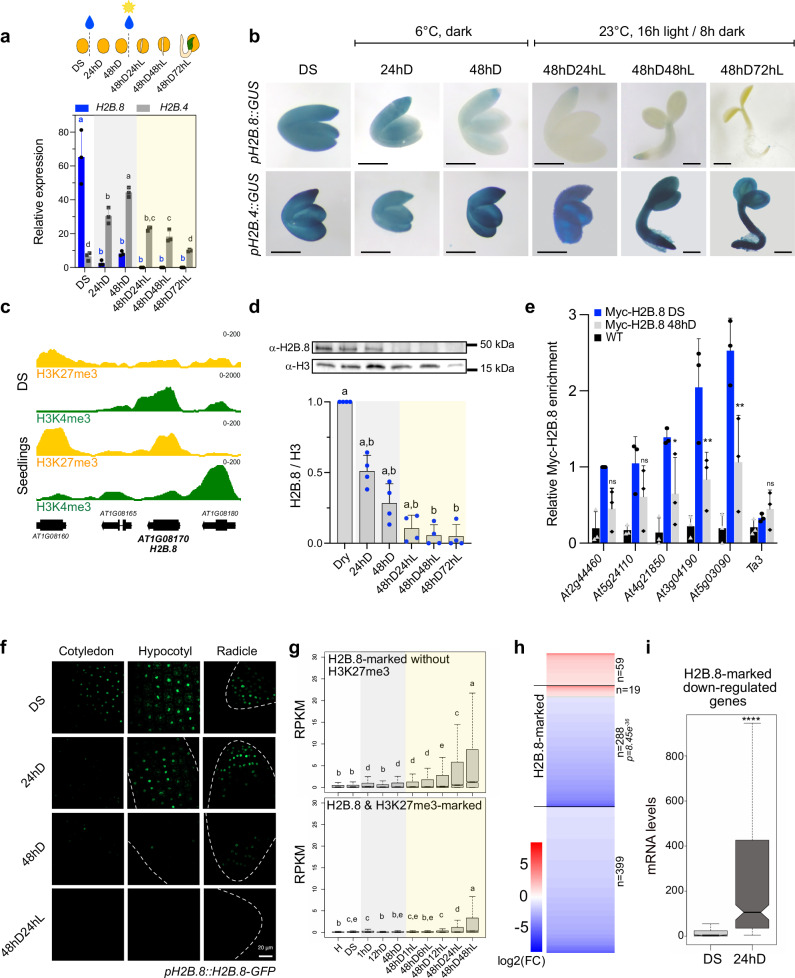
Seed imbibition triggers H2B.8 eviction. **a** Top: Scheme of experimental setup. Seeds were imbibed for 24 h or 48 h in the dark at 6 °C, before transfer to long-day conditions, at 23 °C. Bottom: Mean of *H2B.8* and *H2B.4* transcript levels normalized to *MON1* and *TIP41*, determined by RT-qPCR in DS, after 24 h or 48 h imbibition in the dark (24hD, 48hD, grey background) and in stratified seeds after 24h, 48h or 72 h of incubation under long days (48hD24hL - 48hD72hL, yellow background), hL, hours after transfer to long-day light. Letters indicate significant differences between time points for the gene in the respective color. One-way ANOVA with Tukey’s multiple comparisons test, *n* = 3 biological replicates, error bars represent SD. **b** GUS-stained embryos in transgenic lines expressing the *GUS* coding sequence under control of the *H2B.8* (top) or the *H2B.4* (bottom) promoter. Time points as in **a**. Scale bar represents 0.5 mm. **c** Genome browser view of H3K4me3 and H3K27me3 at the *H2B.8* (*At1g08170*) locus in DS and 8-day old seedlings (ChIP-seq data from refs. ^30,75^). **d** Representative western blots of H2B.8 and the H3 loading control (top). Bar plot (bottom) represents mean endogenous H2B.8 levels normalized to H3 from 4 independent biological replicates. Relative H2B.8 levels in DS were set to 1. Letters indicate significant differences between time points, Kruskal-Wallis test with Dunn’s multiple comparisons test. Error bars represent SD. **e** Myc-H2B.8 enrichment relative to input in DS and 48hD imbibed seeds at five H2B.8-marked genes and the pericentromeric retrotransposon *Ta3* with low H2B.8 levels. H2B.8 levels at *At2g44460* in DS were set to 1 in each biological replicate. WT dry seeds not expressing Myc-H2B.8 were used as background control. Bar plot shows mean of 3 biological replicates and error bars represent SD. Two-sided unpaired ttest comparing DS with 48hD for each gene except for *At2g44460*, for which a two-tailed Wilcoxon Signed Rank Test was used; *At4g21850* *p = 0.0581; *At3g04190* ** *p* = 0.0456; At5g03090 ** *p* = 0.0276**. f** Representative maximum intensity projections of cotyledons, hypocotyls or radicles from embryos expressing H2B.8-GFP under control of its endogenous promoter. Embryos were dissected from DS, imbibed and germinating seeds at 24hD, 48hD and 48hD24hL and imaged with a Spinning confocal microscope, 63x objective. Scale bar represents 20 μm. **g** Mean transcript levels in RPKM from 3 biological replicates of genes marked by H2B.8 but not H3K27me3 (*n* = 4955 genes) or both H2B.8 and H3K27me3 (*n* = 2592 genes, H3K27me3 ChIP-seq dataset from ref. ^30^) at different developmental stages during the seed-to-seedling transition identified in the RNA-seq dataset from ref. ^8^. The boxplot is centered on the median, the box boundaries encompass the second and third quartile, and the whiskers present the 1.5x interquartile range. H = at harvest. Comparison of the same developmental stages between the two gene sets reveals significant differences at each time point, **** p < 0.0001, Kruskal-Wallis test coupled to a Dunn’s multiple comparisons test (not indicated in the figure). Letters indicate significant differences between time points within a gene set, determined by a Kruskal-Wallis test coupled to a Dunn’s multiple comparisons test. **h** Heat map showing up and down-regulated genes (*n* = 765 genes) after *spike-in* normalization in *h2b.8-3* mutants compared to WT at 24hD. 42% (288/687) of the down-regulated genes are marked by H2B.8. Significant overlap between downregulated and H2B.8-marked genes were determined with a one-sided hypergeometric test. **i** Comparison of mean transcript levels (3 biological replicates) of H2B.8-marked genes (*n* = 288 genes), which are down-regulated in *h2b.8-3* mutants at 24hD, from DS and 24hD imbibed WT seeds; **** p < 0.0001, two-tailed Mann-Whitney test. The boxplot is centered on the median, the box boundaries encompass the second and third quartile, and the whiskers present the 1.5x interquartile range. The exact *p*-values for **a,** **d, e** and **g** are given as source data, provided as a Source Data file.

Given that H2B.8 is enriched in the chromatin of dry seed embryos and contributes to its higher-order organization, our subsequent objective was to investigate the dynamics of this variant during seed germination. To this end, the expression and localization of H2B.8 was monitored. Our experimental setup involved imbibing seeds for 48 hours at 6 °C in the dark, followed by germination in a growth chamber set to long-day conditions. In this setup, the first radicle protrusion was observed around 48 h after transfer to light (Supplementary Fig. 4a). As soon as 24 h after contact with water, *H2B.8* transcript levels are strongly reduced (Fig. 4a*;* Supplementary Fig. 11a). To characterize the *H2B.8* promoter activity in more detail, we established transgenic plants that express the *β-glucuronidase (GUS*) reporter gene from the *H2B.4* (control) or the *H2B.8* promoter and followed GUS activity using X-Gluc histochemical staining. The control construct driven by the *H2B.4* promoter shows that this promoter was active during maturation and results in strong *GUS* expression throughout germination (Fig. 4b*;* Supplementary Fig. 11a). In contrast, the *GUS* gene driven by the *H2B.8* promoter is progressively downregulated during seed imbibition, with the loss of blue staining initiating in the cotyledons. After 24 h in the light, only faint staining remains visible in the radicle (Fig. 4b). The progressive downregulation of the *H2B.8* gene correlates with the transition from a H3K4me3-rich chromatin state in dry seeds to a H3K27me3-marked chromatin state of the *H2B.8* gene in seedlings (Fig. 4c). Next, to investigate the fate of the H2B.8 histone variant during the seed-to-seedling transition, we carried out western blot analyses. Both Myc-tagged H2B.8 levels (Supplementary Fig. 11b) and endogenous protein levels (Fig. 4d) decrease by up to 50% after 24 h imbibition in the cold, indicating eviction and degradation of the H2B.8 protein. The dynamics of H2B.8 contrast with the long half-life of several weeks of mammalian histones in non-proliferating cells^39,40^. To confirm the eviction of H2B.8 from chromatin, we quantified Myc-H2B.8 levels by ChIP-qPCR at several H2B.8 target genes (Fig. 4e). Then to endorse these results by a different method, we investigated H2B.8 dynamics during imbibition using live cell imaging. In dry seeds, GFP-tagged H2B.8 can be detected in the cotyledons, hypocotyls and most strongly in the radicle. After 24 h of imbibition, the number of H2B.8-positive nuclei starts to decrease, at 48hD faint H2B.8-GFP signals remain detectable in some root tips, and after 24 h in the light, H2B.8-GFP is undetectable (Fig. 4f, Supplementary Fig. 11c). Given the substantial co-enrichment of genes in H2B.8 and H3K27me3 in dry seeds *(*Fig. 1e-f*)*, we asked whether H2B.8 occupancy or its subsequent eviction during imbibition could influences H3K27me3 reprogramming during the seed-to-seedling transition. However, no significant overlap between the genes that gain or lose H3K27me3^41^, and H2B.8 occupancy in dry seeds was detected (Supplementary Fig. 12a).

To study the global transcriptional dynamics of H2B.8-marked genes, we determined their expression patterns during the seed-to-seedling transition in an available RNA-seq dataset^8^. H2B.8-marked genes are globally expressed at low levels during germination and seedling establishment (Supplementary Fig. 12b). However, compared to genes marked by both H2B.8 and H3K27me3, a subset of the 4955 genes marked only by H2B.8 in this dataset^8^ is progressively activated during seed germination (Fig. 4g, Supplementary Fig. 12c). To determine whether the presence of H2B.8 in dry seeds or its dynamics during imbibition affect transcriptional reprogramming, we carried out *spike-in* RNA-seq analysis on WT and *h2b.8-3* mutant seeds harvested at 24 hD. We find that 765 genes exhibit differential expression in imbibed *h2b.8-3* mutant seeds (Fig. 4h, Supplementary Fig. 12d), of which the majority (687) are downregulated compared to WT. Notably, 42% (288) of the downregulated genes are marked by H2B.8 in dry seeds (Fig. 4h). Furthermore, H2B.8-marked genes showed a more pronounced reduction in transcript levels than H2B.8-free genes (Supplementary Fig. 12e), are globally induced during imbibition (Fig. 4i) and enriched among genes involved in hypoxia and low oxygen response (Supplementary Fig. 12f). Together, this indicates that the transcriptional activation of a subset of H2B.8-marked genes is influenced by the enrichment in this histone variant, either by pre-marking these genes for activation or by facilitating their activation through H2B.8 eviction. Therefore, concomitant with transcriptional reprogramming, seed imbibition triggers the degradation of *H2B.8* transcripts and the eviction of the H2B.8 histone variant from chromatin revealing that water uptake initiates chromatin reprogramming.

### Histone eviction during imbibition is not restricted to H2B.8

**Fig. 5:**
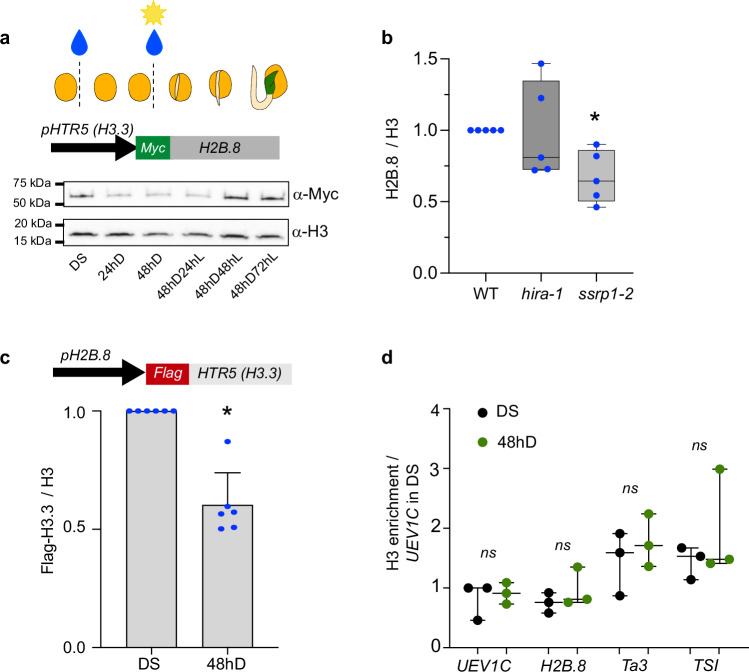
Histone eviction is not restricted to H2B.8. **a** Representative western blot showing expression of Myc-tagged H2B.8 under control of the *HTR5 (H3.3)* promoter during the seed-to-seedling transition together with the H3 loading control. Time points are the same as in Fig. 4. D, dark; hL, hours after transfer to long-day light. **b** Quantification of endogenous nuclear H2B.8 proteins relative to H3 in WT, *hira-1* and *ssrp1-2* mutant seeds. Graph shows mean of 5 biological replicates. * p = 0.0537, two-sided non-parametric Friedman test coupled to a Dunn’s multiple comparisons test. The boxplot boundaries extend from the 25th to 75th percentiles, the line represents the median and the whiskers extend from the smallest to the largest value. **c** Quantification, relative to total H3, of Flag-H3.3 expressed under the *H2B.8* promoter in DS and after 48 h imbibition in the dark (48hD). Graph shows mean of 6 independent transgenic lines. * p = 0.0312, two-tailed non-parametric Wilcoxon Signed Rank test. Error bars correspond to SD. Samples derived from the same experiments were run on two different gels and processed in parallel with either the anti-Flag or the anti-H3 antibody. **d** H3 ChIP-qPCR analysis of relative H3 enrichment (*n* = 3 biological replicates) in DS and seeds stratified for 48h in the dark (48hD). H3 enrichment values were normalized to input and to the H3 levels at the *UEV1C (At2g36060)* gene in DS, *ns, not significant*, two-tailed ttest and two-tailed Wilcoxon Signed Rank test for *UEV1C*. The whiskers extend from the smallest to the largest value. Source data are provided as a Source Data file.

Given the deposition and eviction patterns of H2B.8 histones, their assembly into nucleosomes may involve specific histone chaperone machineries that are expressed specifically during seed maturation. To investigate this possibility, we expressed Myc-H2B.8 under the control of the *HTR5 (H3.3)* promoter and monitored H2B.8 protein levels during the seed-to-seedling transition. Similar to the expression of H2B.8, driven by the 35S promoter^25^, ectopic H2B.8 expression under control of the *HTR5* promoter does not cause any phenotypic abnormalities in plants. Western blots of protein extracts from seeds ectopically expressing H2B.8 showed that while H2B.8 is initially evicted during imbibition, this variant can be re-deposited during germination and seedling establishment when continuously expressed (Fig. 5a). This demonstrates that its incorporation is mediated by ubiquitous histone chaperone systems. One histone chaperone that handles H2A-H2B histones is FACILITATES CHROMATIN TRANSCRIPTION (FACT)^42^. To determine whether FACT plays a role in H2B.8 dynamics, we examined H2B.8 levels in WT, *hira-1* and *ssrp1-2* mutants, which express a truncated SSRP1 subunit of the FACT complex^43,44^. In dry seeds, the relative levels of H2B.8 protein normalized to H3 are reduced in the *ssrp1-2*, but not in the *hira-1* mutant compared to the WT (Fig. 5b), revealing that FACT is important for H2B.8 deposition or its maintenance in dry seeds.

Considering the observed eviction and degradation of H2B.8 during imbibition, we then asked whether this eviction is representative of a more global chromatin reprogramming or whether it is restricted to H2B.8. To this end, transgenic lines were established, which express Flag-H3.3 under control of the *H2B.8* promoter. We expect that in these lines, following the immediate downregulation of the *H2B.8* promoter (Fig. 4a-c) little to no new Flag-H3.3 will be produced upon imbibition. In consequence, the dynamics of the *‘seed’* H3.3 pool, deposited during seed maturation, can be followed. We find that, in multiple independent transgenic lines, Flag-H3.3 levels were decreased 48 hours after imbibition in comparison to the dry seed (Fig. 5c*)* indicating that histone eviction during imbibition is not exclusive to H2B.8. To investigate whether the decrease in *‘seed’* H3.3 results in altered nucleosome occupancy, we conducted H3-ChIP-qPCR in dry and imbibed seeds at 48hD. At the analyzed loci, there is no evidence of reduced nucleosome occupancy at 48hD compared to dry seeds (Fig. 5d), therefore suggesting that loss of *‘seed’* H3.3 is compensated by deposition of new H3 histones. In conclusion, seed imbibition is characterized by important histone eviction and de novo deposition, indicating substantial chromatin reprogramming during the initial stages of seed germination.

### Histone H2B.8 eviction takes place in a DNA-replication independent manner but requires de novo transcription

**Fig. 6:**
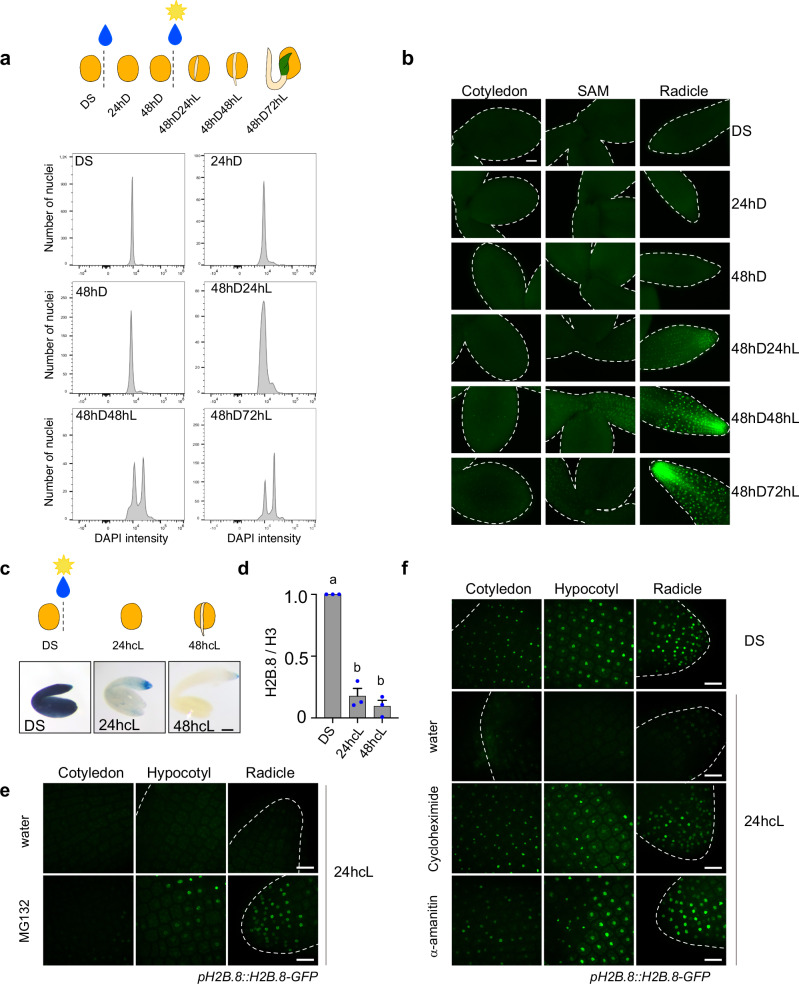
H2B.8 eviction during imbibition takes place in a DNA-replication independent manner but requires transcription. **a** Flow cytometry analysis of nuclei from isolated embryos at the indicated stages during the seed-to-seedling transition. Time points are the same as in Fig. 4. D, dark; hL, hours after transfer to long-day light. **b** EdU incorporation revealed by Click-IT chemistry in embryos at different stages during the seed-to-seedling transition. Images were acquired with a Spinning confocal microscope, equipped with a 25x objective. Scale bars correspond to 50 μm. **c** β-glucuronidase (GUS) histochemical assay revealing activity of the *H2B.8* promoter in transgenic lines expressing *GUS* under control of the *H2B.8* promoter in DS, and after 24 h or 48 h incubation under continuous light (24hcL, 48hcL); cL, continuous light. Scale bar represents 0.2 mm. **d** Quantification from western blots of endogenous, nuclear H2B.8 levels normalized to H3 in DS, at 24hcL and 48hcL. Bar plot shows mean of 3 biological replicates and error bars represent SD. Letters indicate significant differences between time points. One-way ANOVA with Tukey’s multiple comparisons test on raw values. DS vs 24hcL *p* = 0.0147, DS vs 48hcL *p* = 0.0089. **e**,** f** Representative maximum intensity projections of cotyledons, hypocotyls and radicles from embryos expressing H2B.8 as a GFP fusion under its endogenous promoter isolated from dry seeds or imbibed seeds under continuous light (24hcL). Images were acquired with a Spinning confocal microscope, 63x objective using identical exposure settings for treated and untreated samples. Seeds were imbibed in water or in the presence of 50 µM MG132, 100 µM Cycloheximide or 500 µM α-amanitin. Scale bars correspond to 20 μm.

The process of DNA replication provides an opportunity to modify chromatin structure, as new histones are deposited concurrently with DNA replication. The progressive loss of *‘seed’* histones (Figs. 4d-f and 5c) may therefore be attributed to the dilution of the existing histones by the newly deposited histones during the process of DNA replication. To investigate whether the chromatin reprogramming during imbibition is replication-coupled, we timed the onset of DNA replication in embryos in our experimental setup. Flow cytometry analysis of dissected embryos at different time points during the seed-to-seedling transition revealed one sharp peak in dry seed embryos, showing that most nuclei are arrested in the G1 phase (Fig. 6a). The initial broadening of the peak and the emergence of S-phase nuclei at 24 hours after transfer to light indicate that DNA (endo)replication initiates between 48hD and 48hD24hL (Fig. 6a, Supplementary Fig. 13a, b) before a distinct G2 peak emerges by 48hD48hL. We confirmed the onset of DNA replication by EdU staining, which revealed the first EdU-positive nuclei at 48hD24hL, predominantly in the radicle (Fig. 6b, Supplementary Fig. 13c). The decline in H2B.8 levels ( ≤ 48hD) therefore occurs before the first detectable EdU-positive nuclei (48hD24hL), thereby preceding DNA replication. Consequently, chromatin reprogramming, which leads to the eviction of H2B.8 during the first 48h of imbibition (Figs. 4d-f and  5c) occurs independently of DNA replication.

Based on the dynamics of H3.3 and H2B.8 eviction, we hypothesized that chromatin reprogramming reflects the physiological state of the seed. To test this, we imbibed seeds in continuous light (cL). Under these conditions, 25 to 57% of the seeds germinated after 48 h across the three biological replicates (Supplementary Fig. 14a). GUS staining was diminished at 24hcL compared to 24hD (Figs. 6c and 4b*)* revealing *H2B.8* promoter inactivation within 24hcL. Western blots further revealed that about 80% of H2B.8 proteins are evicted and degraded after 24hcL (Fig. 6d). The differences observed in H2B.8 eviction between imbibition in the dark/cold versus light/23 °C conditions provide evidence that chromatin reprogramming occurs concomitantly with the physiological alterations during the seed-to-seedling transition. We then tested whether H2B.8 degradation involves the proteasome pathway. We therefore treated seeds expressing H2B.8-GFP with proteasome inhibitors and found that H2B.8-GFP was retained longer in the inhibitor-treated seeds (Fig. 6e, Supplementary Fig. 14b-d). Germination requires translation of stored mRNA, while de novo transcription has been shown not to be essential for radicle protrusion^5^. To test whether H2B.8 eviction during imbibition necessitates the capacity of the embryo to translate stored mRNA or to initiate de novo transcription, we treated seeds expressing H2B.8-GFP with the translational inhibitor cycloheximide or with α-amanitin, which inhibits RNA Polymerase II activity. Both treatments impeded H2B.8 eviction and degradation (Fig. 6f, Supplementary Fig. 14b, e-g), while cycloheximide also affected seed viability as evidenced by the absence of 2,3,5-triphenyl tetrazolium chloride (TTC) staining in treated seeds (Supplementary Fig. 14h). These results therefore indicate that proteasome activity and global transcriptional onset upon water uptake contribute to the observed replication-independent chromatin reprogramming in the embryo during imbibition.

## Discussion

Dry seed embryos in Arabidopsis have small nuclei with highly condensed chromatin^6^. Here, we demonstrate that these nuclei are characterized by the presence of the H2B.8 histone variant. H2B.8 accumulates in transcriptionally repressed chromatin regions that cluster in 3D nuclear space and is evicted in a replication-independent manner upon water uptake during imbibition. Expressed late during seed maturation, H2B.8 accumulates in dry seeds at TEs located on chromosome arms, as well as at genes with H3K27me3, and H2A.Z enrichment in the gene body, suggesting the existence of locus-specific deposition mechanisms. Given that H2B.8 is expressed during the maturation phase^45^, by the time proliferation has ceased^46^, and accumulates at silent loci, replication- and transcription-coupled H2B.8 deposition are unlikely to be the predominant modes of H2B.8 chromatin assembly. Instead, the observed H2B.8 enrichment patterns in dry seeds may reflect its accumulation in chromosomal regions that are devoid of active transcription but undergo histone H2A-H2B exchange catalyzed by chromatin remodeling factors, similar to the deposition of H2A.Z, which relies on the SWR1 chromatin remodeling complex^47^, or H2A.W, which requires DECREASE IN DNA METHYLATION1^48,49^. Reduced H2B.8 levels in a mutant of the histone chaperone complex FACT, known to reorganize nucleosomes by disrupting histone-histone and DNA-histone interactions and by depositing new or recycled histone H2A-H2B dimers^50,51^, suggests a role for FACT in H2B.8 maintenance during seed maturation. Furthermore, the exclusion of H2B.8 from the pericentromeric regions marked by H2A.W variants and its co-enrichment with H2A.Z may indicate that specific histone variant combinations within a nucleosome are favored.

H2B.8 differs from the other H2B variants in several sequence features, and its incorporation is therefore likely to affect nucleosome structural properties. Modified nucleosome properties due to H2B variant incorporation have been observed for different H2B variants in other organisms, e.g. for the primate-specific H2B.W1 variant, which is expressed in spermatogonia. Like H2B.8, H2B.W1 has an extended C-terminal tail, and it exhibits weakened histone-DNA interactions^52^. Another example is H2BE, which is expressed in the olfactory epithelium of the mouse brain^53,54^, where it increases chromatin accessibility, a property conferred by a single amino acid change (V39I) in the α1 helix at the interface between the histone octamer and DNA^54^. At this position, H2B.8 carries an arginine residue (I153R, Supplementary Fig. 15) that points toward the DNA, suggesting stronger DNA-histone interactions. Future in vitro studies should clarify the extent to which H2B.8 incorporation affects nucleosome stability or DNA unwrapping properties in different nucleosomal contexts.

A common feature of sperm and dry seed nuclei that express H2B.8 is their small size^6,25^, suggesting that H2B.8 could contribute to the tight chromatin organization in seeds through its phase separation properties^25^. In dry seeds, loss of H2B.8 only moderately affects the volume of embryonic nuclei. Therefore, incorporation of H2B.8 is not the primary factor in chromatin condensation during seed maturation, and changes in nuclear volume during germination are likely not regulated by the eviction of H2B.8, but rather by the nuclear lamina components CROWDED NUCLEI (CRWN), which are required for the increase in nuclear volume during germination^6^. Instead, H2B.8 incorporation contributes to the higher-order organization of chromatin in embryonic nuclei. Large H2B.8-enriched chromatin domains can be visualized in embryos both by live-cell imaging and immunofluorescence. Our Hi-C analysis revealed that H2B.8-rich domains tend to cluster within the 3D nuclear space. Although the global chromatin architecture is not markedly altered in the absence of H2B.8, the 3D clustering of H2B.8-enriched regions along chromosome arms is diminished in seeds lacking this histone variant. As a result, other higher-order chromatin domains, such as the H2B.8-depleted pericentromeric regions, may be more easily visualized by microscopy and segmented from the remaining condensed euchromatin.

As in sperm cells^25^, H2B.8 marks lowly expressed or silent genes in embryos, which is consistent with its co-enrichment with H3K27me3 and with H2A.Z in the gene body. Its genomic distribution is likely linked to its deposition mode as discussed above without however being required for the transcriptional repression of these genes. In seedlings, transcriptional activation correlates with the eviction of ectopically expressed H2B.8^25^. Therefore, we hypothesized that eviction of H2B.8 upon water uptake in embryos could facilitate transcriptional reactivation during imbibition. This hypothesis is supported by the observation that the transcriptional upregulation, during imbibition, of a subset of H2B.8-marked genes is affected in the absence of H2B.8.

Despite the evolutionary conservation of H2B.8 orthologs in angiosperms, which exhibit conserved expression patterns in seeds and pollen^23,26^, we were unable to reveal altered seed germination phenotypes for *h2b.8* mutants under the tested conditions. Furthermore, no altered phenotypes or gene expression defects were observed upon its ectopic expression under a strong viral promoter^25,33^ or under the *H3.3* promoter (*our study*). The lack of observable phenotypes under the tested conditions, is a limitation of our study. Further experiments, e.g. exploring mutants of *H2B.8* orthologs in other angiosperms or testing the seed germination of Arabidopsis mutants under hypoxia or other stresses may help to overcome this limitation. Under our standard growth conditions, chromatin plasticity may be sufficient to accommodate the loss of H2B.8 in seeds or the ectopic presence of this atypical variant, along with the potential changes in nucleosome stability induced by its incorporation into the chromatin. The absence of a germination phenotype under control conditions is consistent with the *h2b.8* mutants being fertile, and with the only subtle phenotypes in mutants of other histone variants, such as the sperm-specific H3.10^24^, H3.14^55^ or H3.15^56^ under standard growth conditions. Even though these variants show unique expression patterns and play a role in H3K27me3 reprogramming or stress responses, they are dispensable for plant viability and fertility. This raises the question of what drives the conservation of the H2B.8 variant and its specific expression patterns in seeds and pollen throughout angiosperm evolution. Selection pressure may primarily act on sperm cells, where H2B.8 incorporation has been shown to reduce the nuclear volume, facilitating the migration of sperm nuclei through the pollen tube^25^. Alternatively, specific seed germination phenotypes may only be observed in nature. In contrast to laboratory conditions, seeds in nature are constantly exposed to repeated imbibition-desiccation cycles, and mother plants are exposed to different environmental stresses during seed maturation. Under these conditions, H2B.8 deposition may vary and contribute to the adjustment of the transcriptional program during imbibition. Indeed, when seeds are imbibed under suboptimal conditions and enter secondary dormancy, *H2B.8* transcript levels increase^57^ suggesting that H2B.8 accumulation can be fine-tuned in response to environmental conditions.

Upon water uptake, *H2B.8* transcript levels decline and the H2B.8 histone variants are evicted and degraded, a process that involves the proteasome pathway. Eviction occurs first in the cotyledon tissue and the hypocotyl, while H2B.8 is retained longer in the root meristem area. This eviction takes place initially in a replication-independent manner and is not restricted to the H2B.8 histone variant. Levels of tagged H3.3, deposited during seed maturation, similarly decline, while nucleosome occupancy remains unaffected, revealing global histone turnover in non-replicating cells during the imbibition step. However, further experiments are needed to determine whether H3.3 is simply evicted or if part of the *‘seed’* H3.3 is recycled or redeposited at different loci. After 48 h of imbibition, the levels of H2B.8 and H3.3 variants that were initially present in dry seeds decreased to approximately 50% and 60%, respectively. These histone loss rates are similar to those observed in alfalfa cell cultures^58^, yet they contrast with the substantially longer histone lifetimes of over 100 days seen in non-proliferating mammalian liver and brain cells^39^ or the short histone lifetimes of ~4 hours in mouse embryonic fibroblasts^59^. These estimations are global at the cellular or tissue level, but differences among genomic regions depending on their transcriptional activity are expected. Indeed, the half-life of H3.3 ranged from 1-1.5 hours at genes with high nucleosome turnover in Drosophila S2 culture cells^60^ and from 12-24 h at transcribed genes in Arabidopsis^61^. The histone eviction observed in this study depends on the physiological state of the seed, as evidenced by the accelerated eviction of H2B.8, when seeds are imbibed under continuous light. Subsequent studies should elucidate whether histone turnover during seed imbibition varies among genomic loci and whether it is driven by transcriptional activity. Global histone turnover is not restricted to the seed-to-seedling transition but has also been observed in the G2 cells of roots during the final cell cycle before the exit from proliferation, where H3.1 is evicted and replaced by H3.3^62^, and after fertilization, when paternal H3.3 variants are actively removed^29^. Different mechanisms could lead to the observed histone turnover. Our data show a requirement for transcription and proteasome activity, suggesting that histone turnover is an active process. Enhanced chromatin remodeling activity or global transcriptional restart upon imbibition through the action of de novo expressed proteins may favor H2B.8 eviction. A potential outcome of histone turnover is the setting of new post-translational modifications. Replacing H2B.8 by shorter H2B variants that can be mono-ubiquitinylated at the C-terminal lysine, may favor H2Bub enrichment, which has been linked to gene expression reprogramming^63^.

In this study, we demonstrated that dry seeds are characterized by the enrichment of a specific H2B variant at transcriptionally repressed genes and transposable elements on chromosome arms. The H2B.8 histone variant can form heterotypic nucleosomes, and, due to its structural differences, it is expected to modify nucleosome structure. Upon imbibition, the H2B.8 histone variant is evicted, revealing active chromatin reprogramming that initially occurs in a replication-independent manner accompanying transcriptional reprogramming. Whether histone eviction occurs globally or with distinct dynamics at transposons or genes with specific transcriptional dynamics during the seed-to-seedling transition, is an exciting avenue for future studies.

## Methods

### Plant material

The T-DNA insertion mutants *h2b.8-3* (SM_3_38239), *hira-1* (WiscDsLox362H05) and *ssrp1-2* (SALK_001283C) were obtained from the Nottingham Arabidopsis stock center. Plants were grown under long-day conditions (16 h light, 8 h dark) at 23 °C in Aralab growth chambers. Plants were transformed using floral dipping, and transgenic plants selected on Hygromycin, Kanamycin or Basta according to the resistance marker. After-ripened Arabidopsis seeds were used in all experiments, except for dormancy tests. For the imbibition (48 h in the dark at 6 °C) and germination time-course (long-day conditions, at 23 °C), seeds were spread on water-imbibed filter paper in glass petri dishes. Samples were taken every 24 h (24hD (dark), 48hD, 48hD24hL (light), 48hD48hL and 48hD72hL). Alternatively, seeds were placed on imbibed filter paper in glass petri dishes directly under continuous light. For inhibitor experiments, a hole was introduced into each individual seed with a fine needle (29 G) and seeds incubated in presence of 250 and 500 µM α-amanitin, 100 µM cycloheximide, 50 µM MG132 or a combination of 50 µM MG132 and 50 µM Bortezomib in a small plastic tube under continuous light. For germination tests, seeds were placed on imbibed filter paper in glass dishes. A seed was scored as germinated as soon as the radicle protruded from the seed coat. Ethylene treatment was carried out by placing seeds into 360 mL gas-tight Petri dishes, in which 0.8 mL gaseous ethylene (5%, Air Liquide, Paris, France) was injected with a syringe to obtain a final concentration of 100 ppm of ethylene. For artificial aging, seeds were placed at 37 °C in open tubes situated in a sealed container with 3 M KCL at the bottom. Seeds were stratified for 48 h before placed to long-days for germination. Seed dormancy was released by stratifying at 6 °C for 4 days. Secondary dormancy was induced by placing the seeds for 24 h at 30 °C. Germination rates were then assessed at 15 °C in the dark. To be able to manually dissect embryos from dry seeds, the seeds were imbibed for 30 min in water (for Hi-C, flow cytometry, H2B.8-GFP live cell imaging, GUS staining) or fixed directly by imbibition in 4% paraformaldehyde (nuclear image analysis and immunofluorescence).

### Cloning and construction of transgenic lines

Gibson cloning was used to assemble the different constructs. Each fragment was amplified by PCR and inserted into the pBluescript SK-derived vector pTP1 that contains the *att*L1 and *att*L2 Gateway recombination sites (kind gift from T. Pélissier). These constructs were then transferred into Kanamycin-resistance conferring destination vector pGWB401 (Addgene). All constructs were verified by Sanger or whole-vector Nanopore sequencing. To express H2B.8 or H3.3 histones under the control of the *H2B.8* promoter, 1068 bp of the *H2B.8* promoter region and the 5’UTR as well as the 3’UTR region of the *H2B.8* gene were used. The *H2B.8* coding region was cloned in frame with a 4x Myc-tag (*pH2B.8::Myc-H2B.8*) and the *HTR5* coding region with a 3x Flag-tag (*pH2B.8::Flag-H3.3*). To express Myc-H2B.8 under the *HTR5* promoter, 1016 bp of the *HTR5* promoter region, 5’UTR and its 3’UTR were used. To generate *pH2B.4::Flag-H2B.4* expressing transgenic lines, we used 2318 bp of the *H2B.4* promoter and 5’UTR, the *H2B.4* coding sequence and its 3’UTR to assemble the construct with one Flag-tag by Gibson cloning. For the *pH2B.8::H2B.8-Flag* construct, we synthesized (Genscript) a single construct comprising the *H2B.8* promoter, 5’UTR, CDS without the tail part (corresponding to AA1-150 in translational fusion with a 3x Flag tag and the 3’UTR region of *H2B.8*. The *H2B.8* tail (AA1-150) was then introduced using the Gibson assembly method. To visualize *H2B.8* and *H2B.4* promoter activity during germination, the *GUS* coding regions were cloned under control of 1068 and 2430 bp long fragments of the *H2B.8* or the *H2B.4* promoters respectively in pDonR221 and then transferred in *pBGWFS7* with Gateway cloning kit. To construct the *pH2B.8::H2B.8-GFP* expressing transgenic plants, 2034 bp of the *H2B.8* promoter region and the 5’UTR as well as the coding region of the *H2B.8* gene were inserted into pENTR1A (Thermofisher). The construct was then transferred into the Basta-resistance conferring destination vector pGWB604. To express H2B.4, H2B.8 and the tail of H2B.8 as a fusion protein with the Gal4 DNA binding domain, the coding sequences of *H2B.4* and *H2B.8* as well as the sequence coding for AA1-150 of the H2B.8 tail were cloned as a translational fusion into the pDonR221vector and then transferred into the pGBKT7 destination vector using Gateway cloning. For expression of the same H2B.8 tail fragment (AA1-150) in *E. coli* with a N-terminal GST fusion, the pENT-Tail(H2B.8) was transferred in the pET-GST vector with the same cloning kit. Oligonucleotides used for cloning were obtained from Eurogentec and are listed in Supplementary Data 2.

### X-Gluc histochemical staining

Dissected embryos were fixed for 2 h in 80% acetone at −20 °C, vacuum infiltrated twice 5 min with 0.5 mL of X-Gluc staining solution (50 mM NaPO_4_ pH 7, 20 mM EDTA, 0.2% Triton-X-100, 2 mM Potassium-Ferrocyanide, 2 mM Potassium-Ferricyanure, 2 mM X-Gluc) and incubated 24 h at 37 °C in the dark. Samples were then repeatedly washed in Ethanol at room temperature (RT) and images acquired with an Olympus Stereo SZH microscope under 15x magnification.

### Tetrazolium assay

The tetrazolium assay was performed as described in ref. ^64^ with minor modifications. Pierced seeds were imbibed for 24 h under continuous light in water or in presence of inhibitors, rinsed twice and soaked in a solution of 1% 2,3,5-triphenyl tetrazolium chloride (MP Biomedicals) and then incubated at 29 °C for 24 h. After dissection, embryos were placed in well-plates and imaged under a stereo microscope. Heat killed seeds (100 °C, 1 h) were used as negative control.

### EdU staining

Approximately 30 seeds were imbibed under agitation in water supplemented with 10 µM 5-ethynyl-2’-deoxyuridine (EdU) from the Click-iT® EdU Imaging Kit. The seeds were dissected, and the embryos/seedlings fixed and processed according to the kit’s instructions. Imaging was performed using a spinning disk microscope equipped with a 63x objective.

### Quantification of EdU or H2B.8-GFP positive nuclei

The microscope images (2D for EdU and maximum intensity projections for 3D H2B.8-GFP images) were grouped by replicates using Omero Figure using a double-blind setup. EdU- or H2B-GFP-positive nuclei were counted and the tissue area measured using Fiji before each image was reattributed to the proper sample.

### Flow cytometry analysis

Fifty seeds were harvested at the different time points, and the embryos were dissected and then chopped in 100 µL of cold GB buffer (45 mM MgCl₂, 30 mM Sodium citrate, 20 mM MOPS pH 7, 0.1% Triton X-100, 1% BSA). Samples were then filtered through a 30 µM CellTrics filter and fixed in 2% formaldehyde in GB. To purify the nuclei, the filtrate was deposited on top of an Eppendorf tube containing 100 µL of 2 M sucrose, layered over GSB buffer (0.5 M sucrose, 0.1% BSA, 15 mM Sodium Citrate, 10 mM MOPS pH 7, 0.15% Triton X-100, 0.5% PVP, 0.5% BSA, 22.5 mM MgCl₂). Samples were then centrifuged for 4 min at 4 000 g with minimal braking. The nuclei, localized in the GSB layer, were collected and diluted in GB buffer with 5 µg/mL of DAPI for a final volume of 1 mL and analyzed on an Attune NTX Flow Cytometer. Analyses were performed with FlowJo.

### Nuclear image analysis

Seeds were imbibed in 4% paraformaldehyde in 1X PBS for 2 h before embryos were dissected, stained with Hoechst and imaged as described in ref. ^65^. From the 40 central Z-slices of each image, we extracted small 3D images containing one nucleus each, using the ImageJ plugin *NucleusJ* 2.0^66^. For each genotype, an equal number of nuclei (10-20) per embryo were randomly selected. To segment the nucleus and the intensely stained regions within the nuclei, we generated a deep-learning model using the open-source deep-learning framework *Biom3d*^67^. In short, *Biom3d* was used to train two separate models, one to segment nuclear volumes, and the other to segment the Hoechst-bright regions and to segment nuclei and intensely stained regions in WT and mutant nuclei. All segmented signals were verified by eye in a double-blind mode after overlay with the respective raw image and obviously aberrant predictions discarded. Quantitative parameters were subsequently extracted from the resulting segmentations. The Intensity RHF (ratio between the total intensity of segmented Hoechst-bright chromatin regions and the total intensity of the nucleus) was determined using the “Chromocenter Analysis menu” of *NucleusJ*^66^. Screenshots of representative nuclei and the predicted segmented nuclei and chromocenter masks were obtained using the 3D view in Napari.

### Immunofluorescence staining

Seeds were imbibed for 2 h in 4% paraformaldehyde in 1X PBS, nuclei isolated from dissected embryos and immunofluorescence experiments carried out as described^65^. In brief, embryos were finely chopped with a razor blade in 200 μL LB01 buffer (15 mM Tris-HCl pH 7.5, 2 mM NaEDTA, 0.5 mM spermine, 80 mM KCl, 20 mM NaCl and 0.1% Triton X-100) and filtered through a falcon cell strainer cap (40 µM). After a 1:1 dilution in sorting buffer (100 mM Tris-HCl pH 7.5, 50 mM KCl, 2 mM MgCl_2_, 0.05% Tween-20 and 5% sucrose), 20 µL of the nuclei suspension were spread on a polylysine slide. After drying, the nuclear suspension was fixed in 2% formaldehyde in 1X PBS for 5 min and rinsed. Slides were washed in 1X PBS containing 0.5% Triton X-100 for 15 min at RT, then 3 times with 1X PBS 5 min at RT. For immunodetection, slides were incubated overnight at 4 °C with 20 µL of primary antibody in fresh blocking buffer (3% BSA, 0.05% Tween 20 in 1X PBS), washed 3 times for 5 min each in 1X PBS solution and then incubated for 2–3 h at RT in 20 µL of blocking buffer containing secondary antibodies. Finally, slides were washed 3 times for 5 min each in 1X PBS and mounted in Vectashield mounting medium containing 1.5 µg/mL DAPI (Vector Laboratories). The following antibodies were used: anti-Flag (Abcam, ab18230, batch GR229415-5, 1/100), anti-Myc (Millipore, 05-724, batch 3800004, 1/100), anti-H2A.Z (Agrisera, AS10 718, batch 2410, 1/250) and anti-H3K27me3 (Diagenode, C154110069, batch A1818P, 1/100). The anti-H2A.W antibody was generated against two peptides specific for the H2A.W.6 variant as in ref. ^21^ and used to a final dilution of 1/400 in blocking buffer. Primary antibodies were detected with anti-mouse Alexa-594 (Invitrogen, A21203, 1/1000) and anti-rabbit Alexa-488 (Invitrogen, A11070, 1/1000). Images were acquired with a confocal laser-scanning microscope (LSM800; Carl Zeiss) and with a 63x N.A. 1.4 oil immersion objective. Two laser diodes at 488 nm and 561 nm were used to excite Alexa-488, and Alexa-594, respectively. Images were stored in an OMERO database (https://omero.mesocentre.uca.fr/). Figures were produced using OMERO figure.

### RNA extraction and RT-qPCR

Total RNA was extracted from dry seeds using a combination of Phenol-Chloroform extraction and RNAzol@RT (Molecular Research Center). In brief, 100 mg of seeds were ground in liquid nitrogen using mortar and pestle and taken up in extraction buffer (100 mM Tris-HCl pH 9.5, 150 mM NaCl, 5 mM DTT, 1% Sarkozyl). After vortexing and centrifugation, the supernatant was recovered and mixed with 0.5 Volumes of Chloroform and Acidic Phenol. RNA in the supernatant was precipitated and the pellet taken up in 800 µL RNAzol@RT and treated following the manufacturers’ instructions. DNA was removed with DNaseI (Promega) and finally RNA purified using Zymo RNA purification columns (Zymo Research). For *spike-in* RNA seq, 200 embryos imbibed for 24 h were manually dissected, harvested into ice cold acetone and ground to fine powder using a metal bead in a Qiagen Tissue Lyser. RNA was extracted using the Qiagen micro kit following the manufacturers’ instructions. After adding the first buffer (RLT PLUS), 100 ng of Drosophila total RNA was added to each sample. RNA quality was checked using the Tapestation (Agilent) and quantified by Qubit. For RT-qPCR analysis total RNA was reverse-transcribed using polyT primers using AMV Reverse Transcriptase (Promega) using the manufacturers’ instructions. Transcript levels of histone *H2B* transcripts and H2B.8-target genes were normalized to *MON1 (At2G28390)* and *TIP41 (At4G34270)* using the Roche Lightcycler and qPCR kit (Supplementary Data 2).

### RNA-seq analysis

Library preparation from total RNA and mRNA sequencing was performed by BGI. For dry seeds, raw reads were processed by the bioinformatics pipeline CRESCENT^68^ (https://github.com/gilless429/crescent) developed with the snakemake workflow management system. Quality checking was done using fastqc and multiqc. Low-quality reads were trimmed or removed by cutadapt. Reads were then mapped to the TAIR10 reference genome via the hisat2 mapper, before being assigned to genomic features by featureCounts. Differential expression analysis was executed through the DESeq2 R package and read counts were normalized using DESeq2’s median of ratios method. The thresholds for what was considered a mis-regulated feature were an adjusted p-value (Benjamini-Hochberg procedure) of less than 0.05 and a log2FC of absolute value greater than 1 (Supplementary Data 3). The PCA was produced using DESeq2’s plotPCA, heatmaps using the pheatmap package, after variance stabilizing transformation of read counts and Venn diagrams with the ggvenn package.

For *spike-in* RNA seq analysis of 24 h imbibed seeds, the same snakemake pipeline was used. Reads were mapped to an “hybrid” genome containing the *Arabidopsis thaliana* TAIR10 reference genome and the *Drosophila melanogaster* reference genome DM6 by hisat2^69^ mapper, processed and deduplicated with samtools. Reads were then assigned to Arabidopsis and Drosophila genomic features by featureCounts. *Spike-in* genes (Drosophila) were identified based on their gene identifiers and used exclusively to estimate size factors for normalization. Size factors calculated from *spike-in* counts were applied to Arabidopsis gene counts with the function sizeFactors. Differential expression analysis was executed through the DESeq2 R package (Supplementary Data 4).

### Chromatin-Immunoprecipitation followed by sequencing or qPCR

100 milligrams of seeds expressing the Myc-H2B.8 protein under control of its endogenous promoter were ground to a fine powder in liquid nitrogen cooled mortars. The powder was taken up in Extraction buffer (EB1, 0.4 M sucrose, 10 mM Tris-HCl pH8, 10 mM MgCl_2_, 10 mM β-mercaptoethanol, 0.1 mM PMSF, proteinase inhibitors (Roche)) and the solution filtered through two layers of miracloth. After centrifugation (20 min at 3000 g at 4 °C), the pellet was taken up in EB2a (0.25 M sucrose, 60 mM Hepes, pH8, 10 mM MgCl_2_, 0.3% Triton X-100, 0.1 mM PMSF, proteinase inhibitors (Roche)) and Formaldehyde added to a final concentration of 1%. After 10 min incubation, crosslinking was stopped by adding glycine (140 mM final). Formaldehyde was removed by one wash in EB2a and one in EB2b (0.25 M sucrose, 10 mM Tris-HCl pH8, 10 mM MgCl_2_, 1% Triton X-100, 5 mM β-mercaptoethanol, 0.1 mM PMSF, proteinase inhibitors (Roche)). The nuclear pellet was taken up in 100 µL of Nuclei Lysis (NL) buffer (50 mM Tris-HCl pH8, 10 mM EDTA, 1% SDS, 0.1 mM PMSF, and proteinase inhibitors (Roche)) and left in the fridge for 1.5 hours. After dilution with NL buffer without SDS to 0.1% SDS final, chromatin was sheared using a Covaris sonicator (IP 140, CBP 200, DF 10 %, T° 5 °C, 6 min). Chromatin was stored overnight in the fridge to allow checking sonication efficiency by decrosslinking of a chromatin aliquot overnight and DNA purification using ChIP DNA Clean & Concentrator columns (Zymo Research). Chromatin was incubated with 2 µL of either mouse monoclonal anti-Myc (Millipore, 05-724, batch 3800004) or rabbit polyclonal anti-Myc (Abcam, ab9106, batch 1058571-4) overnight, before adding 30 µL of Dynabeads and incubating for at least 3 hours. Beads with chromatin were washed in low-salt and high-salt buffers as previously described^70^ before elution in 0.1 M NaHCO_3_ and 1% SDS. Chromatin was decrosslinked overnight, treated with Proteinase K and RNase A before purification using ChIP DNA Clean & Concentrator columns (Zymo Research). Several IPs were pooled for each antibody using a ChIP DNA Clean & Concentrator column and DNA concentration determined using Qubit. Library preparation and sequencing was carried out by BGI. For ChIP-qPCR, DNA purified with ChIP DNA Clean & Concentrator columns (Zymo Research) was diluted twice and used in a qPCR reaction with the qPCR Roche Lightcycler and qPCR kit. Primers are listed in Supplementary Data 2.

### ChIP-seq analysis

After read quality check with Fastqc, reads were aligned to the TAIR10 genome using Bowtie2^71^. PCR duplicates were discarded using samtools rmdup^72^. Peaks were called using MACS2^73^ (--qvalue 0.01, --bw 300) using corresponding inputs as background, only peaks overlapping by at least 10% between replicates were retained. Sample tracks were generated using deeptools bamCoverage^74^ with option -normalizeUsing RPGC. Based on H2B.8 peaks’ fold change compared to Input calculated by MACS2 (Supplementary Data 5), H2B.8 peaks were divided in 3 quantiles with high, middle, and low H2B.8 levels to investigate the impact of H2B.8 on chromatin interactions. All genes, for which the gene body overlapped with at least one nucleotide of a H2B.8-peak were considered H2B.8-marked. All other genes were considered H2B.8 free. To compare H3K4me3 and H3K27me3 enrichment at the *H2B.8* gene in seeds and seedlings, data from^30,75^ were normalized using S3norm^76^. All datasets used in this study are listed in Supplementary Data 6.

### Hi-C

For in situ Hi-C experiments, 400 WT and *h2b8-3* mutant seeds per replicate were briefly imbibed before manually dissecting the embryos. Hi-C was performed according to^77^ using the DpnII restriction enzyme (NEB). A second crosslinking step was performed involving disuccinimidyl glutarate and a second digestion with DdeI (NEB) according to Lafontaine et al.^78^. DNA libraries were prepared using NEBNext Ultra II DNA library preparation kit (NEB) according to the manufacturer’s instructions (10 cycles for the PCR amplification step). DNA libraries were checked for quality and quantified using a 2100 Bioanalyzer (Agilent) and the libraries were subjected to 2 × 75 bp high-throughput sequencing by NextSeq 500 (Illumina). Two independent biological replicates were generated yielding over 39 million valid interaction pairs for both control and mutant. The proportion of cis (intrachromosomal) interactions, an indicator of Hi-C data quality, was 85% for the control and 83% for the mutant condition, consistent with the values reported for in situ Hi-C in Arabidopsis confirming the robustness and reliability of our Hi-C datasets.

### Hi-C analysis

Raw Illumina sequencing fastq files were processed with Trimmomatic v0.38^79^. Cleaned reads were mapped independently to the Tair10 reference genome (available at https://www.ncbi.nlm.nih.gov/datasets/genome/GCF_000001735.3/) using HiC-Pro v3.0.0^80^. Bowtie2 parameter settings were default, except “BOWTIE2_GLOBAL_OPTIONS = --very-sensitive-L 30 --score-min L, −0.6, −0.6 --end-to-end –reorder” and “BOWTIE2_LOCAL_OPTIONS = --very-sensitive -L 20 --score-min L, −0.6, −0.6 --end-to-end--reorder” Forward and reverse mapped reads were paired and assigned to DpnII and DdeI restriction fragments. Invalid ligation reads, such as dangling ends, were filtered. Then HiC-Pro v3.0.0 with parameter “-s merge_persample” was performed to merge each pair of replicates before using the valid pairs to generate interaction. After, ‘.hic’ files were generated with the hicpro2juicebox.sh script, which belongs to HiC-Pro then furtherly transformed to ‘.cool’ files and ‘.h5’ files using the function hicConvertFormat from HiCExplorer^81^ at different resolutions. Then, the ‘.h5’ files were normalized using HiCExplorer functions, specifically ‘hicNormalize’ and ‘hicCorrectMatrix’ with the ‘--correctionMethod KR’ parameter. The scaling plot of Interaction frequencies against genomic distance was drawn at a 100 kb resolution from the ‘.h5’ files using ‘hicPlotDistVsCounts.R’^82^. The saddle plots of chromatin compartmentalization and compartment strengths boxplot were drawn at 20 kb resolution by ‘CompareTwoHiCmatrixV2BasedOnHiCdat.R‘^82^. The figures of aggregated Hi-C sub-matrices of different H2B.8 peak quantiles are plotted by coolpup.py^83^ at 25 kb resolution.

### Nuclear protein extraction from seeds and western blot

70 milligrams of dry or imbibed seeds were frozen and ground in liquid nitrogen to a fine powder and homogenized in Honda’s buffer. Nuclei isolation was carried out as previously described^84^ with minor modifications. Nuclear pellets were resuspended in 100 μL of Laemmli 4X buffer, supplemented with β-mercaptoethanol (Bio-Rad), incubated for 5 min at 90 °C and centrifuged at 16 000 g for 5 min. 15-20 μL of nuclear protein extracts were loaded on 4-20% precast SDS-PAGE gels (Bio-Rad) for western blot analysis, using standard procedures. Western blots were probed with different primary antibodies: monoclonal anti-Myc antibody (Millipore, 05-724, batch 3800004, 1/2000), polyclonal anti-Myc antibody (Abcam, AB9106, batch 1058571-4, 1/1000), anti-human H2B antibody (Abcam, ab1790, batch GR3373614-1, 1/2000), anti-Flag antibody (Sigma, F3165, batch SLCJ3741, 1/500) and anti-H3 antibody (Abcam, ab1791, batch GR3237738-1, 1/25000). The anti-H2B.8 antibody (1/1000) was raised in rabbits (Eurogentec) against an AtH2B.8 N-terminal peptide (VSVTKKKKVVEETIK). The antiserum was further purified by peptide affinity column. Primary antibodies were revealed by incubation with a horseradish peroxidase-coupled anti-rabbit (Abliance, BI 2407, batch 241006, 1/5000) or anti-mouse secondary antibody (Abliance, BI 2413 C, batch 231006, 1/5000). Immunoblot chemiluminescence was revealed using ECL protein gel blotting detection reagents (Clarity Western ECL Substrate, Bio-Rad, France). Densitometric analysis of immunoreactive protein bands was performed on non-saturated signals, using Image Lab software (Bio-Rad, France).

### Protein extraction from yeast cultures

Yeast cultures were grown in YNB medium lacking Tryptophan at 29 °C overnight. Cells were pelleted by centrifugation at 2862 g, washed in PBS and taken up in TCA buffer (10 mM Tris HCl pH8, 20% Tricholoroacetic acid, 25 mM NH_4_OAc and 1 mM EDTA). Cells were lysed by vortexing in presence of glass beads and the supernatant recovered. After centrifugation at 16 000 g isolated proteins were taken up in 0.1 M Tris HCl pH11, 3% SDS and supplemented with Laemmli buffer. Gal4 DNA binding domain (DBD)-histone fusion proteins were visualized by western blot using the anti-Myc antibody (Millipore, 05-724, batch 3800004, 1/2000).

### Expression of recombinant H2B.8 tail as GST fusion and protein extraction from bacteria

Rosetta cells (Novagen) transformed with the pET-GST-H2B.8(tail) construct were grown in LB medium supplemented with 100 µg/mL of Ampicillin and 34 µg/mL of Chloramphenicol. Expression was induced by addition of Isopropyl-β-D-Thiogalactopyranoside (IPTG, 0.4 mM). Cells without the construct were used as control. Cells were harvested by centrifugation at 4 500 g for 10 min at 4 °C and then resuspended in 1 mL of 1X PBS supplemented with a Protease inhibitor cocktail (Roche Complete Mini). Lysozyme was added to 0.1 mg/mL and the lysate sonicated on ice (7 ×20 sec ON, 30 sec OFF, amplitude 70%). Triton X-100 was added to a final concentration of 1% and the lysate incubated at 4 °C for 15 min. After centrifugation at 12 000 g for 10 min at 4 °C, the supernatant was collected and analyzed by western blot. The GST-H2B.8(tail) fusion protein was detected using a goat anti-GST antibody (Cytiva 27-4577-01, Sigma Aldrich, batch 322050, dilution 1/4000) or a rabbit polyclonal anti-H2B.8 (described above, dilution 1/5000). Primary antibodies were revealed by incubation respectively with horseradish peroxidase-coupled anti-goat (Abliance, BI 2403, batch 210405, 1/5000) and anti-rabbit secondary antibodies (Abliance, BI 2407, batch 241006, 1/5000).

### MNase accessibility assay

MNase accessibility assays were performed on dry seeds as in ref. ^28^.

### Immunoprecipitation of mononucleosomes

200 milligrams of dry seeds (from WT and from Myc-tagged H2B.8 lines) were ground in liquid nitrogen to a fine powder. The powder was solubilized in 15 mL of extraction buffer (10 mM Tris-HCl pH8, 0.25 M sucrose, 10 mM MgCl_2_, 1% Triton X-100, 5 mM β-mercaptoethanol, supplemented with proteases inhibitors (Roche)), followed by vortexing until a fine suspension was obtained. After incubation for 15 min on ice, the homogenate was filtered through one layer of Miracloth (Merck, Millipore) and nuclei were pelleted by centrifugation at 1400 g for 15 min at 4 °C. Nuclei were resuspended in 10 mL of extraction buffer by pipetting up and down. The nuclei suspension was filtered once again, through one layer of Miracloth and the filtered solution centrifuged at 1400 g for 15 min at 4 °C. The nuclear pellet was washed once in 1 mL of MNase digestion buffer (10 mM Tris-HCl pH7.5, 15 mM NaCl, 60 mM KCl, 1 mM CaCl_2_ supplemented with protease inhibitors). After centrifugation at 1400 g for 15 min at 4 °C, isolated nuclei were resuspended in 250 μL of MNase digestion buffer. Approximately 350 μL of suspension was obtained. An aliquot of 50 μL was kept on ice, as a control of non-digested chromatin. 12 U of MNase (Takara) were added to the nuclei suspension before incubation for 30 min at 37 °C. During the incubation, nuclei were mixed gently every 5 min. At the end of incubation, an aliquot of 50 μL was removed to check digestion efficiency. MNase digestion was stopped on ice by addition of 28 μL of STOP solution (100 mM EDTA, 100 mM EGTA) and nuclei were lysed by addition of NaCl to a final concentration of 500 mM. The suspension was mixed by inverting the tubes and then kept on ice for 15 min. Extracts were cleared by centrifugation for 10 min at 20 000 g at 4 °C. Supernatant (approximatively 300 μL) was diluted by adding 700 μL of MNase buffer. An aliquot of 50 μL was removed for the input. 5 μL of monoclonal anti-Myc antibody (Millipore, 05-724, batch 3800004) diluted in 200 μL of PBS 1X, 0.1 % Tween, was bound on 50 μL of protein G magnetic beads (Dynabeads, Invitrogen) by incubating on a wheel for 30 min at RT. Diluted MNase extracts ( ~ 1 mL) were incubated with antibody-loaded beads over night at 4 °C on a wheel. Beads were washed 3 times with 200 μL of PBS 1X, 0.1 % Tween. For the Flag-IP, the supernatant of the cleared extracts (approximatively 300 μL) was collected and a 50 μL aliquot was removed for the input before dilution. The supernatant was then diluted with MNase buffer to obtain a final NaCl concentration of 150 mM and incubated overnight with anti-Flag M2 magnetic beads (Sigma-Aldrich, M8823, batch SLBm0090V) pre-equilibrated by 2 washes in TBS (50 mM Tris-HCl pH7.4, 150 mM NaCl). After IP, beads were washed 3 times with TBS and supernatant separated from the beads using the magnetic rack. For both IPs, beads were resuspended in 30 μL of Laemmli 4X buffer supplemented with β-mercaptoethanol (Bio-Rad), incubated for 5 min at 90 °C and centrifuged at 16 000 g for 5 min. 5-10 μL of immunoprecipitated proteins were loaded on 4–20% precast SDS-PAGE gels (Bio-Rad) for western blot analysis using standard procedures. To check if mononucleosomes were obtained after MNase digestion, DNA was extracted from aliquots of both nondigested and MNase-digested nuclei. 50 μL of STOP solution (100 mM EDTA, 100 mM EGTA) with 1.5 μL of proteinase K (Invitrogen, 20 mg/mL) were first added to each aliquot before incubation for 30 min at 37 °C. DNA was then extracted with CTAB buffer (2% CTAB, 100 mM Tris-HCl pH8, 1.5 M NaCl, 20 mM EDTA pH8) and isoamylic alcohol/chloroform, before precipitation with isopropanol. DNA was taken up in TE buffer (10 mM Tris-HCl pH7.5, 5 mM EDTA) and treated by RNAse A/T1 (Invitrogen) for 30 min at 37 °C. The DNA concentration was determined using a Nanodrop and equal amounts for each sample were run on a 1.5% agarose gel.

### Statistics and reproducibility

For live imaging experiments, GUS staining, EdU staining and nuclei parameter measurements we carried out at least two independent experiments each comprising multiple embryos. Immunostaining was repeated at least twice with similar results. Western blots and immunoprecipitation experiments were performed at least twice. No statistical method was used to predetermine sample size, and experiments were not randomized. In most experiments, the investigators were not blinded to allocation during experiments and outcome assessment except for quantification of EdU-positive and H2B.8-GFP positive nuclei and during verification of correct image segmentation. No data were excluded from the analyses.

### Reporting summary

Further information on research design is available in the Nature Portfolio Reporting Summary linked to this article.

## Supplementary information

Supplementary Information

Supplementary Data 1

Supplementary Data 2

Supplementary Data 3

Supplementary Data 4

Supplementary Data 5

Supplementary Data 6

Reporting Summary

Transparent Peer Review file

## Source data

Source Data

## Data Availability

The RNA-seq, ChIP-seq et Hi-C data generated in this study have been deposited in the Gene Expression Omnibus database under accession code GSE304463. Image source data as well as images used to train deep learning segmentation models, the corresponding models and extracted image parameters are accessible in an OMERO database (https://omero.mesocentre.uca.fr/) under the accession code 2026_SIMON [10.18145/hj5z-5w89]. The H3K4me3 and H3K27me3 ChIP-seq data on dry seeds used in this study are available in the Gene Expression Omnibus database under accession code GSE243806. The H2A.Z and H3.3 ChIP-seq data on dry seeds used in this study are available in the Gene Expression Omnibus database under accession code GSE209642. The H3K4me3 and H3K27me3 ChIP-seq data on seedlings used in this study are available in the Gene Expression Omnibus database under accession code GSE237185. The RNA-seq data used in this study are available in the Gene Expression Omnibus database under accession code GSE94457 and under accession code GSE94712. The ATAC-seq data used in this study are available in the Gene Expression Omnibus database under accession code GSE250330. The H3K27me3 ChIP-seq data during the seed-to-seedling transition used in this study are available in the Gene Expression Omnibus database under accession code GSE167503. Source data are provided with this paper.

## Code availability

Code from CRESCENT^68^ RNA-seq analysis in dry seeds can be found here: https://github.com/gilless429/crescent.

Code for deep-learning image analysis *Biom3d*^67^ can be found here: https://archive.softwareheritage.org/browse/origin/directory/?origin_url=https://github.com/GuillaumeMougeot/biom3d. Code for the *spike-in* DESeq2 analysis can be found here: https://github.com/laurianesimon/DeSeq2-analysis-spike
