# Supplementary material for: Replication-independent eviction of the histone variant H2B.8 reveals chromatin reprogramming during seed imbibition
Source: Nat Commun. 2026 Jun 29;17:8102. doi: 10.1038/s41467-026-74230-6 (PMC13458839; doi:10.1038/s41467-026-74230-6)
Supplement: Supplementary file 1 [file 41467_2026_74230_MOESM1_ESM.pdf]

Supplementary Information

Replication-independent eviction of the histone variant H2B.8 reveals chromatin reprogramming during seed imbibition

Simon et al.

Supplementary Figure 1

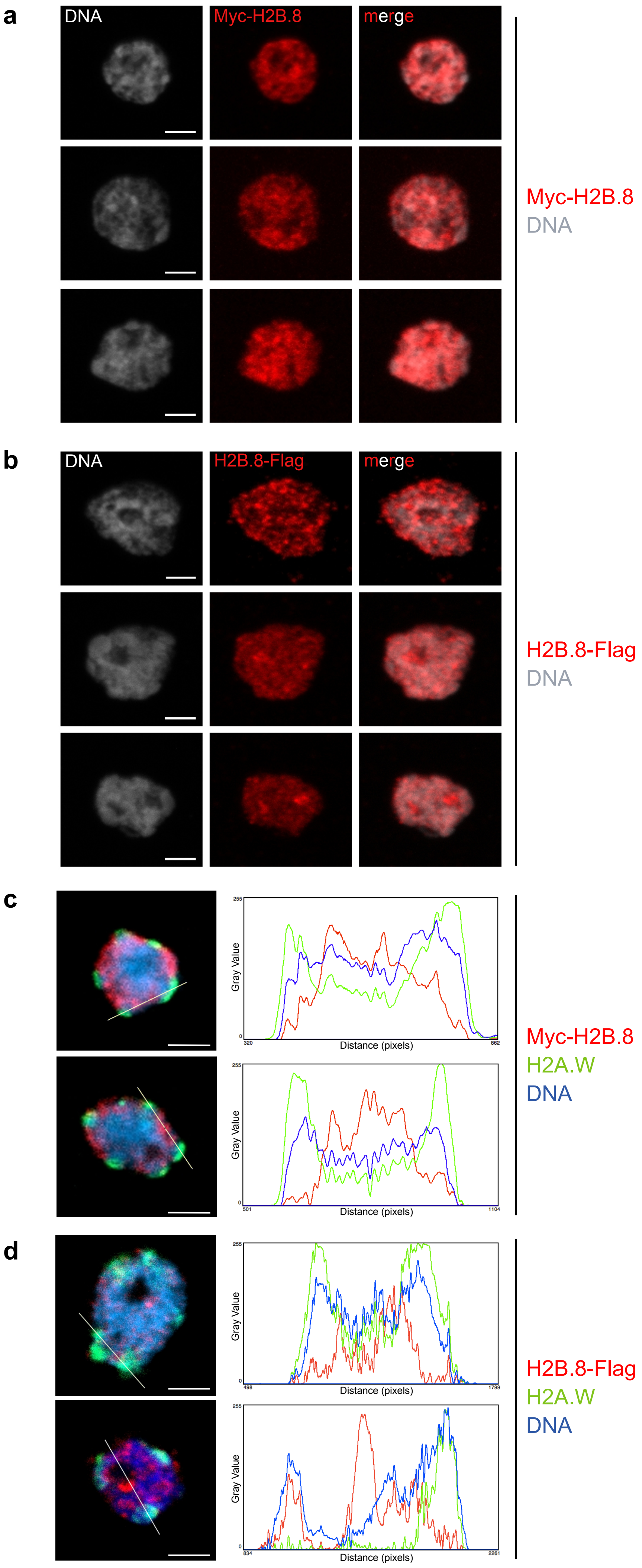

Supplementary Figure 1: H2B.8 is depleted from H2A.W-marked heterochromatin

a-b. Maximum intensity projections of isolated nuclei from dissected embryos from dry seeds (DS) expressing either Myc-H2B.8 (**a**) or H2B.8-Flag (**b**) stained with an anti-Myc or an anti-Flag antibody (red). DNA was counterstained with DAPI (grey). Right panels show merged images. **c-d.** Single Z-slices of DS nuclei expressing Myc-H2B.8 (**c**) or H2B.8-Flag (**d**) stained with anti-H2A.W (green), anti-Myc (red) or anti-Flag (red) antibody and counterstained with DAPI (blue). *ImageJ* was used to draw lines and plot intensity profiles. Most of the chromatin regions that stain intensely with DAPI are marked by H2A.W and therefore correspond to pericentromeric heterochromatin. All scale bars correspond to 2 μm.

Supplementary Figure 2

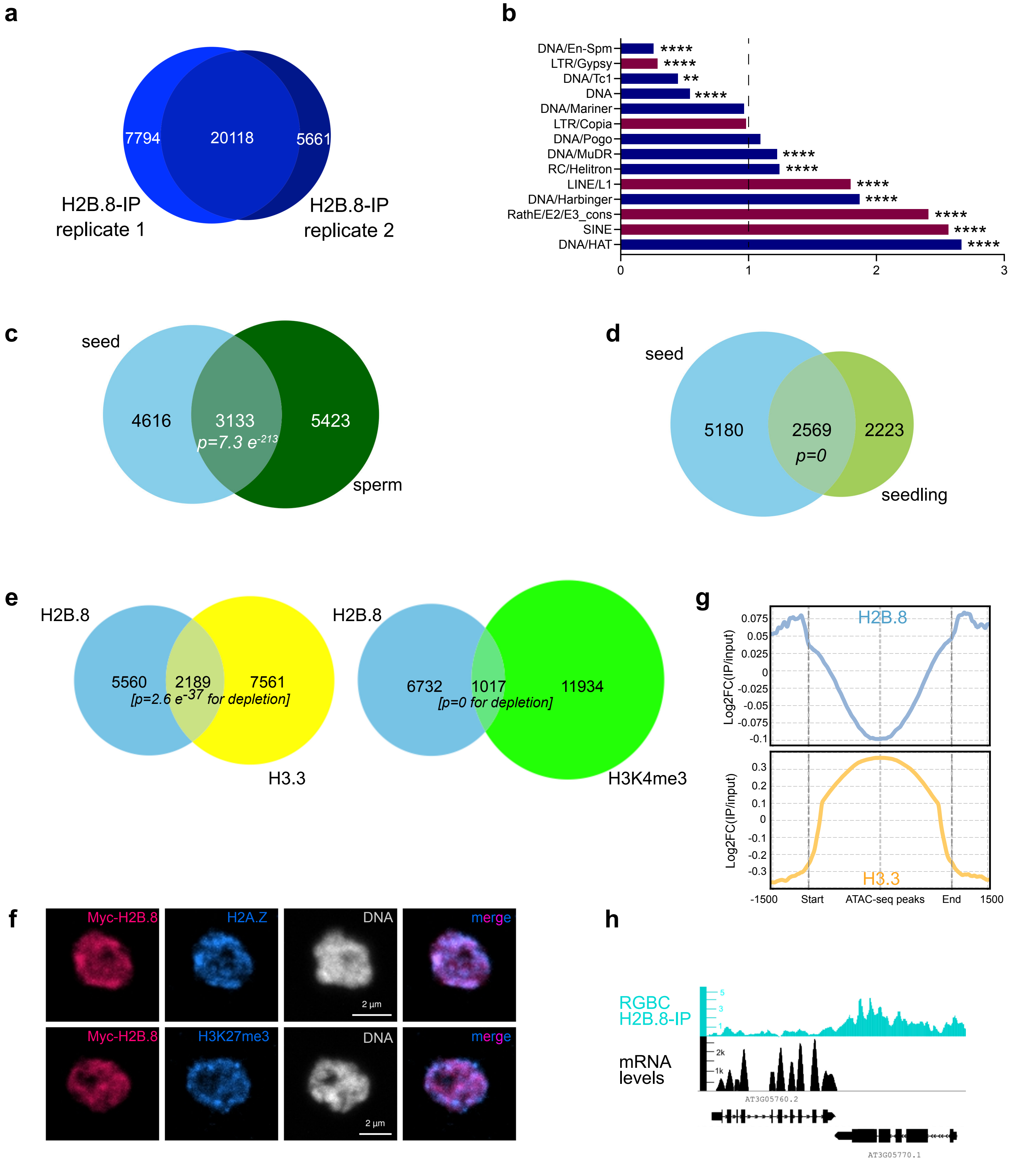

Supplementary Figure 2: Overlap between H2B.8-marked genes in seeds, sperm and ectopically expressing seedlings and depletion of H2B.8 from H3K4me3- or H3.3-marked genes

a. Venn-diagram showing overlap between H2B.8 peaks determined by MACS2 in the two Myc-H2B.8 ChIP-seq replicates from DS. Peaks from the two biological replicates, in which Myc-H2B.8 was either immunoprecipitated with a monoclonal or a polyclonal anti-Myc antibody, show substantial overlap. **b.** H2B.8 occupancy at transposable elements, for which different transposon classes could be assigned (n= 8626 transposable elements). DNA transposons are shown in blue and retrotransposons in violet. Odd Ratio > or < 1, $^{**}p<0.005$ and $^{****}p<0.0001$, Fishers Exact test. **c - d.** Venn diagrams depicting overlap between H2B.8-marked genes in seeds and sperm cells (**c**) or in seeds and seedlings expressing H2B.8 under the 35S promoter (**d**). ChIP-seq data were obtained from [1]. Significant overlap was assessed with a one-sided hypergeometric test. **e.** Venn diagrams depicting overlap between H2B.8-marked genes and genes marked by H3.3 (left) or H3K4me3 (right). H2B.8-marked genes are significantly underrepresented among genes enriched in H3.3 (one-sided hypergeometric test, ChIP-seq data from [2]) or in H3K4me3 (ChIP-seq data from [3]). **f**. Representative DS embryo nuclei stained with DAPI (grey), in which H3K27me3 (blue) or H2A.Z (blue) were revealed by immunostaining together with H2B.8 as an N-terminal Myc fusion (magenta). Scale bar presents 2 µm. **g.** Metagene plot showing H2B.8 and H3.3 enrichment profiles centered on ATAC-seq peaks (from [4]) in DS. **h.** Genome browser view of an H2B.8-depleted and enriched gene in DS together with the corresponding RNA-seq expression profile. Source data are provided as a Source Data file.

Supplementary Figure 3

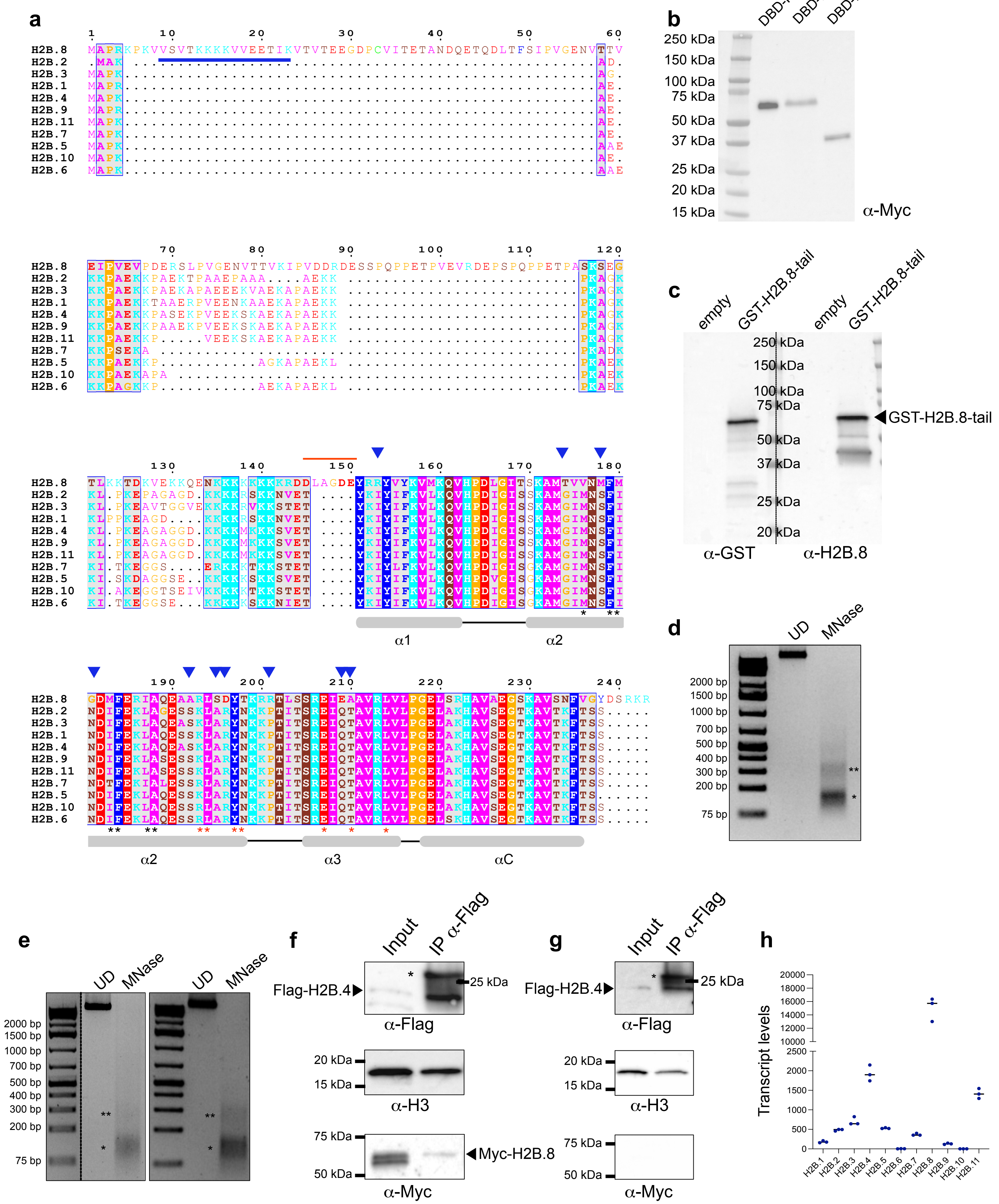

Supplementary Figure 3: Expression and structural features of H2B variants

a. Alignment of the 11 Arabidopsis H2B proteins. Residues are colored as follows: HKR (positively charged), cyan; DE (negatively charged), red; STNQ (polar), maroon; AVLIM (hydrophobic), pink; FYW, blue; PG orange; C, green. Positions of the three α-helices (α1, α2 and α3) constituting the histone fold domain and the C-terminal α-helix (αC) of all H2B histones (except H2B.8) are indicated as grey bars. Critical amino acids in the H2B α2 and α3 helices involved in interactions with H2A α2 or in the H4-H2B four-helix bundle [5] are indicated by black and red stars respectively. Blue arrows indicate amino acid differences in the α2 and α3 helices between H2B.8 and the other H2B variants including one residue involved in the H4-H2B four-helix bundle (210A). The red bar indicates an H2B.8-specific region, comprising two additional acidic amino acids. The blue bar indicates the epitope used to generate the H2B.8-specific antibody. **b.** Western Blot of total yeast proteins expressing H2B.4, H2B.8 and only the H2B.8 tail (AA1-150) as a Gal4 DNA Binding domain (DBD) fusion including a Myc-tag. Molecular weights of 38.3 kDa for Gal4-H2B.4, 49.1 kDa for Gal4-H2B.8, and 38.5 kDa for Gal4-H2B.8-Tail were predicted. **c.** Western Blot of total proteins extracted from control (empty) and *E. coli* cells expressing the H2B.8 tail (AA1-150, predicted molecular weight of 43 kDa) as a GST fusion revealed with anti-GST or anti-H2B.8 antibodies. **d-e.** Agarose gels showing undigested (UD) and MNase digested DNA used in nucleosome immunoprecipitation of Figure 2b (**d**) or Figure S3f and g (**e**), revealing mainly mononucleosomes (*) and small amounts of dinucleosomes (**). **f.** Anti-Flag nucleosome immunoprecipitation from F2 segregating seeds obtained from crosses of lines expressing Flag-H2B.4 or Myc-H2B.8 revealing the presence of H2B.8 in the Flag-H2B.4 containing nucleosomes. Samples derived from the same experiment were run on two different gels and processed in parallel with either the anti-Flag or the anti-H3 and anti-Myc antibody. **g.** Anti-Flag control nucleosome immunoprecipitation from seeds expressing only Flag-H2B.4; * indicates the IgG light chains in **f** and **g**. **h.** Transcript levels (DESeq2 normalization from 3 biological replicates) of *H2B* genes in WT DS.

Supplementary Figure 4

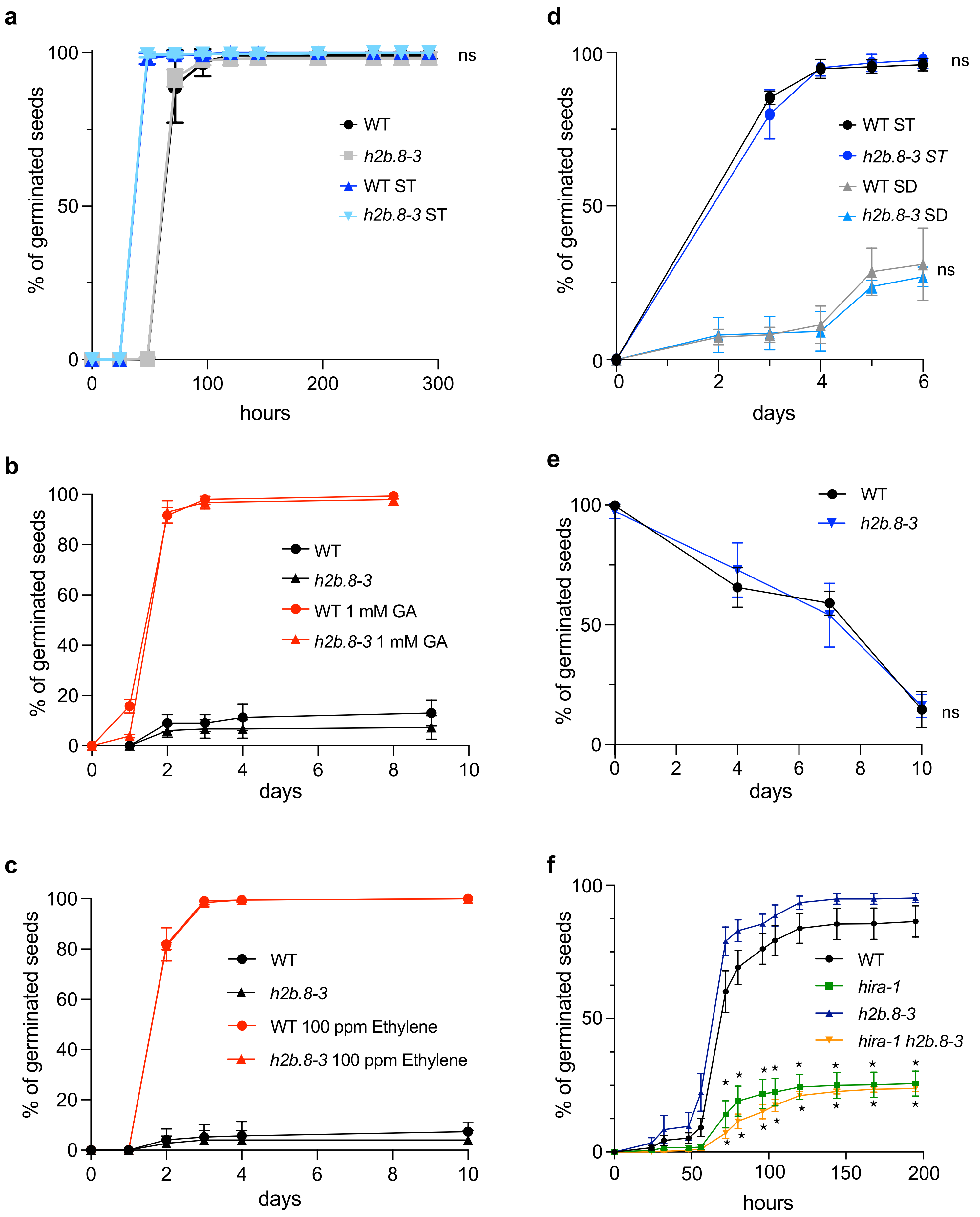

Supplementary Figure 4: Seed germination is unaffected by the absence of H2B.8

a. Seed germination of after-ripened stratified (ST) and non-stratified WT and *h2b.8-3* mutant seeds. Mean of 6 biological replicates for each genotype are shown. Error bars correspond to SD. Statistical differences between WT and *h2b.8-3* mutant seeds were assessed using a 2way Anova with a Tukey's multiple comparisons test; ns, not significant. **b-c.** Germination of after-ripened WT and *h2b.8-3* mutant seeds in the absence or presence of gibberellin (GA, 1 mM, **b**) and Ethylene (100 ppm, **c**). Germination tests were performed at 25°C in the dark. Mean of 3 biological replicates for each genotype are shown. Error bars correspond to SD. Statistical differences between WT and *h2b.8-3* mutant seeds were assessed using a 2way Anova with a Tukey's multiple comparisons test; ns, not significant. **d.** Germination profiles of WT and *h2b.8-3* mutant seeds either stratified (ST) or after induction of secondary dormancy (SD). Statistical differences between WT and *h2b.8-3* mutant seeds under the same conditions were assessed using a 2way Anova with a Tukey's multiple comparisons test; ns, not significant. **e.** Percentage of germinated WT and *h2b.8-3* mutant seeds after 0, 4, 7 or 10 days of artificial aging treatment under high humidity at 37°C. Statistical differences between WT and *h2b.8-3* mutant seeds were assessed using a 2way Anova with a Tukey's multiple comparisons test. ns, not significant. **f.** Germination of freshly harvested and non-stratified WT, *h2b.8-3*, *hira-1* and *hira-1 h2b.8-3* double mutant seeds at 23°C in the dark. Error bars show SEM from 5 or 6 biological replicates. Stars indicate statistically significant differences compared to the WT, $p < 0.005$, 2way ANOVA coupled to a Tukey's multiple comparisons test.

Supplementary Figure 5

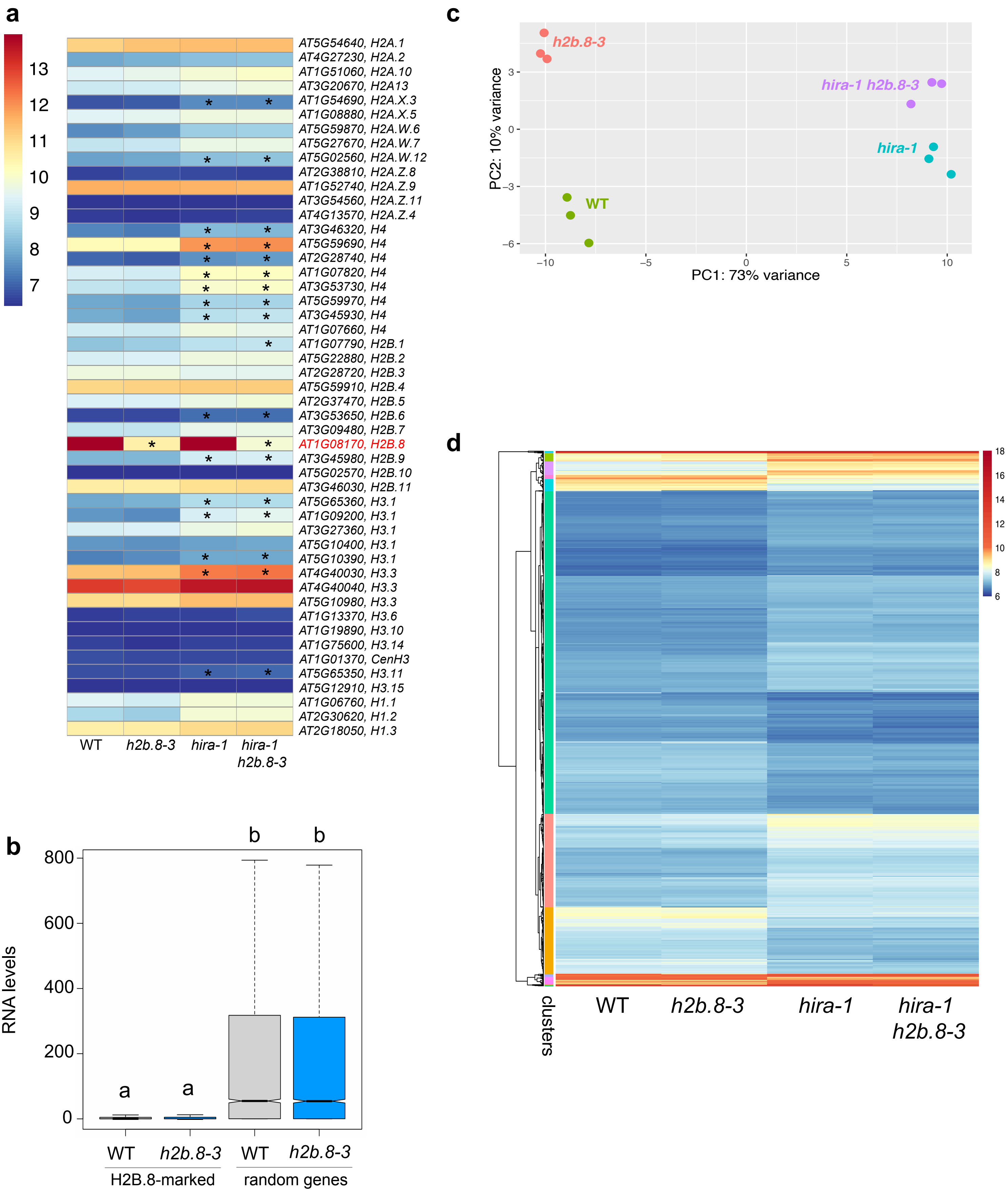

Supplementary Figure 5: Gene expression changes upon depletion of H2B.8 and/or HIRA in dry seeds

a. Heatmap showing normalized read counts of histone gene transcripts in WT, *h2b.8-3*, *hira-1* and *hira-1 h2b8-3* double mutant DS. Stars indicate significantly up- or down-regulated genes (|log2FC| > 1, padj < 0,05) compared to the WT. **b**. Mean transcript levels (DESeq2 normalization) from 3 biological replicates of an equivalent number (n = 7546 genes) of random and H2B.8-marked genes in WT and *h2b.8-3* mutant DS, significant differences indicated by letters were determined with a Kruskal-Wallis test followed by a Dunn's multiple comparison test. The boxplots are centered on the median, the box boundaries represent the second and third quartile, and the whiskers present the 1.5x interquartile range. **c**. Principal component analysis of the three RNA-seq replicates for WT, *h2b.8-3*, *hira-1* and *hira-1 h2b.8-3* double mutants. Single *hira-1* and *hira-1 h2b.8-3* double mutants cluster together. **d**. Heatmap showing normalized read counts for all differentially regulated genes in the dataset. Source data are provided as a Source Data file.

Supplementary Figure 6

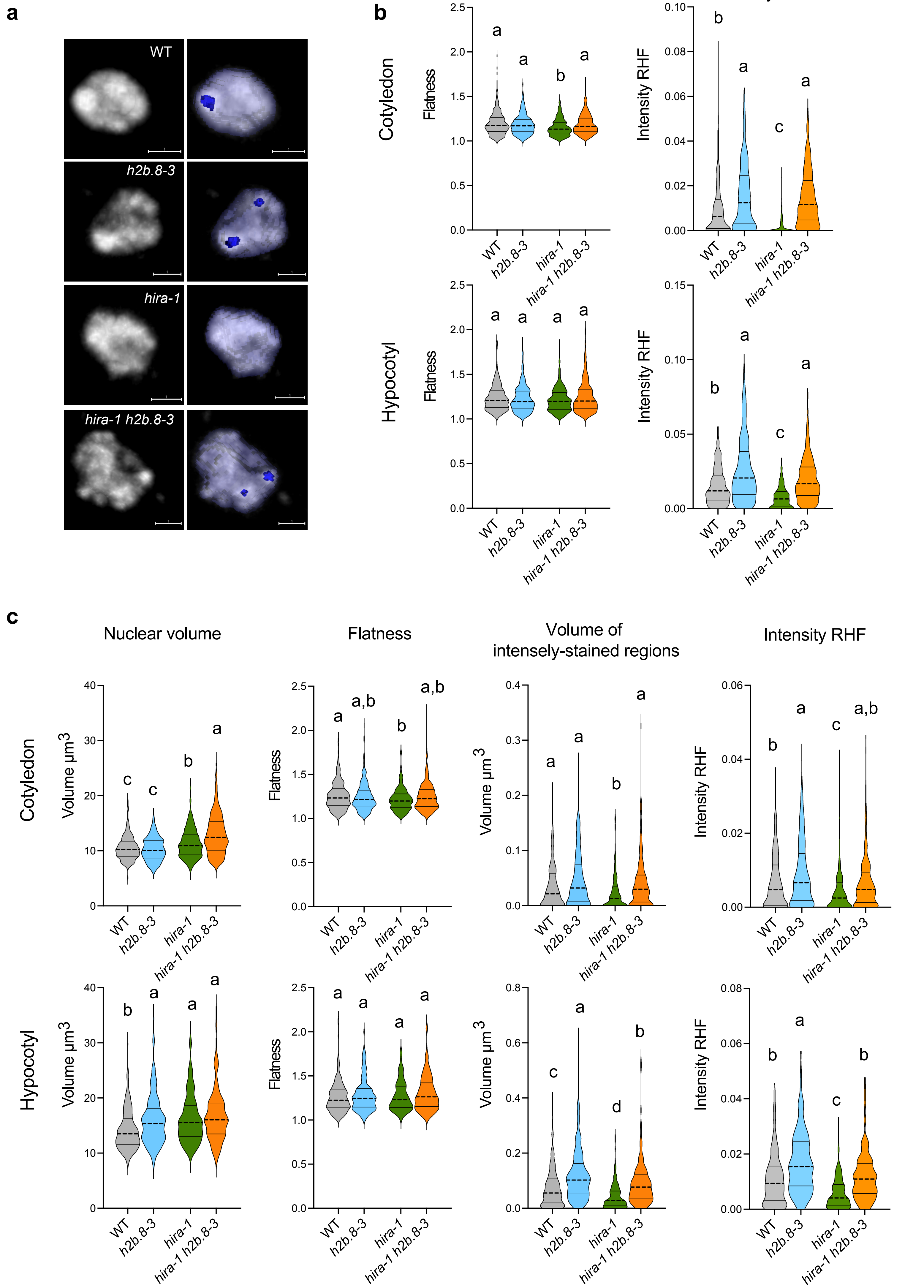

Supplementary Figure 6: Changes in nuclear and chromatin organization upon depletion of H2B.8 and/or HIRA

a. Representative DS cotyledon nuclei from WT, *h2b.8-3*, *hira-1* and *hira-1 h2b.8-3* double mutants, in which the nucleus (light blue) and intensely stained chromatin regions (dark blue) were segmented using a deep-learning model developed in *Biom3d*. Images were generated with Napari. Scale bar presents 2 µm. **b**. Nuclear flatness and Intensity RHF (Relative Heterochromatin Fraction) in cotyledon and hypocotyl nuclei in WT and the three mutant genotypes (*h2b.8-3*, *hira-1* and *hira-1 h2b.8-3*). Nuclei belong to the dataset in Figure 3b and c. **c**. Second independent dataset from an independent seed lot, showing nuclear volume, flatness of the nucleus, total volume of intensely stained chromatin regions per nucleus, and the Intensity RHF. The parameters were determined from cotyledons and hypocotyls from dissected DS using the same deep-learning model generated in *Biom3d* as in Figure 3. Cotyledons: WT: n (number of nuclei) = 233, N (number of embryos) = 24, *h2b.8-3*: n= 273, N =19; *hira-1*: n= 268, N= 19 and *hira-1 h2b.8-3*: n= 271, N= 19. Hypocotyls: WT: n = 229, N = 24, *h2b.8-3*: n= 166, N =19; *hira-1*: n= 174, N= 19 and *hira-1 h2b.8-3*: n= 179, N= 19. Significant differences between genotypes were determined for each parameter using a Kruskal-Wallis test with a Dunn's multiple comparisons test. The exact p-values are given in the source data file. Source data are provided as a Source Data file.

Supplementary Figure 7

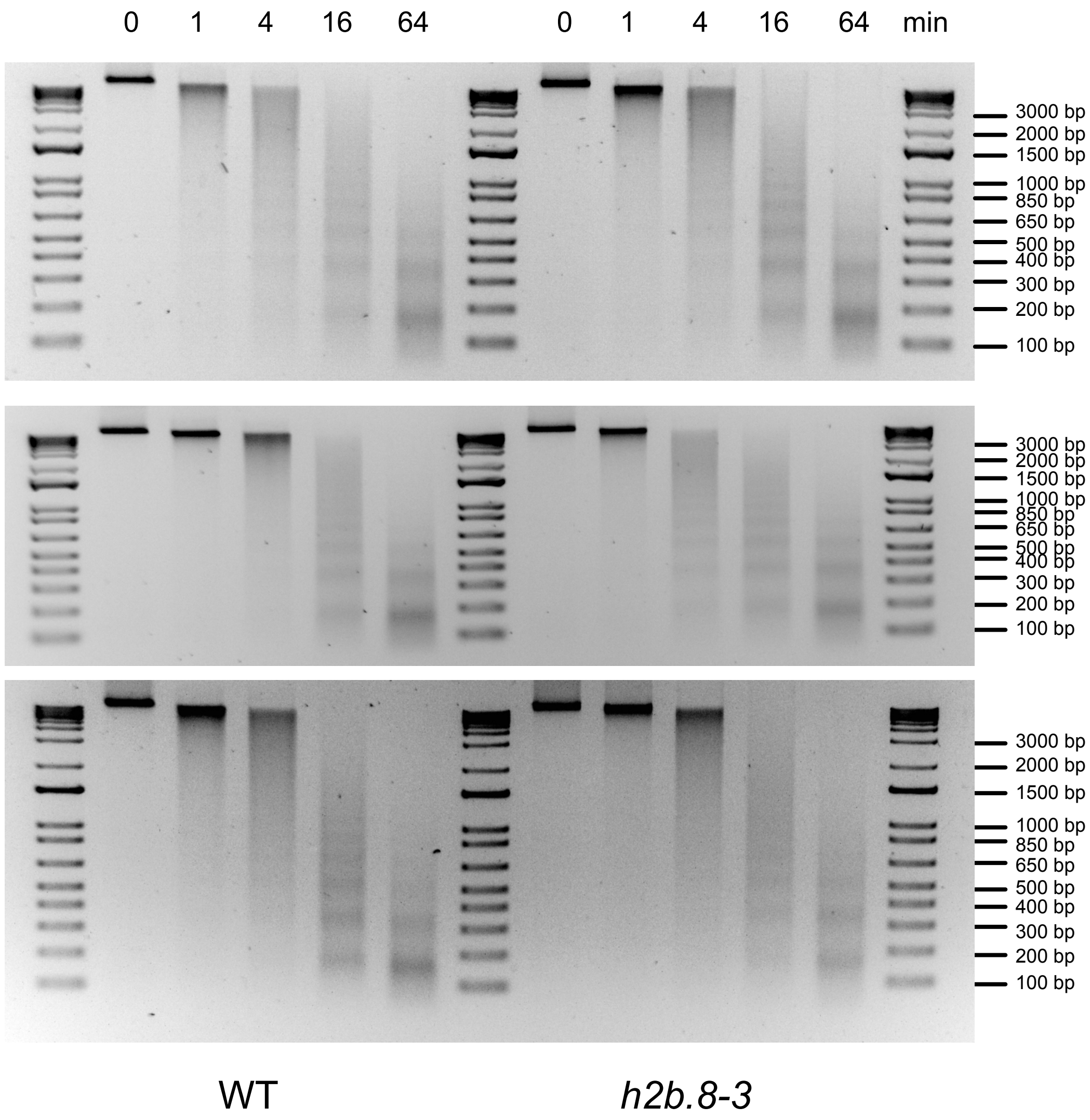

Supplementary Figure 7: MNase digestion followed by agarose gel analysis reveals no major changes in chromatin accessibility in dry seeds upon loss of H2B.8

Chromatin accessibility in WT and *h2b.8-3* mutant DS was assessed by MNase digestion for different durations in three independent biological replicates from different seed batches.

Supplementary Figure 8

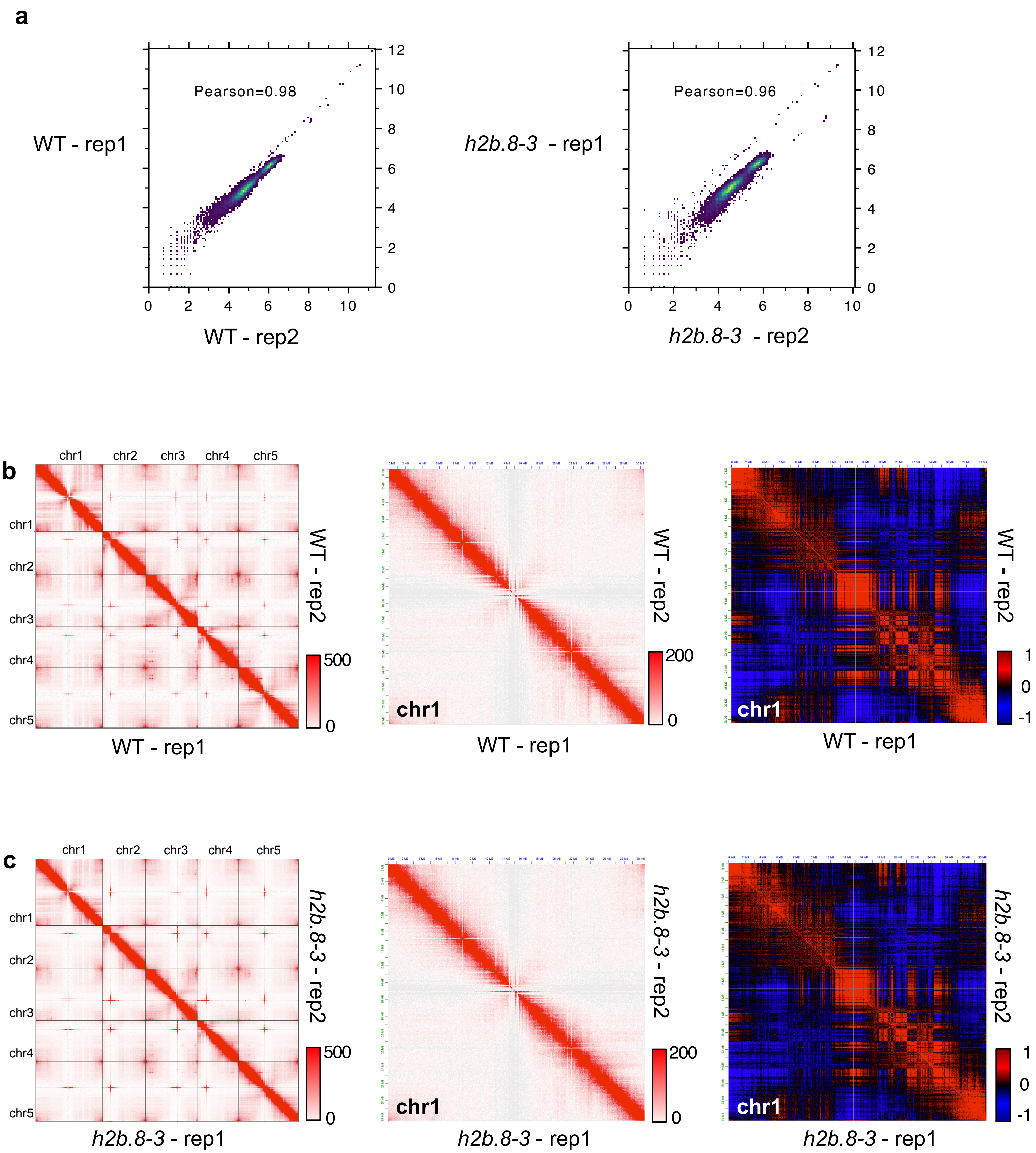

Supplementary Figure 8: Correlation between Hi-C contact matrices

a. Scatter plots showing correlations between Hi-C contact matrices of the two DS WT replicates (left) and the two *h2b.8-3* replicates (right). **b, c**. Hi-C heatmaps of interaction frequencies for the two biological replicates of WT (**b**) and *h2b.8-3* mutant DS (**c**), in all chromosomes (left), and in chromosome 1 (middle), as well as using the Pearson scaling method in chromosome 1 (right) visualized by Juicebox.

Supplementary Figure 9

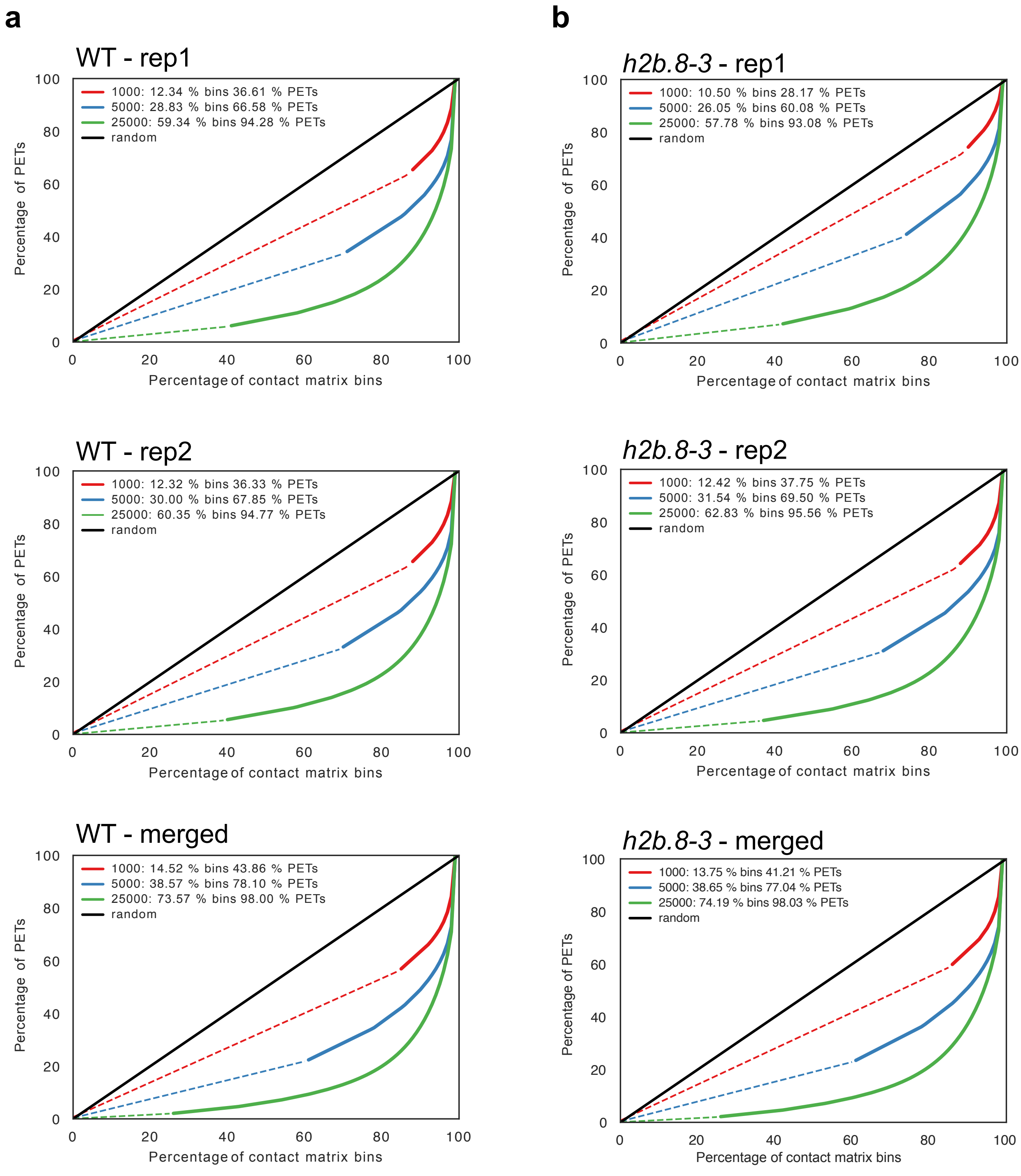

Supplementary Figure 9: Estimation of the contact matrix resolution

a-b. Line plots showing the percentage of paired-end tags (PETs) on the percentage of contact matrix bins for WT (**a**), and the *h2b.8-3* mutant (**b**). The dashed lines indicate the bins from the contact matrix with only singleton PET, and the solid lines indicates the bins with multiple PETs. The random line indicates all PETs are evenly distributed in the contact matrix bins.

Supplementary Figure 10

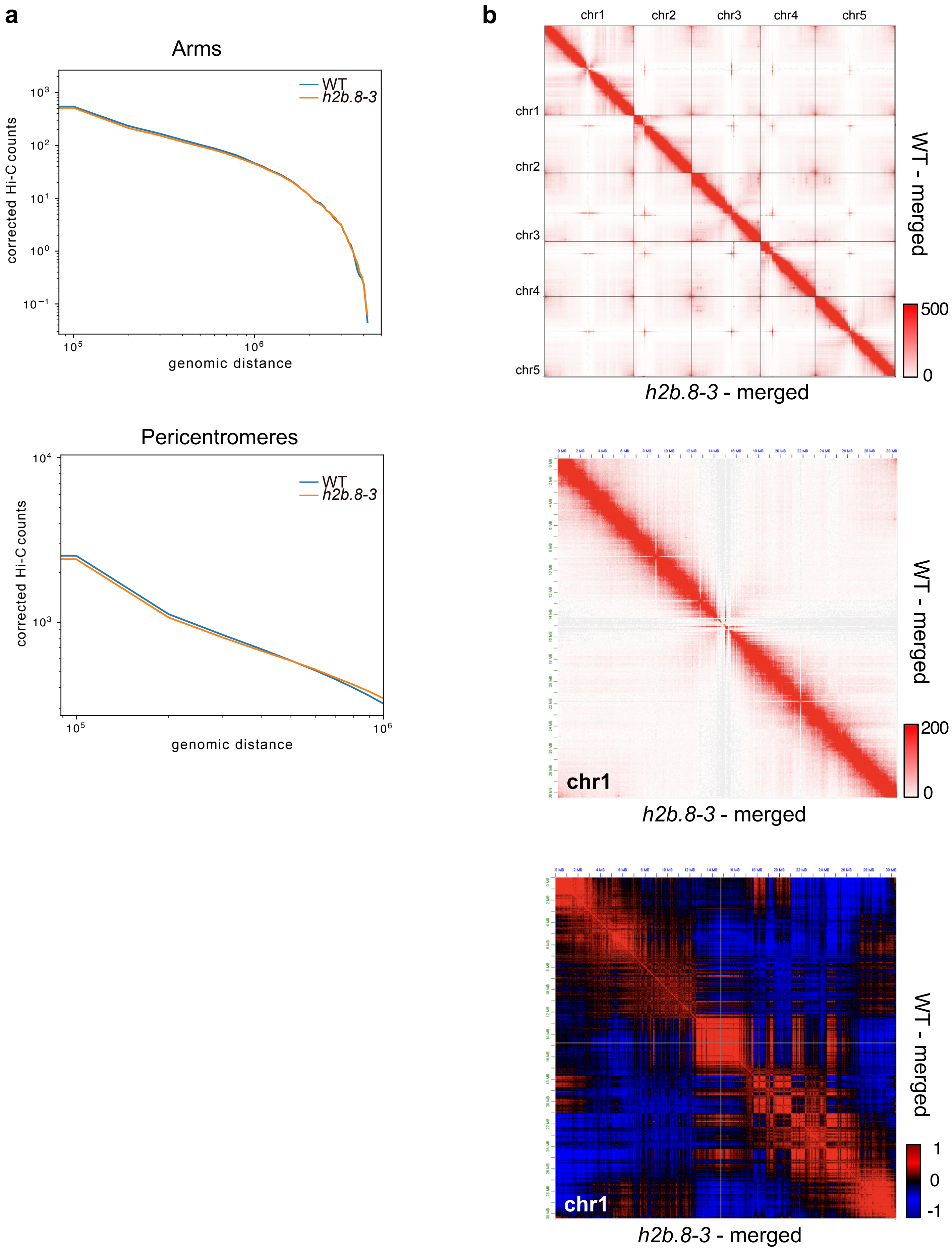

Supplementary Figure 10: Hi-C heatmaps of interaction frequencies

a. Averaged scaling plots with a 100 kb genomic bin size for WT and *h2b.8-3* mutant DS embryos, illustrating how interaction frequencies vary with increasing genomic distance for all chromosomes on chromosome arms (top) and pericentromeric regions (bottom). **b.** Hi-C heatmaps of interaction frequencies for merged WT samples and merged *h2b.8-3* mutant samples, in all chromosomes (top), and in chromosome 1 (middle), as well as using Pearson scaling method in chromosome 1 (bottom) visualized by Juicebox.

Supplementary Figure 11

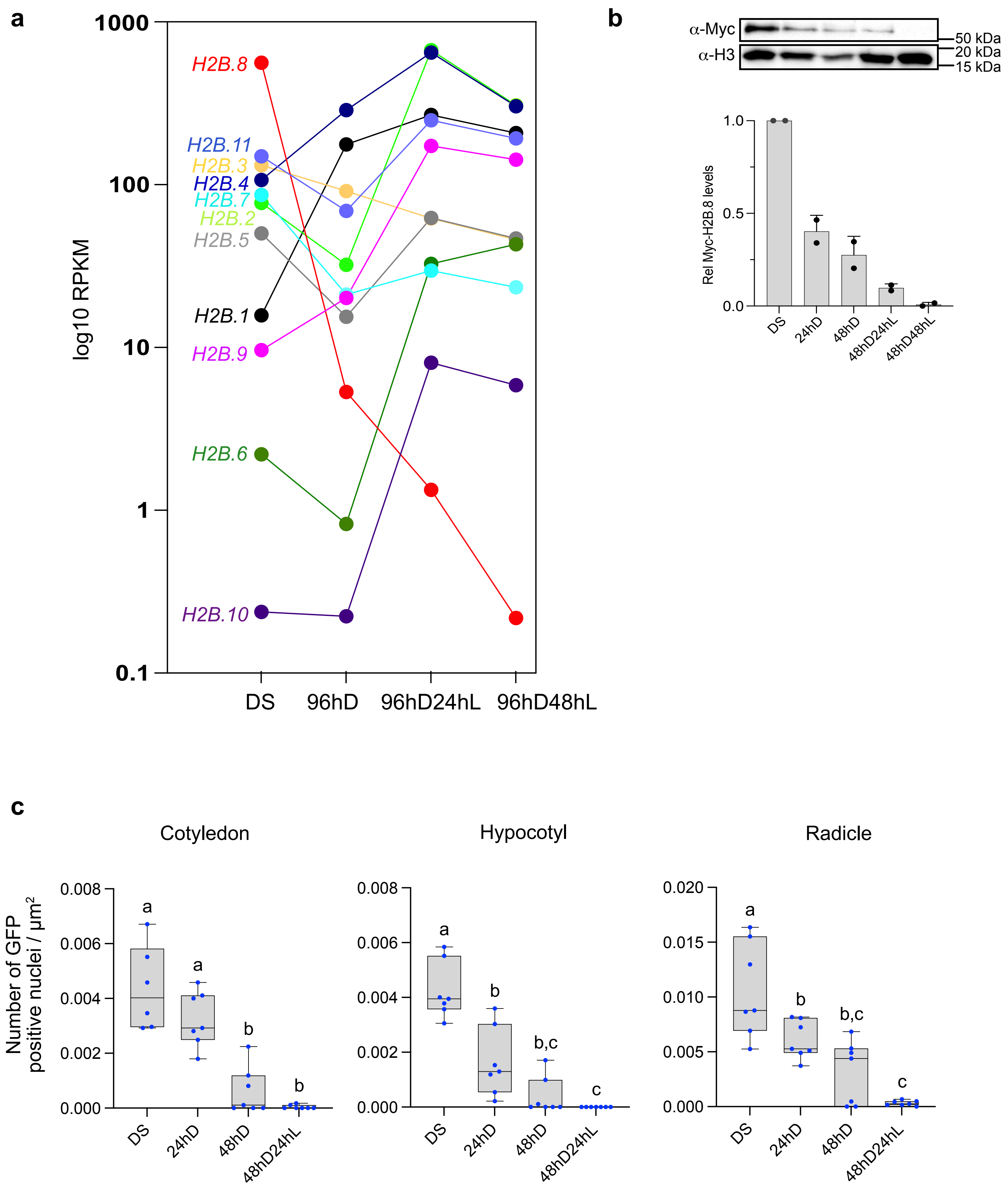

Supplementary Figure 11: H2B.8 eviction during imbibition

a. Expression of the 11 *H2B* genes in DS, after 4 days of stratification in dark and cold (96hD) and 1 or 2 days after light exposure (96hD24hL, 96hD48hL, RNA-seq dataset from [6]). **b**. Representative Western Blot showing Myc-H2B.8 and the H3 loading control (top) and quantification (bottom) of Myc-tagged H2B.8 expressed from the endogenous promoter during the seed-to-seedling transition normalized to H3, n=2 independent experiments, Error Bar presents SD. Normalized Myc-H2B.8 levels in DS were set to 1. **c.** Quantification of H2B.8-GFP positive nuclei per μm^2 tissue in DS, at 24hD, 48hD and 48hD24hL in the cotyledon, the hypocotyl and the radicle. Cotyledon, n≥6; hypocotyl, n=7; radicle, n=7. Letters indicate significant differences between developmental stages determined by an ordinary one-way ANOVA coupled to a Tukey's multiple comparisons test. The boxplot boundaries extend from the 25th to 75th percentiles, the middle line represents the median and the whiskers extend from the smallest to the largest value. Source data are provided as a Source Data file.

Supplementary Figure 12

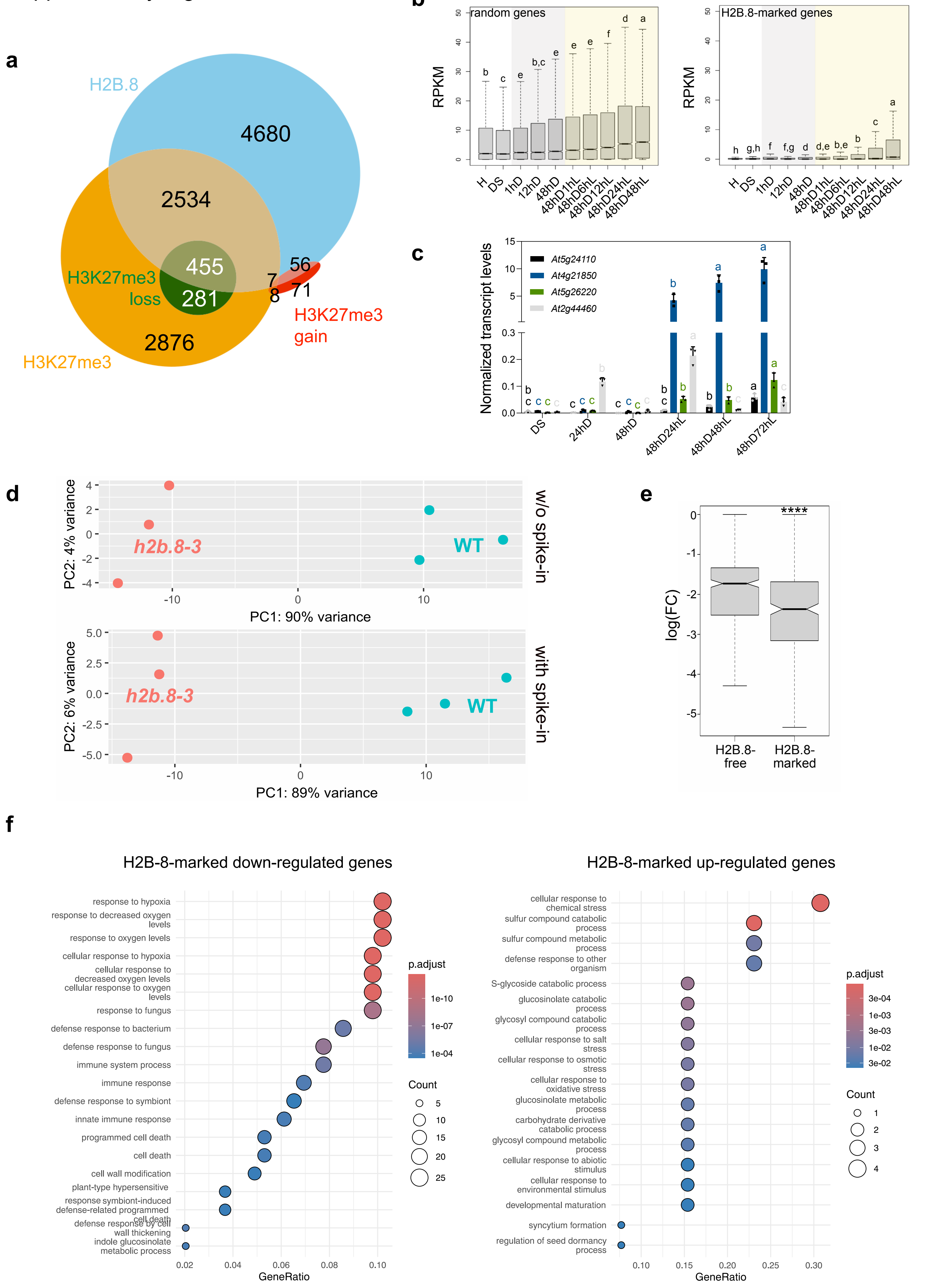

Supplementary Figure 12: Expression of H2B.8-marked genes during the seed-to-seedling transition

a. Venn diagram showing overlap of H2B.8-marked genes and genes that loose or gain H3K27me3 during seed germination (48h in the light; H3K27me3 ChIP-seq datasets were obtained from [7]); *not significant (ns)*, one-sided hypergeometric test. **b**. Differences between mean transcript levels of random (n= 7547 genes) and H2B.8-enriched genes (n= 7547 genes) in RPKM during the seed-to-seedling transition identified in the RNA-seq dataset from [8], which included 3 biological replicates. H = at harvest, DS = dry seeds. The boxplots are centered on the median, the box boundaries represent the second and third quartile, and the whiskers present the 1.5x interquartile range. Comparison of the same developmental stages between the two gene sets reveals significant differences at each time point, **** $p<0.0001$, Kruskal-Wallis test coupled to a Dunn's multiple comparisons test (not indicated in the figure) except for the two 48hD48hDL time points, which are not significantly different. Letters indicate significant differences between time points within a set of genes, Kruskal-Wallis test coupled to a Dunn's multiple comparisons test. **c**. Validation of the expression dynamics of four H2B.8-target genes by RT-qPCR during the seed-to-seedling transition in our experimental setup. Letters indicate significant differences between time points for the gene in the respective color, one-way ANOVA with Tukey's multiple comparisons test, n=3 biological replicates, error bars represent SD. **d**. Principal component analysis for gene expression patterns in WT and *h2b.8-3* mutants at 24hD with and without normalization to Drosophila *spike-in* RNA. **e.** Comparison of log2(FC) changes between H2B.8-free (n=399) and H2B.8-marked (n=288) genes, downregulated in *h2b.8-3* mutants. **** <0.001, Mann-Whitney test. The boxplots are centered on the median, the box boundaries encompass the second and third quartile, and the whiskers present the 1.5x interquartile range. **f.** GO term analysis of the H2B.8-marked downregulated (n=288) and upregulated (n=19) genes. Significantly enriched GO terms were determined with the R package clusterProfiler. Source data are provided as a Source Data file.

Supplementary Figure 13

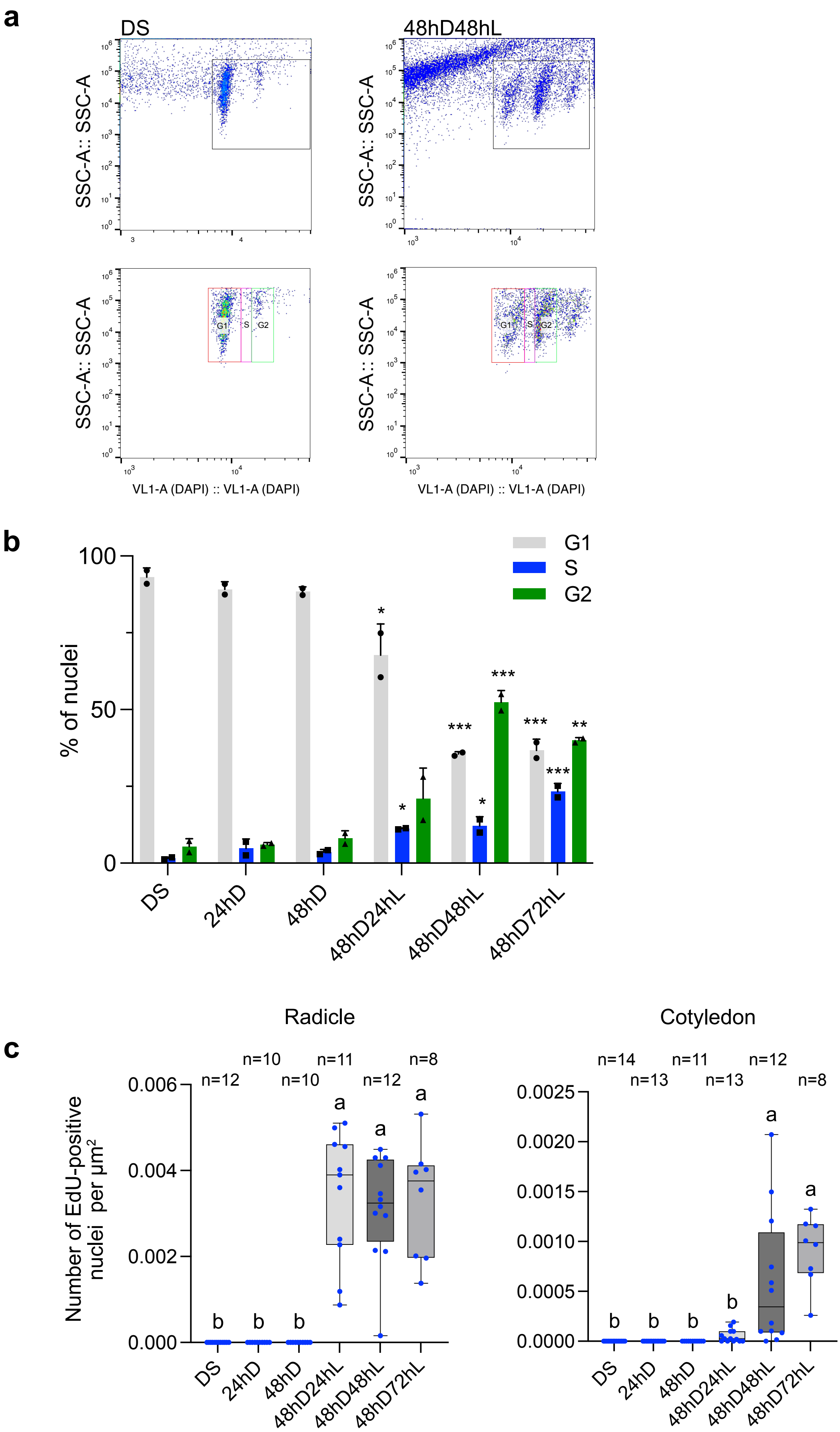

	DS	24hD	48hD	48hD24hL	48hD48hL	48hD72hL
% of EdU-positive shoot apical meristems	0	0	0	0	75	55,6
n	12	11	9	11	12	9

Supplementary Figure 13: Timing of replication onset during the seed-to-seedling transition

a. Representative examples of gate settings for G1, S and G2 nuclei isolated from DS or 48hD48hL germinating seeds. **b**. Quantification of G1, S and G2 nuclei during the seed-to-seedling transition. Two biological replicates have been performed. Statistical differences compared to the G1, S or G2 values obtained DS are indicated. * $p < 0.05$, ** $p < 0.01$, *** $p < 0.001$, One-way ANOVA coupled to a Tukey's multiple comparisons test. Error bars correspond to SD. **c.** Quantification of EdU-positive nuclei per µm^2 tissue in DS and during the seed-to-seedling transition in the radicle and the cotyledon. The boxplot boundaries extend from the 25th to 75th percentiles, the middle line represents the median and the whiskers extend from the smallest to the largest value. The number of independent embryos assessed are indicated. Letters indicate significant differences between developmental stages determined by an ordinary one-way ANOVA coupled to a Tukey's multiple comparisons test. The table indicates the percentage of EdU-positive shoot apical meristems at each developmental stage.

Supplementary Figure 14

a

replicate 1	40.0%
replicate 2	25.5%
replicate 3	57.7%

b

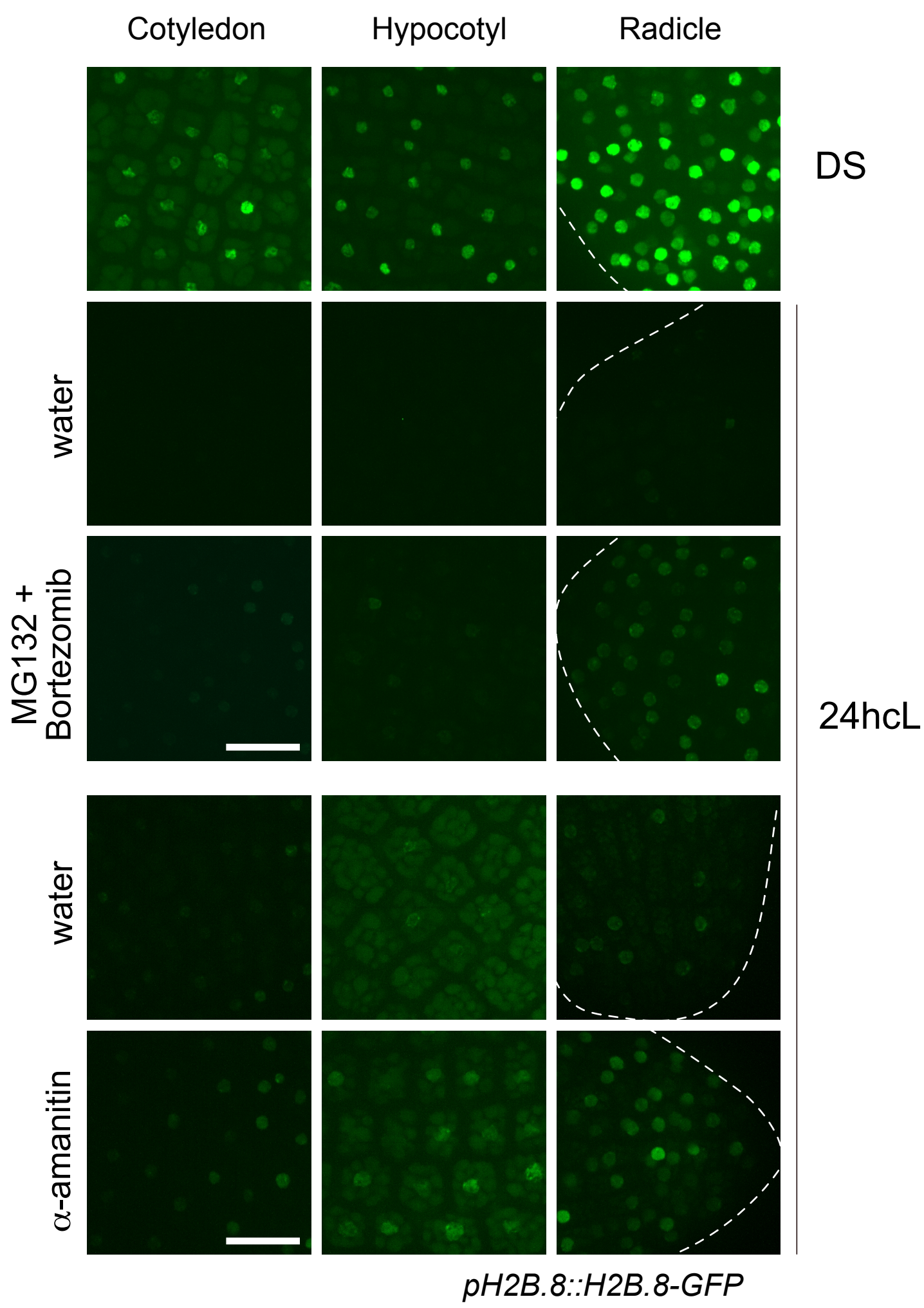

c

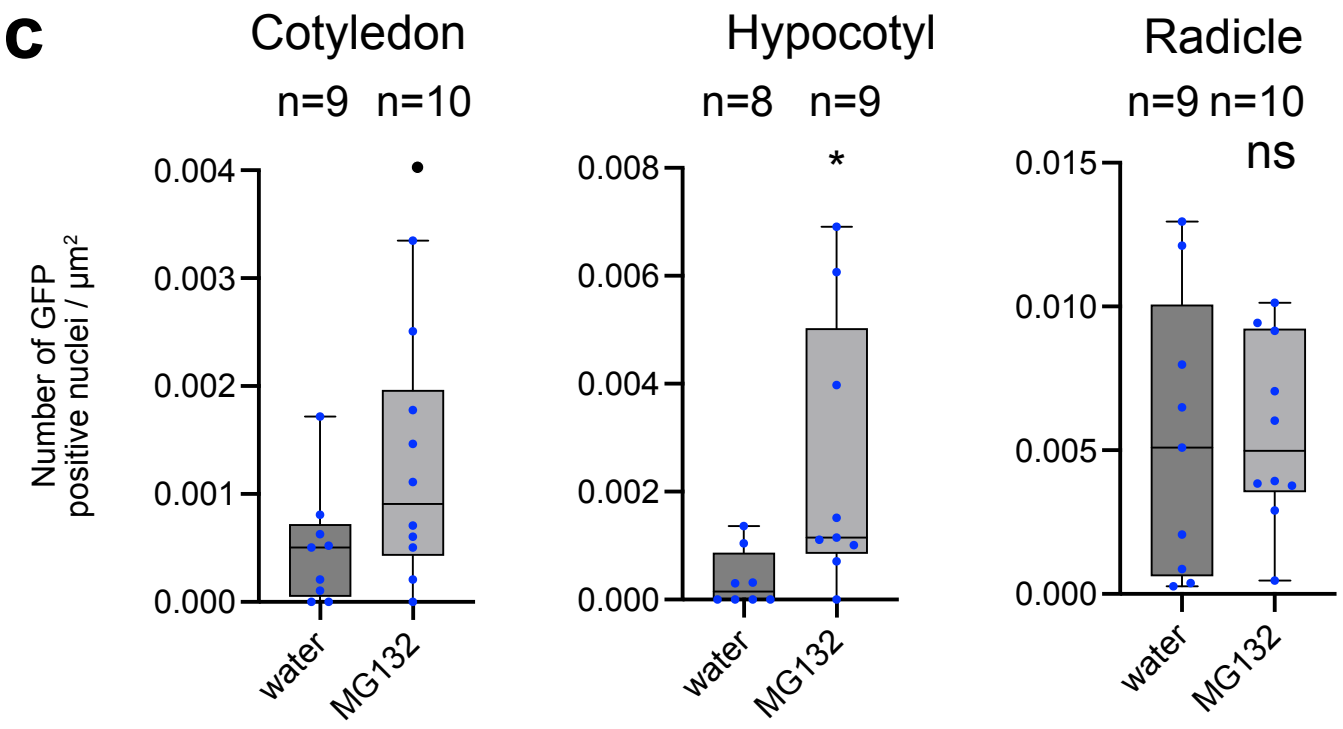

d

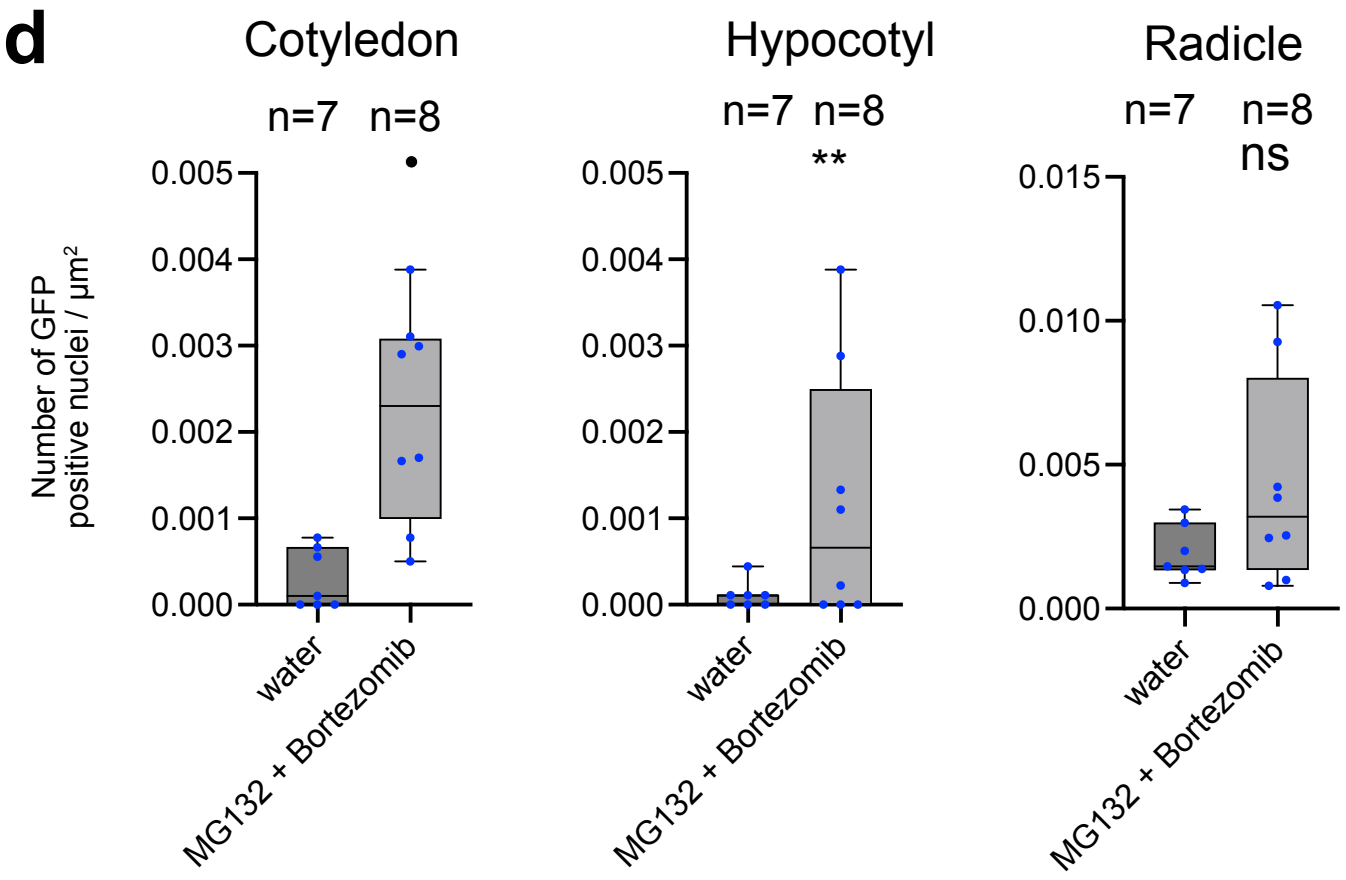

e

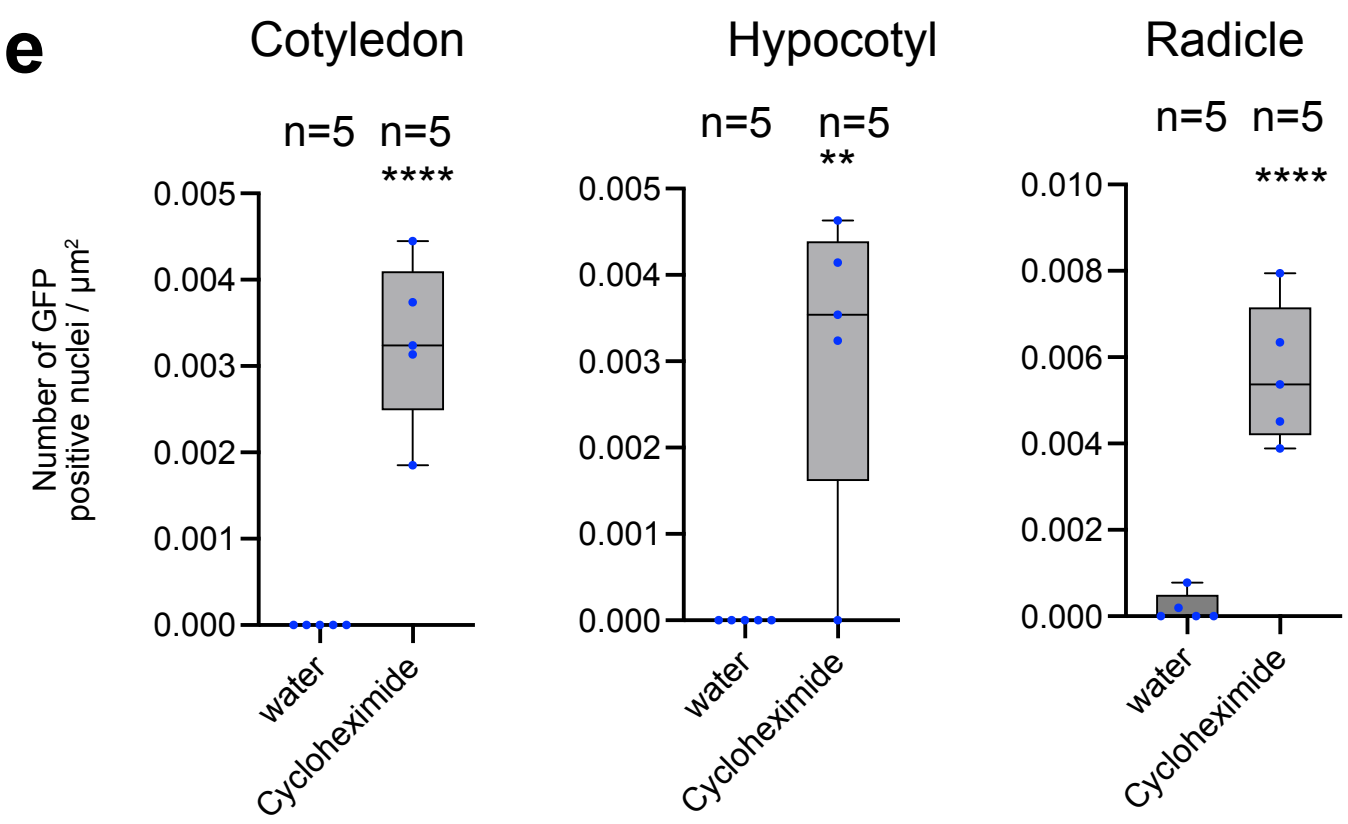

f

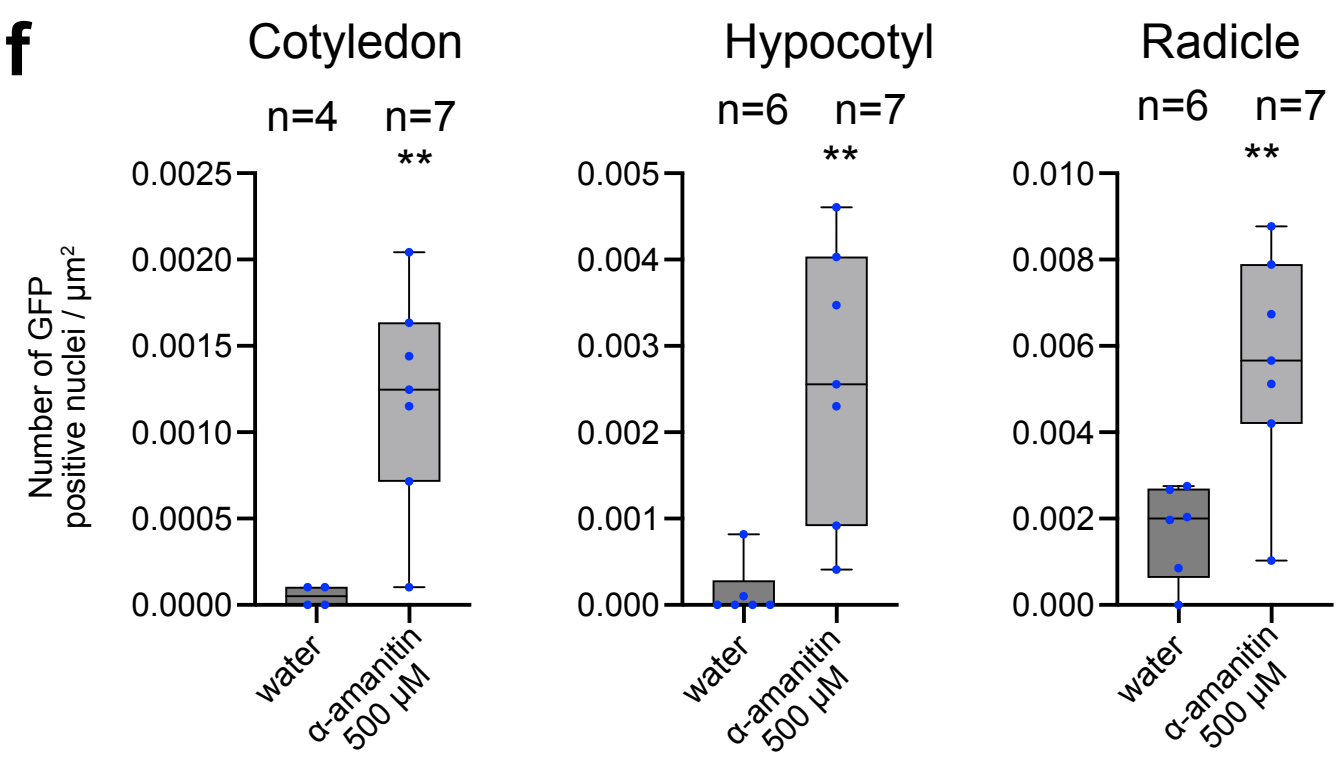

g

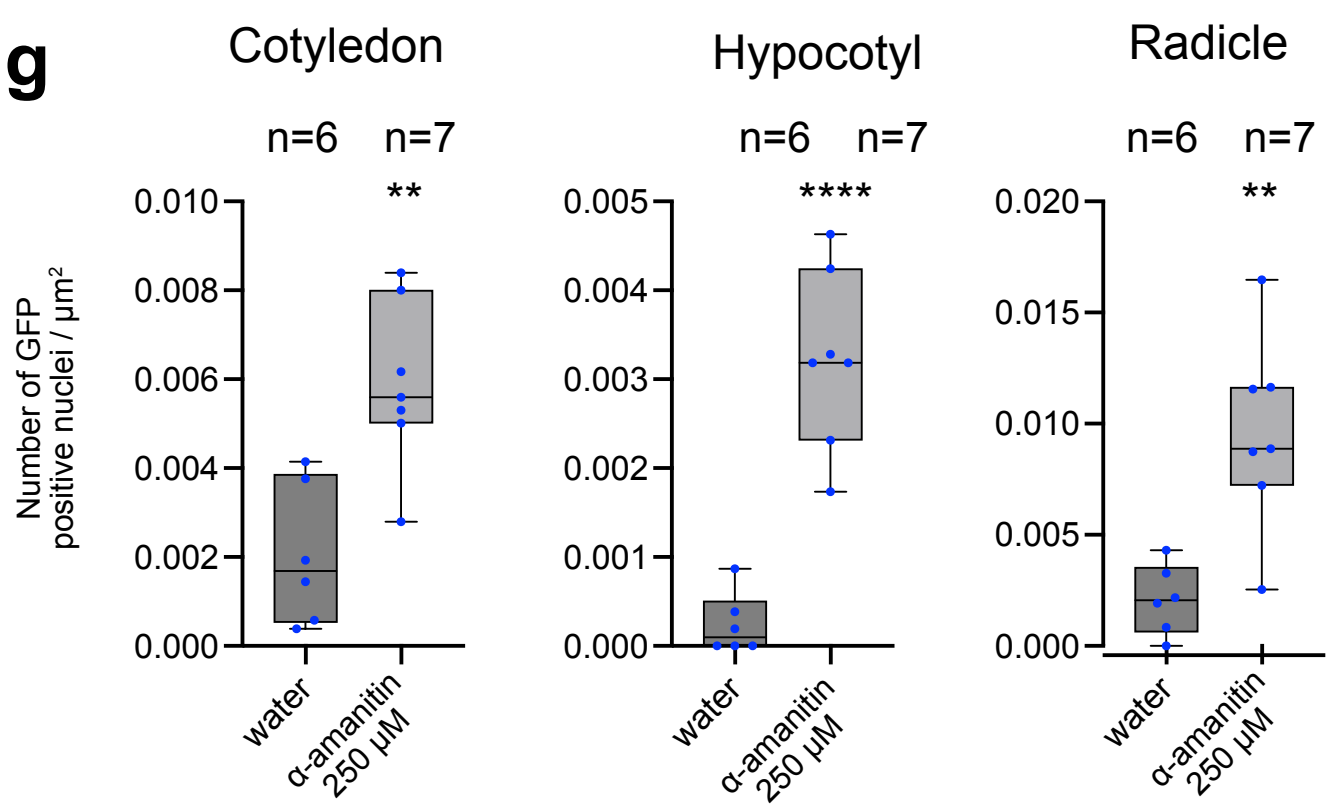

h

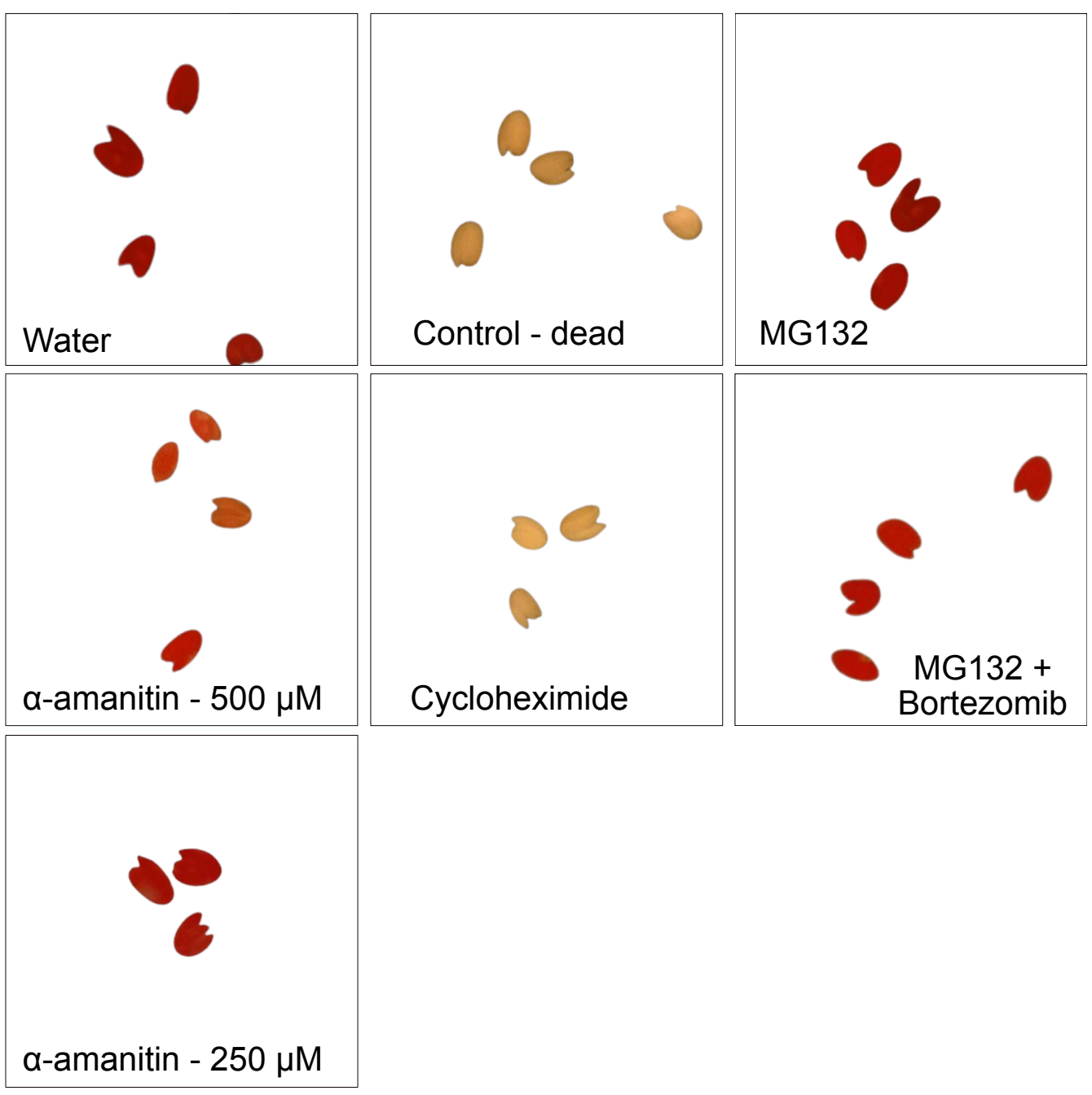

Supplementary Figure 14: Eviction of H2B.8 during the seed-to-seedling transition requires transcription and proteasome activity

a. Percentage of germinated seeds at 48h under continuous light (cL). **b**. Representative maximum intensity projections of cotyledons, hypocotyls and radicles from embryos expressing H2B.8 as a GFP fusion protein under its endogenous promoter isolated from DS or imbibed seeds under continuous light (24hcL). Images were taken with a 63x objective on a Spinning confocal microscope, using identical exposure settings for treated and untreated samples. Seeds were imbibed in water or in the presence of a combination of MG132 and Bortezomib (50 µM each) or α-amanitin (250 µM). DS are shown for comparison. Scale bar represents 20 µm. **c-g.** Quantification of H2B.8-GFP positive nuclei per µm^2 tissue in dissected embryos imbibed at 23°C under continuous light in the cotyledon, the hypocotyl and the radicle. The boxplot boundaries extend from the 25th to 75th percentiles, the middle line represents the median and the whiskers extend from the smallest to the largest value. Significant differences were determined using an unpaired ttest, • $p < 0.1$, * $p<0.05$, ** $p<0.01$, **** $p<0{,}0001$. The exact p values for each test can be found in the Source Data file. The number of embryos analyzed, and the inhibitor treatment are indicated. **h.** Dissected embryos from seeds treated with different inhibitors and subsequently incubated in the presence of 2,3,5-triphenyl tetrazolium chloride (TTC) to reveal active metabolism. Seeds killed by heating to 100 °C for 1h were used as negative control.

Supplementary Figure 15

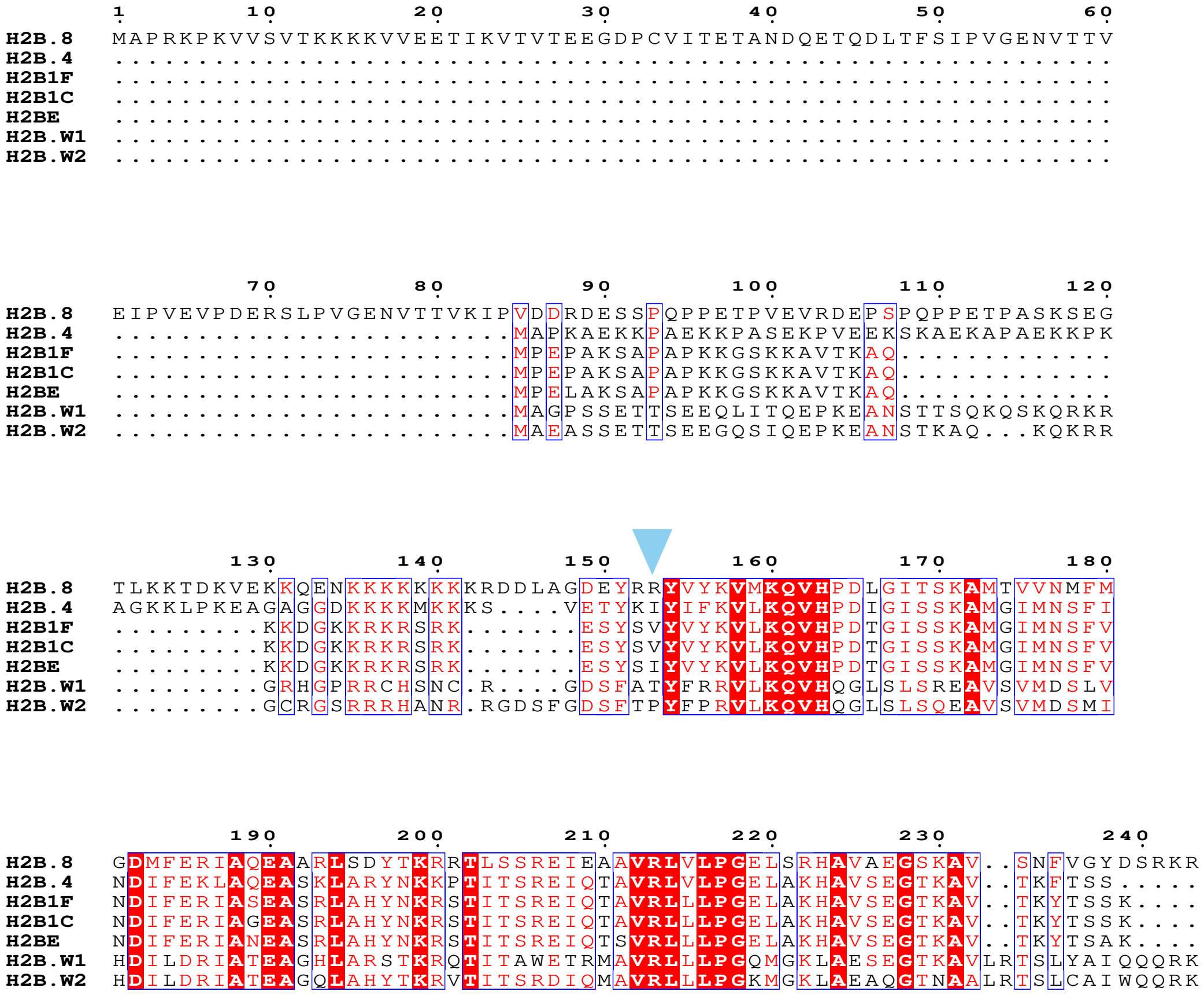

Supplementary Figure 15: Comparison of atypical H2B variants

Alignment of H2B variants from Arabidopsis (H2B.8, H2B.4), mouse (H2B1F, H2BE) and human (H2B1C, H2B.W1 and H2B.W2) generated with MUSCLE and ESPript 3.0. The blue arrowhead indicates the residue identified to alter chromatin accessibility in nucleosomes containing mouse H2BE [9].

Supplementary References

1. Buttress, T. *et al.* Histone H2B.8 compacts flowering plant sperm through chromatin phase separation. *Nature* **611**, 614–622 (2022).
2. Zhao, T. *et al.* Histone H3.3 deposition in seed is essential for the post-embryonic developmental competence in Arabidopsis. *Nat. Commun.* **13**, (2022).
3. Zhang, Q. *et al.* aChIP is an efficient and sensitive ChIP-seq technique for economically important plant organs. *Nat. Plants* **9**, 1317–1329 (2024).
4. Tremblay, B. J. M. *et al.* Interplay between coding and non-coding regulation drives the Arabidopsis seed-to-seedling transition. *Nat. Commun.* **15**, (2024).
5. Luger, K., Mäder, A. W., Richmond, R. K., Sargent, D. F. & Richmond, T. J. Crystal structure of the nucleosome core particle at 2.8 A ˚ resolution. *Nature* **389**, 6648 (1997).
6. Kawakatsu, T., Nery, J. R., Castanon, R. & Ecker, J. R. Dynamic DNA methylation reconfiguration during seed development and germination. *Genome Biol.* **18**, (2017).
7. Pan, J., Zhang, H., Zhan, Z., Zhao, T. & Jiang, D. A REF6-dependent H3K27me3-depleted state facilitates gene activation during germination in Arabidopsis. *Journal of Genetics and Genomics* **50**, 178–191 (2023).
8. Narsai, R. *et al.* Extensive transcriptomic and epigenomic remodelling occurs during Arabidopsis thaliana germination. *Genome Biol.* **18**, (2017).
9. Feierman, E. R. *et al.* Histone variant H2BE enhances chromatin accessibility in neurons to promote synaptic gene expression and long-term memory. *Mol. Cell* **85**, 2822–2837 (2024).
