# Supplementary material for: Replication-independent eviction of the histone variant H2B.8 reveals chromatin reprogramming during seed imbibition
Source: Nat Commun. 2026 Jun 29;17:8102. doi: 10.1038/s41467-026-74230-6 (PMC13458839; doi:10.1038/s41467-026-74230-6)
Supplement: Supplementary file 2 [file 41467_2026_74230_MOESM8_ESM.pdf]

nature portfolio

Corresponding author(s): Aline Probst

Last updated by author(s): Mar 4, 2026

Reporting Summary

Nature Portfolio wishes to improve the reproducibility of the work that we publish. This form provides structure for consistency and transparency in reporting. For further information on Nature Portfolio policies, see our Editorial Policies and the Editorial Policy Checklist.

Statistics

For all statistical analyses, confirm that the following items are present in the figure legend, table legend, main text, or Methods section.

n/a	Confirmed	
☐	☒	The exact sample size (*n*) for each experimental group/condition, given as a discrete number and unit of measurement
☐	☒	A statement on whether measurements were taken from distinct samples or whether the same sample was measured repeatedly
☐	☒	The statistical test(s) used AND whether they are one- or two-sided<br>*Only common tests should be described solely by name; describe more complex techniques in the Methods section.*
☒	☐	A description of all covariates tested
☒	☐	A description of any assumptions or corrections, such as tests of normality and adjustment for multiple comparisons
☐	☒	A full description of the statistical parameters including central tendency (e.g. means) or other basic estimates (e.g. regression coefficient) AND variation (e.g. standard deviation) or associated estimates of uncertainty (e.g. confidence intervals)
☒	☐	For null hypothesis testing, the test statistic (e.g. *F*, *t*, *r*) with confidence intervals, effect sizes, degrees of freedom and *P* value noted<br>*Give P values as exact values whenever suitable.*
☒	☐	For Bayesian analysis, information on the choice of priors and Markov chain Monte Carlo settings
☒	☐	For hierarchical and complex designs, identification of the appropriate level for tests and full reporting of outcomes
☐	☒	Estimates of effect sizes (e.g. Cohen's *d*, Pearson's *r*), indicating how they were calculated

Our web collection on statistics for biologists contains articles on many of the points above.

Software and code

Policy information about availability of computer code

Data collection	Attune Net software : 3.1.1243.0 software for flow cytometer
Data analysis	FlowJo v10.9.0 : commercial software for flow cytometry analysis<br>NucleusJ 2.0 : open source / custom ImageJ plugin<br>ImageJ : open source, image analysis<br>Biom3d : open source / custom, deep learning method for segmentation (https://www.biorxiv.org/content/10.1101/2024.07.25.604800v2)<br>OMERO.web 5.29.1 : open source, storage and figure assembly for microscopy images<br>CRESCENT v1.0: custom RNA-seq analysis (https://github.com/gilless429/crescent)<br>GraphPad Prism 11

For manuscripts utilizing custom algorithms or software that are central to the research but not yet described in published literature, software must be made available to editors and reviewers. We strongly encourage code deposition in a community repository (e.g. GitHub). See the Nature Portfolio guidelines for submitting code & software for further information.

Data

Policy information about availability of data

All manuscripts must include a data availability statement. This statement should provide the following information, where applicable:

- Accession codes, unique identifiers, or web links for publicly available datasets
- A description of any restrictions on data availability
- For clinical datasets or third party data, please ensure that the statement adheres to our policy

RNA-seq, ChIP-seq and Hi-C data are deposited under the record GSE304463 (https://www.ncbi.nlm.nih.gov/geo/query/acc.cgi?acc=GSE304463). The data can be accessed with the following token: urczuasepfgbhmb.
Source data underlying Fig.1c, Fig. 3b,c , Fig.4f, Fig. 6b,e,f, Fig. S1a-d, Fig. S2f,and Figure S6, Fig. S11c, Fig. S13c, Fig. S14b,a,g are accessible here: https://omero.mesocentre.uca.fr/webclient/?show=project-2803. Upon publication this link will become a permanent doi for durable accessibility.
Seeds are available under request.
All data supporting the findings of this study are available within the manuscript and its supplementary files.

Research involving human participants, their data, or biological material

Policy information about studies with human participants or human data. See also policy information about sex, gender (identity/presentation), and sexual orientation and race, ethnicity and racism.

Reporting on sex and gender	not applicable
Reporting on race, ethnicity, or other socially relevant groupings	not applicable
Population characteristics	not applicable
Recruitment	not applicable
Ethics oversight	not applicable

Note that full information on the approval of the study protocol must also be provided in the manuscript.

Field-specific reporting

Please select the one below that is the best fit for your research. If you are not sure, read the appropriate sections before making your selection.

☒ Life sciences ☐ Behavioural & social sciences ☐ Ecological, evolutionary & environmental sciences

For a reference copy of the document with all sections, see nature.com/documents/nr-reporting-summary-flat.pdf

Life sciences study design

All studies must disclose on these points even when the disclosure is negative.

Sample size	For plant phenotyping, we used at least 3 biological replicates for each genotype<br>For RNA-seq analysis, we used 3 biological replicates for each genotype.<br>For RT-qPCR, we used 3 biological replicates for each genotype.<br>For ChIP-qPCR we used 3 biological replicates<br>For H2B.8 ChIP-qPCR analysis, we used 2 biological replicates.<br>For WBs, we used >3 biological replicates, except for Supplementary Figure 11B.<br>For Hi-C we used 2 biological replicates.<br>For nuclei parameters determination, we used 2 replicates.<br>For quantification of EdU or H2B.8 positive nuclei, we used 2 replicates.
Data exclusions	not applicable
Replication	Number of biological replicates minimum of 2; qPCRs with minimum of 2 technical replicates
Randomization	No experimental groups involved in this study, thus randomization was not performed.
Blinding	For quantification of EdU or H2B.8 positive nuclei, we used a double blind setup.

Reporting for specific materials, systems and methods

We require information from authors about some types of materials, experimental systems and methods used in many studies. Here, indicate whether each material, system or method listed is relevant to your study. If you are not sure if a list item applies to your research, read the appropriate section before selecting a response.

Materials & experimental systems

n/a	Involved in the study
[ ]	[x] Antibodies
[x]	[ ] Eukaryotic cell lines
[x]	[ ] Palaeontology and archaeology
[x]	[ ] Animals and other organisms
[x]	[ ] Clinical data
[x]	[ ] Dual use research of concern
[ ]	[x] Plants

Methods

n/a	Involved in the study
[ ]	[x] ChIP-seq
[ ]	[x] Flow cytometry
[x]	[ ] MRI-based neuroimaging

Antibodies

Antibodies used

The following primary antibodies were used : monoclonal anti-Myc antibody (Millipore, 05-724, batch 3800004, IF 1/100, WB 1/2000, IP 5µg, ChIP 1µg), polyclonal anti-Myc antibody (Abcam, AB9106, batch 1058571-4, WB 1/1000, ChIP 1µg), anti-human H2B antibody (Abcam, ab1790, batch GR3373614-1, WB 1/2000), and anti-H3 antibody (Abcam, AB1791, batch GR3237738-1 and 1068998-2, WB 1/25000), anti-Flag (Abcam ab18230, batch GR229415-5, IF 1/100), anti-Flag (Sigma, F3165, batch SLCJ3741, WB 1/500), anti-H2A.Z (Agrisera AS10 718, batch 2410, IF 1/250) and anti-H3K27me3
(Diagenode, C15410069, batch A1818P, IF 1/100). Anti-Flag M2 magnetic beads (Sigma-Aldrich, M8823, batch SLBM0090V) were used in IP.
The anti-H2B.8 antibody was raised in rabbits (Eurogentec) against an AtH2B.8 N-terminal peptide (VSVTKKKKVVEETIK) and used at a 1/1000 dilution. Horseradish peroxidase-coupled anti-rabbit (Abliance, BI 2407, batch 241006, 1/5000) or anti-mouse (Abliance, BI 2413C, batch 231006, 1/5000) secondary antibodies were used in Western Blot and anti-mouse Alexa-594 (Invitrogen, A21203, IF 1/1000), and anti-rabbit Alexa-488 (Invitrogen, A11070, 1/1000). The anti-H2A.W antibody (IF 1/400) was generated against two peptides specific for the H2A.W.6 variant as in (Yelagandula et al., 2014).

Validation

ChIP grade commercial antibodies were used. For ChIP-seq and qPCR, specificity of Myc antibody was confirmed on wildtype seeds not expressing Myc-H2B.8.
For IF, when possible, antibodies detecting histone modifications were validated on mutant plants lacking the histone modification. Absence of unspecific binding of the secondary antibodies was confirmed by absence of staining without primary antibody.

Plants

Seed stocks

h2b.8-3 (SM_3_38239), hira-1 (WiscDsLox362H05) and ssrp1-2 (SALK_001283C) were obtained from the Nottingham Arabidopsis stock center

Novel plant genotypes

We generated the following transgenic plants:
WT plants expressing pH2B.8::4xMyc-H2B.8; WT plants expressing pH2B.8::H2B.8-3xFlag; WT plants expressing pH2B.8::H2B.8-GFP; WT plants expressing pHTR5::4xMyc-H2B.8; WT plants expressing pH2B.8::3xFlag-H3.3, pH2B.4::Flag-H2B.4.
We generated hira-1 h2b.8-3 double mutants through crossing of single mutants.
We generated a segregating F2 population by crossing pH2B.8::4xMyc-H2B.8 with pH2B.4::Flag-H2B.4

Authentication

For the mutants, we confirmed the genotype of the homozygous lines using PCR-based genotyping, including appropriate controls, with primer details provided in Supplementary Data 2.
For the transgenic lines expressing tagged histone proteins, homozygous T3 lines or seeds from independent T1 plants were used.

ChIP-seq

Data deposition

[x] Confirm that both raw and final processed data have been deposited in a public database such as GEO.

[x] Confirm that you have deposited or provided access to graph files (e.g. BED files) for the called peaks.

Data access links
May remain private before publication.

https://www.ncbi.nlm.nih.gov/geo/query/acc.cgi?acc=GSE304463

Files in database submission

GSE304459_INPUT_MYC.sorted.bw, GSE304459_IP_Myc_1_H2B8.sorted.bw, GSE304459_IP_Myc_2_H2B8.sorted.bw and all fastq files for INPUT and IP.

Genome browser session
(e.g. UCSC)

Provide a link to an anonymized genome browser session for "Initial submission" and "Revised version" documents only, to enable peer review. Write "no longer applicable" for "Final submission" documents.

Methodology

Replicates

Two biological replicates made from a pool of 4-5 biological replicates precipitated either with a polyclonal or a monoclonal anti-Myc

	antibody.
Sequencing depth	total reads : rep1 :25201683, rep2 : 25300458,INPUT :25298879 / uniquely mapped : rep1 :16417516, rep2 : 15510719,INPUT : 16220857 / lenght of reads: 50/ single-end
Antibodies	mouse monoclonal anti-Myc (Millipore, 05-724) or rabbit polyclonal anti-Myc (Abcam, AB9106)
Peak calling parameters	bowtie2 -x TAIR10.index --very-sensitive -p 20 -U file.fq.gz -S file.sam<br>macs2 callpeak --bdg -f BAM -g 120e6 --nomodel --broad --qvalue 0.01 --bw 300 -t IP.bam -c INPUT.bam -n IP_vs_input_MACS2_q001
Data quality	We performed the peak calling seperately for each replicate and only retained peaks found in both replicates for further analysis.
Software	bowtie2 , picard-tools, samtools, MACS2,

Flow Cytometry

Plots

Confirm that:

☒ The axis labels state the marker and fluorochrome used (e.g. CD4-FITC).

☒ The axis scales are clearly visible. Include numbers along axes only for bottom left plot of group (a 'group' is an analysis of identical markers).

☒ All plots are contour plots with outliers or pseudocolor plots.

☒ A numerical value for number of cells or percentage (with statistics) is provided.

Methodology

Sample preparation	Fifty seeds were harvested at the different time points, and the embryos were dissected and then chopped in 100µL of cold GB buffer (45mM $MgCl_2$, 30mM Sodium citrate, 20mM MOPS pH 7, 0.1% Triton X-100, 1% BSA). Samples were then filtered through a 30µm CellTrics filter and fixed in 2% formaldehyde in GB. To purify the nuclei, the filtrate was deposited on top of an Eppendorf tube containing 100µL of 2M sucrose, layered over GSB buffer (0.5M sucrose, 0.1% BSA, 15mM Sodium Citrate, 10mM MOPS pH 7, 0.15% Triton X-100, 0.5% PVP, 0.5% BSA, 22.5mM $MgCl_2$). Samples were then centrifuged for 4 min at 4000 g with minimal braking. The nuclei, localized in the GSB layer, were collected and diluted in GB buffer with 5µg/mL of DAPI for a final volume of 1mL and analyzed on an Attune™ NxT Acoustic Focusing Cytometer.
Instrument	Attune™ NxT Acoustic Focusing Cytometer model BRVY, part number: 4486521, serial number: 2AFC215830517
Software	FlowJo v10.9.0
Cell population abundance	*Describe the abundance of the relevant cell populations within post-sort fractions, providing details on the purity of the samples and how it was determined.*
Gating strategy	Gating examples in figure S13a.

☒ Tick this box to confirm that a figure exemplifying the gating strategy is provided in the Supplementary Information.
