# Supplementary material for: Replication-independent eviction of the histone variant H2B.8 reveals chromatin reprogramming during seed imbibition
Source: Nat Commun. 2026 Jun 29;17:8102. doi: 10.1038/s41467-026-74230-6 (PMC13458839; doi:10.1038/s41467-026-74230-6)
Supplement: Supplementary file 3 [file 41467_2026_74230_MOESM9_ESM.pdf]

nature portfolio

Peer Review File

Replication-independent eviction of the histone variant H2B.8 reveals chromatin reprogramming during seed imbibition

Corresponding Author: Dr Aline Probst

This file contains all reviewer reports in order by version, followed by all author rebuttals in order by version.

Version 0:

Reviewer comments:

Reviewer #1

(Remarks to the Author)
In this study, the authors find that H2B.8 accumulates in dry seeds to maintain a compact chromatin structure and marks euchromatic transposons and low expressed genes. Especially, H2B.8 marks regions enriched with H3K27me3 or gene-body H2A.Z. Furthermore, they demonstrate that H2B.8 was removed by downregulation of H2B.8 protein abundance during imbibition. In which way, the dynamic changes of H2B.8 function as a key regulator of seed to seedlings transition in plants. Overall, the conclusions are supported by the results and the findings are significant. I have several comments.

Major
1. Figure 2C, although there is a non-specific band, it is still necessary to include a loading control (for example H3) in these blots in order to confirm that the antibody detects H2B.8 specifically.
2. The predicted size for H2B.8 is 27 kD. However, the authors detected a band at about 40 kD. They explain this discrepancy by expressing full-length H2B.8, H2B.8-tail, and H2B.4 in yeast to exclude the effect of PTM. However, the possibility that H2B.8 can be similarly modified in yeast as in plants cannot be totally ruled out. Thus, it is difficult to draw the conclusion that the mobility shift is caused only by sequence. The authors may need to express H2B.8 in E.coli and purify the protein for western blot analysis. Moreover, although endogenous H2B.8 could shift to a size that is much larger than its predicted size, how do the authors explain a 4×Myc tag fusion would cause 15-20 kD difference in size? How about H2B.8-Flag protein?
3. According to Figure S3C, the MNase digestion is not complete. This could make the conclusion that H2B.8 forms heterotypic nucleosomes less convincing, if no further evidences are provided. Because the H2B signal observed could potentially result from di- or oligo-nucleosomes that retained. Furthermore, I think, a blot using anti-H2B.8 should be shown, to compare with the blots using a generic H2B antibody.
4. Clearly, the H2B.8 mRNA and protein abundance are downregulated during seed imbibition, likely due to decreased transcription of H2B.8 gene. But it is difficult to say that imbibition triggers degradation of H2B.8 transcript (line 41). The authors have to examine the stability of H2B.8 mRNA to make this conclusion.
5. CHX blocks global protein translation and synthesis. It is confusing to me that why CHX treatment slowed down H2B.8-GFP signal decrease in Figure 6E. What's the results if the authors perform the same treatments, but detect with western blots?
6. The evidences for eviction of H2B.8 is weak. The authors may need to monitor the chromatin associated H2B.8 protein levels (in Figure 4D, Figure 5A, and Figure S11B) to better reflect eviction, not just protein turnover or gene silencing of H2B.8 during imbibition. Current results clearly support the downregulation of H2B.8 protein abundance, which will of course limit its deposition. But more direct evidences for histone eviction of H2B.8 is required.
7. A previous study of H2B.8 in sperm suggests that H2B.8 does not regulate transcription (Buttress et al., 2022), the data in this study demonstrate a quite different conclusion that H2B.8 could regulate the expression of about 921 genes during imbibition. But no clear discussion or explanation could be easily found in the manuscript.

Minor
1. Figure 2C, the western blot quality needs improvement to show an even background.
2. Figure 2B and 2C is confusing, they actually should be put together in the same panel.
3. Could the authors compare the relative association affinity of H2B.4-H2B.4, H2B.4-H2B.8, and H2B.8-H2B.8? This could be another support for the formation of H2B.8 heterotypic nucleosomes.

4. Molecular weight markers are missing in several WB results, such as Figure 4D.
5. In Figures 1F–G, Please specify in the Materials and Methods how “H2B.8-marked” and “H2B.8-free” genes were defined, including any thresholds applied.
6. Figure 4A: H2B.4 is used in the AlphaFold nucleosome model, whereas the RT-qPCR uses H2B.2. Why?
7. Line 253, the claim that global chromatin accessibility may require ATAC-seq or MNase-seq in addition to agarose gels.
8. Figure 5A: The H3 loading control varies across lanes. Please optimize the blot.
9. Figure 6E, middle panel, “hypocotyl”, not “hypocot”?

Reviewer #2

(Remarks to the Author)
The manuscript by Simon et al. entitled “Replication-independent eviction of H2B.8 reveals chromatin reprogramming during seed imbibition” identifies and addresses the role of the histone variant H2B.8 in the see-to-seedling transition. The histone variant H2B.8 has previously been connected to sperm cell compaction in flowering plants (Buttress et al. 2022). In this manuscript, the authors show that H2B.8 is expressed during seed maturation, contributes to chromatin organization in dry seeds and is transcriptionally repressed and removed during seed imbibition. Under lab conditions, the described presence and dynamics of H2B.8 does not seem to affect seed germination. Using ChIP-seq, RNA-seq and Hi-C of dry seeds, the authors show that H2B.8 marks inactive genes and chromosome-arm TEs and contributes to spatial contacts of specific euchromatic domains. While the absence of H2B.8 (in h2b.8 mutant) has almost no impact on gene transcripts in dry seeds, it contributes to the transcriptional activation of a subset of H2B.8-marked genes during seed imbibition. Using promoter swaps, the authors demonstrate that H2B.8 can be deposited by general histone chaperone systems and that the histone eviction during seed imbibition is not exclusive to H2B.8 but involves also H3.3. Finally, using different chemical treatments, the authors show that H2B.8 is evicted in a manner that is independent on DNA replication but involves proteasome degradation and it promoted by transcription and protein translation.
This is a well written manuscript, summarizing carefully designed, conducted and critically interpreted experiments. Given the absence of physiological phenotypes, the role of H2B.8 in the seed and during seedling emergence is not entirely clear and the phenotypes are mostly correlative. Nevertheless, the study is in my opinion of high value, newly identifying the involvement of H2B.8 in seed development, providing novel insights into histone dynamics during the seed-to-seedling transition.
I have several comments and questions to the authors:
1. It is not clear whether H2B.8 contributes to general chromatin structure in the seed and/or may have a regulatory role. While some results indeed point to a repressive role (e.g. Figure 1I), possible regulatory role is suggested by selective targeting to a subset of genes (Fig 1E, Fig.S2); progressive activation of H2B.8-marked genes (Fig. 4F) and downregulation of H2B.8-marked genes in h2b.8 (Fig. 4G). What genes are affected and may this support a regulatory role of H2B.8?
2. The authors elegantly use tools that prevent the depletion of H2B.8 upon imbibition (e.g. proteasome inhibitors – Fig. S13) or even promote re-establishment of H2B.8 after germination (pHTR5::H2B.8 – Fig. 5A). What is the impact of these manipulations on plants and H2B.8-marked gene expression?
3. Lines 237 – 255, Figures 3 and S6: The volume of the nucleus is moderately increased in h2b.8 and hira, and the phenotype is additive in the combined mutant. The volume of intensely stained regions is increased in h2b.8 and the combined mutant but reduced in hira. The impact of h2b.8 is explained as being dominant over hira (Lines 254 – 255). Higher volume of intensely-stained regions may indicate higher tendency to form the compact regions but also their loosening (incomplete/disabled compaction), i.e. two seemingly opposite phenomena. Hence, one could also interpret the results a rescue of hira by h2b.8. Can the authors clarify the interpretation of the phenotype?
4. The level of H2B.8 marking positively correlates with local chromatin contacts (Fig. 3F). Is this reflected in differential activation of the underlying genes upon imbibition/germination?

Other comments:
5. Line 97 – “Besides sperm nuclei…” – instead of “Besides in sperm nuclei…”
6. Line 129 – “strongly visible?” instead of “strongly enriched”? Enrichment suggests comparative amount in relation to something else….
7. Line 129 – Figure 1a – H2B.8 seems depleted form the SAM (compared to its level in other tissues and to H3.3) – is this so and can the authors comment on this?
8. Line 139 - Figure S1A and B: the localization of H2B.8 seems to be peripheral using the Myc-H2B.8 (A) but more centrally located in the H2B.8-Flag line (B). Do the differently tagged H2B.8 give different distribution pattern and why? Can the authors comment on this?
9. Lines 150-161 – There seem to be some discrepancies in the numbers of genes targeted by H2B.8: Text (line 150) states 7749 genes (same in Fig S2c,e and Fig 1e lower venn) but Fig. 1E upper venn depicts 8179 genes, and legend to Fig. 1f,g states 7549 genes). Can you explain/correct this?
10. Figure 1f,g – It is not entirely clear from the figures of the legends what is depicted: is it all annotated genes separated by being/or not marked by H2B.8 OR H3K27me3/H2A.Z targets separated by being or not marked by H2B.8? The logic suggest it is the former/first option but it should be made clear by the legend, including stating the number of genes that are NOT H2B.8 targets.
11. Figure 1f – the enrichment profile lines are relatively thin and poorly visible.
12. Line 1072 (Legend and Fig 2b,c) – (i) it is not clear what input 1/5 or 1/10 means, (ii) it would ease reading the figure if the antibodies used for detection were specified in the figure and (iii) formulation: “Revelation with anti-Myc…reveals” – instead “Detection using anti-Myc or other noun?
13. Line 314 – 315: “Given the substantial co-enrichment of H2B.8 and H3K27me3….” – the statement refers to Fig. 1E? Please clarify/provide reference.

14. Line 327 should refer to Fig S12C instead of S13C and the figure reference would be better suited after the previous sentence on line 326. For clarity, Figure 4G would be better referred to upon first mentioning of the numbers of genes changed (line 327).
15. Line 388: "…exposed imbibed seeds TO continuous light"
16. Line 388: Evidence/reference to figure is missing to support the statement that "….,25 – 57% of the seeds germinated after 48h across 3 biological replicates."
17. Line 523: "….C-TERMINAL lysine"
18. Line 1189 – legend to Fig S2: "H3K4me3-marked genes"

Reviewer #3

(Remarks to the Author)
I co-reviewed this manuscript with one of the reviewers who provided the listed reports. This is part of the Nature Communications initiative to facilitate training in peer review and to provide appropriate recognition for Early Career Researchers who co-review manuscripts.

Reviewer #4

(Remarks to the Author)
There are two main messages in this manuscript by Simon et al. The first one is focused on analysing the nature and functions of the H2B.8 variant, which has a longer amino terminal domain as well as some amino acid changes and is abundant in seeds, both in dry seeds and during the seed-to-seedling transition. The other part of the manuscript investigates the chromatin reprogramming during the seed-to-seedling in Arabidopsis.

I will start highlighting the strengths of the manuscript: the experimental part has been thoroughly designed with a clear interest in detail and overall presenting an array of well performed experiments (see below for more specific comments that need to be addressed). The authors profile the seed-enriched histone variant H2B.8 using tagged lines, a custom antibody, ChIP-seq, Hi-C, imaging/segmentation, RNA-seq (with spike-in at 24hD), and S-phase timing by EdU/FACS. The manuscript is in general well written, with only a few details that will benefit from some polishing (see below in minor comments). The original ideas, understanding the role of H2B.8 in seeds and the seed-to-seedling nuclear reprogramming, and their crosstalk, are attractive and will be of interest not only for the plant community, but also for a broader scientific community focus on understanding how transcriptional changes linked to development are governed. Despite these undeniable strengths, the manuscript deals with an important caveat: the lack of functional results for H2B.8 that prevents the manuscript from driving final conclusions and answering the following question: why do Arabidopsis seeds need to accumulate this particular histone variant? The authors showed that H2B.8 accumulates in dry embryos, forms heterotypic nucleosomes (I find this part and the biochemical analysis of the H2B.8 variant particularly compelling!), maps to lowly expressed genes and euchromatic TEs (often co-marked by gene-body H2A.Z and H3K27me3), and that H2B.8-rich regions cluster in 3D. Upon seed imbibition, H2B.8 transcripts drop and the protein disappears from the nuclei in a tissue-ordered manner and at 24hD, a subset of H2B.8-marked genes fails to activate in h2b.8. Still, all these results are descriptive and insufficient to address that initial question for the need to accumulate this variant in seeds. On the other hand, the results allow them to propose a model where water uptake triggers replication-independent histone turnover that reconfigures 3D architecture to enable early gene activation.
Here some more specific details that I would like to discuss with the authors and that, whether addressed, may help to improve the manuscript:

-why do the authors describe the reduction of H2B.8 from the chromatin as eviction? I don't think that they present data to check whether the protein is evicted from the chromatin during the transition from seed to seedling. However, they present robust data that shows that 1) the H2B.8 transcript amount decreases during germination (even showing an explanation for this decline due to H3K27me3 high signal on the H2B.8 locus) and 2) the H2B.8 protein levels decline most probably degraded by the proteasome, which may be key for the histone turnover during this developmental transition. To check for eviction, so removal from the chromatin, the authors would have needed to separate the pool of H2B.8 associated to the chromatin from the free pool, something that they didn't do and I don't think they need to check because they have enough results to understand why H2B.8 levels decline during germination. Please, reconsider the wording avoiding the term "eviction". In addition, considering that protein degradation seems to have an important effect on H2B.8, I would strongly recommend moving supplementary figure 13A-B into main figure 6. Indeed, a possibility that cannot be discarded is that protein synthesis/transcriptional onset is indeed needed for synthesis of the protein degradation machinery to allow H2B.8 degradation.

-my next comment is related to the previous one. I think that the authors had the hypothesis that histone eviction mediated by histone chaperones played a major role in H2B.8 abundance. I do agree that their results with the ssrp1-2 mutant partially supports this idea (there is only a partial reduction in H2B.8 levels). Therefore, this could indicate that FACS is important for its deposition on the chromatin, but still, it does not explain how the decline in its levels occur during germination. I also think that the authors focused too much on the idea of eviction/turnover and miss a clear opportunity to understand the proteolytic regulation of H2B.8. Furthermore, what happens with the Flag-H3.3 binding to the chromatin during imbibition? I agree that its amount slightly decrease probably due to the H2B.8 promoter being repressed, but it doesn't seem to decline as much as H2B.8 protein levels. Therefore, I would expect that the Flag-H3.3 protein can still be detected during imbibition by ChIP-PCR to fully understand how much de novo deposition occurs. This would indicate that there is another factor in the equation: protein degradation that requires the H2B.8 protein and doesn't occur efficiently in the Flag-H3.3 line. The

argument that “eviction” precedes DNA replication is very interesting but requires stronger quantitative backing. In Fig. 6A, please provide replicate counts, gating strategy, coefficients of variation for the 2C peak, and quantitative cell-cycle parameters (e.g., %G1, %S, %G2 ± CI) at each timepoint. Clarify how endoreduplication is distinguished from mitotic S-phase in this context. For Fig. 6B, report the % of EdU nuclei per tissue per timepoint, with embryo and nucleus counts and appropriate statistical tests. Together, these data would make the timing of S-phase onset explicit. Finally, authors could include a one-sentence conclusion in the Results stating clearly that the decline in H2B.8 (≤24hD) occurs before the first detectable EdU^+ nuclei (48hD24hL), thereby preceding DNA replication.

-still related with H2B.8 “eviction”, in figure 4 several results are only partially quantified. In panel A, transcript dynamics are shown and a Friedman’s test is reported ($p<0.05$), but the number of biological replicates (n), the definition of error bars (SD vs. SEM), and exact p-values are not provided. In panel D, the quantification suggests four biological replicates, but only three uncropped blots are shown in the supplementary, replicate numbers per condition are inconsistent (3–4 dots), and the stability of the H3 loading control is not addressed. Furthermore, only normalized values are presented, without effect sizes or confidence intervals to define the magnitude and precision of the ~50% reduction at 24hD. In panel E, GFP imaging is descriptive, lacking per-nucleus quantification, embryo/nucleus counts, and statistical testing. Finally, the term “rapid” is used but not defined quantitatively (for example “≥50% reduction in cotyledon nuclei by 24hD”). Providing per-tissue quantification across time points, with exact replicate numbers, effect sizes, confidence intervals, and explicit thresholds for “rapid eviction”, will be essential.

-it is indeed very unfortunate that neither the h2b.8 mutant nor its transgenic lines (overexpressing line or under the H3.3 promoter) showed any obvious phenotypic changes under the analysed conditions. However, I think that the authors could have checked a few more things. For instance, in the discussion they commented that “deficient germination or reduced seed longevity might only be observed in nature”. However, this can be easily mimicked and analysed in the lab to check if seed longevity is affected in the h2b.8 mutant or the overexpressing lines. They also mentioned that “when seeds are imbibed under suboptimal conditions and enter secondary dormancy, H2B.8 expression is induced”, did they check dormancy in the mutant or overexpressing lines? In addition, repeated imbibition – desiccation, or suboptimal germination conditions (in the presence of GA inhibitors or chemicals) could have been easily tested. As an argument for the lack of obvious phenotype they discussed about “The absence of a germination phenotype is consistent with the h2b.8 mutants being fertile, and the specific phenotypic differences observed for the loss of other histone variants under standard growth conditions such as the sperm-specific H3.1 or H3.14 and H3.15, which are induced in roots upon stress or wounding”. However, as far as I know, when mutants affected in these variants are grown under specific conditions, there are observable phenotypic changes. Therefore, this will indeed indicate that the authors are missing the right conditions to observe the h2b.8-related phenotype that would be crucial to understand its molecular functions.

-The RNA-seq analysis shows that H2B.8-marked genes tend to activate upon imbibition and that their induction is impaired in h2b.8 mutants. However, the time course currently comes from an external dataset, while the genotype effect is presented only at 24hD. To strengthen the narrative under your own growth conditions, please include a short RT-qPCR panel across DS → 24hD → 48hD (±48hD24hL) for some H2B.8 target genes. In addition, to establish that the overlap in Fig. 4G–H is not incidental, provide enrichment statistics (odds ratio with 95% CI, Fisher’s exact p) and a distribution-level test (log2FC shift, Wilcoxon). This will demonstrate that the overlap between down-regulated genes and H2B.8-marked genes is more than expected by chance. It is interesting that despite the impairment of that set of genes during germination, the mutant still germinates well. Can the authors analyse GO terms for both H2B.8 target genes and H2B.8 misregulated target genes? This may provide some clues about its functional role and perhaps the most appropriated conditions to further test for phenotype.

- Figure 6E shows that H2B.8 “eviction” is impaired by CHX and α-amanitin, but the evidence is qualitative. To support the conclusion that this requires de novo transcription/translation, please provide per-nucleus retention metrics stratified by tissue and timepoint, with embryo/nucleus counts, effect sizes, confidence intervals, and exact p-values. Because both inhibitors can impair general physiology, viability/germination controls, experiments are needed to exclude nonspecific toxicity. Finally, since α-amanitin has specificity and uptake caveats, a second transcription inhibitor would strengthen the claim; if not feasible, this limitation should be noted with justification of dose and conditions.

-the authors discussed that more in vitro studies will be needed to fully understand the impact of H2B.8 on the nucleosome. However, could the HiC data help to support any of the two possible scenarios, that H2B.8 positively or negatively affects DNA-octamer interactions? Did they try micrococcal nuclease analyses in the WT vs the mutant to see whether access to nucleosomal DNA could be affected? I do agree that for other functional analyses (e.g. use of deletions and point mutations) deeper and time-consuming approaches will be needed.

-I would strongly recommend unifying the experimental timeline and conditions across the paper. Readers, like this reviewer, will follow the biology more easily with a single, consistent timeline from dry seeds through imbibition and transfer to light. Please add a schematic used consistently in Figs. 4–6: DS → 24hD → 48hD → transfer to light → 48hD24hL → 48hD48hL → 48hD72hL, plus the continuous-light (cL) branch. Define hL (“hours after transfer to long-day light”) and cL (“continuous light”) at first use in the main text and repeat in relevant legends. Mark the transfer-to-light boundary directly on plots that span dark→light. Also resolve the inconsistency “48 h at 4 °C” (Results) vs “48 h at 6 °C” (Methods) and propagate the chosen value throughout text, legends, and Methods.

Other minor comments:
1. Textual edits
• “may may favor H2Bub” → “may favor H2Bub.”
• Ensure consistent temperature notation and spacing: 23 °C, 4 °C (number + thin space + °C).

• Supplementary Data numbering: “Supplementary Data 6” appears twice—please renumber to avoid duplication.
• Introduction line 78: “FACILTATES CHROMATIN TRANSCRIPTION (FACT) ensure” → “FACILITATES CHROMATIN TRANSCRIPTION (FACT) ensures.”
• Fig. 2 text: “b-sheets” → “β-sheets”; “alpha” → “α”. Please, Use standard Greek letters in text (α-amanitin).
• Discussion line 524: “may may favor H2Bub enrichment” → “may favor H2Bub enrichment.”
• Suppl. Fig. 12 legend: “genes that loose or gain H3K27me3” → “genes that lose or gain H3K27me3.”
• Methods line 554 and fig. 6 legend-Imbibition/Inhibitors: “Cycloheximidine” → “Cycloheximide” (match earlier Results spelling).
• Methods line 709- Chromatin shearing: “chromatin was shared using a Covaris” → “chromatin was sheared using a Covaris.”
• Discussion lines 473-476: this should be at least mentioned in the results part. As a reader, I thought this information was missing from the results.
• Discussion lines 475-476: please, add “this finding indicates that, under our standard growing conditions,…”
• Discussion line 460-461: It doesn’t read well. Please, change to “…with the H2A.Z and H3K27me3 co-enrichment in gene body,...'.
• Results lines 326-328: authors mentioned Figure S13c. Do they mean Figure S12c?
• Results line 323: “…a subset of genes” reads vague. I know that the specific numbers are in the figure, but it would be more informative to add them to the text.
• Figure 4b: it took me a while to realise which gene corresponds to H2B.8 as, I think, this is the first time it is mentioned with the code. It would also be nice to indicate the orientation of the genes to understand which intergenic region corresponds to the promoter.
• Add a short, first-use definition of “heterotypic nucleosome”.
• Maintain consistent unit spacing (e.g., 100 µM, 20 µm, 25 °C) and symbol usage across main and Supplementary.

2. Statistics, legend
• For every figure/panel, state exact n (distinguish biological vs technical), define error bars (SD/SEM/95% CI), and report exact p-values with the named test (and any multiple-testing correction).
• Where summaries are shown, add per-replicate dots and effect sizes with 95% CIs.

3. Data
• Provide a figure-to-data map (table: panel → raw data files → analysis script/software + version) and link it to the existing OMERO/GEO entries.
• In Data availability, if its pointed towards readers to this mapping table as the single place to find all files, it might be very easy and helpful.
• Lines 182-185: “Both fusion proteins migrate at a similar molecular weight and slower compared to the H2B.4 variant (Figure S3b), revealing that, in dry seeds, the reduced electrophoretic mobility of H2B.8 is conferred by the tail sequence rather than by post-translational modifications”. Why? Can one fully discard the possibility that well conserved post-translational modifications occur in yeast? Or am I missing something?

Version 1:

Reviewer comments:

Reviewer #1

(Remarks to the Author)
The authors have addressed most of my concerns. The revised manuscript provided substantial evidences, linking the behavior of H2B.8 to epigenetic reprogramming and gene expression during seed imbibition. Again, the manuscript is well-written and most of the conclusions are supported by the data. Revisiting my previous concerns, I still recommend the authors to pay attention to the conclusion that H2B.8 forms heterotypic nucleosomes. After which, I think the conceptual advance of this work merits its acceptance for publication at Nature communications.

Here is my remaining concern:
It is still not convincing enough for me that H2B.8 forms heterotypic nucleosomes. I mean, yes, it could form heterotypic nucleosomes; but, is it the only form of nucleosomes that H2B.8 forms? The current evidences did not exclude the possibility that it could form homo. However, in the results part (Lines 195-207), the description gave an impression that it only forms heterotypic nucleosomes, which is very strong. I noticed the IP/input ratio, but have different opinion based on the data in Figure 2b. It looks like for me that the IP/input ratio for H2B is smaller than H3, by eye. In Osakabe et al., 2018, the authors’ conclusion is the majority of H2A nucleosomes are homogenic, but they also observed the potential heterogenic forms for H2A-H2A.W and H2A.X-H2A.W as well as H2A.W.7-H2A.W.6. Taking that into account, I think a more inclusive conclusion would be more acceptable. For experiments that I previously requested, to avoid heavy chain interference, the authors may consider using anti-Myc antibody from a different species than that used for generation of anti-H2B.8 for IP or they may want to use a secondary antibody that only recognizes the light chain. Otherwise, the authors should try to avoid such strong claim in Abstract, sub-titles, and Figure titles.

Others:
Line 45-46, Abstract, I believe this sentence is grammatically incorrect.

Reviewer #2

(Remarks to the Author)
This is a revised version of a manuscript that I had reviewed before. I have read through the manuscript and confirm that all my comments have been addressed to my satisfaction and to what I think is within the scope of this work.

Reviewer #3

(Remarks to the Author)
I co-reviewed this manuscript with one of the reviewers who provided the listed reports. This is part of the Nature Communications initiative to facilitate training in peer review and to provide appropriate recognition for Early Career Researchers who co-review manuscripts.

Reviewer #4

(Remarks to the Author)
I would like to thank the authors for their efforts to reply to my and other reviewers' concerns. I can confirm that they have carefully considered all reviewers' comments to provide an improved version of their manuscript:
• One of my main concerns regarding the claim of H2B.8 eviction is now better supported by ChIP-qPCR experiments of selected candidate genes showing loss of chromatin-associated H2B.8 during imbibition.
• The replication-independence claim is now backed with quantified FACS/EdU support in Supplementary Figure, and the text explicitly states that H2B.8 decline precedes the first detectable EdU-positive nuclei.
• The inhibitor experiments are no longer just qualitative: they now quantify retention under MG132 and α-amanitin, add TTC viability controls, and they have removed the stronger cycloheximide-based translation claim once viability failed.
• They also strengthened the transcriptional part by adding enrichment/GO support for the H2B.8-marked downregulated genes.
• I still consider that the lack of phenotype of the mutant and transgenic lines makes very difficult to drive final conclusions, but again this is not due to the lack of efforts from the authors. Future experiments as the one they mention (functional analyses in other species such as B. napus and more physiological analyses such as the response to hypoxia) may help to set some light. However, I do consider that this caveat still needs to be clearly stated and discussed in the manuscript.
• Although the H3.3 / global chromatin reprogramming angle is improved, I still feel that this part is less mechanistically developed than the H2B.8 story. For example, the manuscript shows that seed-deposited Flag-H3.3 declines during imbibition and that total H3 occupancy is maintained at tested loci, but it still does not directly resolve how much of this reflects true H3.3 eviction versus recycling/redeposition. In their response, the authors indicate that deeper analysis of H3.3 dynamics will be part of future work. I therefore think that the broader conclusions regarding H3.3/global chromatin reprogramming should be toned down further so that they remain fully aligned with the data currently shown.
• There are still a few editorial/polish issues. For example, the abstract still contains the wording error "thereby initiating and therefore initiates." I would also recommend one more careful language edit throughout, as there are still a few sentences that read as slightly overcomplicated or repetitive.

Open Access This Peer Review File is licensed under a Creative Commons Attribution 4.0 International License, which permits use, sharing, adaptation, distribution and reproduction in any medium or format, as long as you give appropriate credit to the original author(s) and the source, provide a link to the Creative Commons license, and indicate if changes were

made.
In cases where reviewers are anonymous, credit should be given to 'Anonymous Referee' and the source.
The images or other third party material in this Peer Review File are included in the article’s Creative Commons license, unless indicated otherwise in a credit line to the material. If material is not included in the article’s Creative Commons license and your intended use is not permitted by statutory regulation or exceeds the permitted use, you will need to obtain permission directly from the copyright holder.
To view a copy of this license, visit https://creativecommons.org/licenses/by/4.0/

We thank the four reviewers for their detailed evaluation of our manuscript and their suggestions and criticisms, which helped us to improve the manuscript. In the following you can find a point-by-point response to the issues raised:

Reviewer 1

In this study, the authors find that H2B.8 accumulates in dry seeds to maintain a compact chromatin structure and marks euchromatic transposons and low expressed genes. Especially, H2B.8 marks regions enriched with H3K27me3 or gene-body H2A.Z. Furthermore, they demonstrate that H2B.8 was removed by downregulation of H2B.8 protein abundance during imbibition. In which way, the dynamic changes of H2B.8 function as a key regulator of seed to seedling transition in plants. Overall, the conclusions are supported by the results, and the findings are significant. I have several comments.

We thank the reviewer for the insightful comments.

Major

1. Figure 2C, although there is a non-specific band, it is still necessary to include a loading control (for example H3) in these blots to confirm that the antibody detects H2B.8 specifically.

We consider that there may be a typo in the comment and that the reviewer might be referring to Figure 2a. We have replaced the blot with a new one and included an H3 loading control (*new Figure 2a*).

2. The predicted size for H2B.8 is 27 kD. However, the authors detected a band at about 40 kD. They explain this discrepancy by expressing full-length H2B.8, H2B.8-tail, and H2B.4 in yeast to exclude the effect of PTM. However, the possibility that H2B.8 can be similarly modified in yeast as in plants cannot be totally ruled out. Thus, it is difficult to draw the conclusion that the mobility shift is caused only by sequence. The authors may need to express H2B.8 in *E. coli* and purify the protein for western blot analysis.

To address this question as well as the concerns raised by Reviewer 4, we have now expressed the tail of H2B.8 in *E. coli* as a GST fusion. In agreement with our findings *in planta* and in yeast, we see that the H2B.8-tail GST fusion protein (expected size 43 kDa) migrates at about 60 kDa). This blot has now been added to Supplementary Figure 3 (*now Figure S3c*).

Moreover, although endogenous H2B.8 could shift to a size that is much larger than its predicted size, how do the authors explain a 4×Myc tag fusion would cause 15-20 kD difference in size? How about H2B.8-Flag protein?

We have now included in Figure 2a a *pH2B.8::H2B.8-(3x)Flag* line to compare directly the shift induced by the Flag and 4xMyc tag. We have previously observed, using a published *H3.3-4xMyc line* (Stroud, PNAS, 2012) that the 4x-Myc tag indeed induces a substantial size shift in SDS-page gels.

3. According to Figure S3C, the MNase digestion is not complete. This could make the conclusion that H2B.8 forms heterotypic nucleosomes less convincing, if no further evidence

is provided. Because the H2B signal observed could potentially result from di- or oligo-nucleosomes that retained. Furthermore, I think, a blot using anti-H2B.8 should be shown, to compare with the blots using a generic H2B antibody.

Indeed, a small fraction of dinucleosomes remain, however the majority are mononucleosomes. We have done several tests to optimize the MNase concentration and digestion time, and we found that these conditions are optimal to avoid that MNase starts digesting nucleosomal DNA, then strongly affecting efficiency of the immunoprecipitation. Our digestion profiles closely resemble those shown in Figure 1 of Osakabe et al. (NAR, 2018), which were also used to draw conclusions about the homo- or heterotypic nature of the nucleosomes. We want to draw your attention that the IP / input ratio is similar for H3 and total (small) H2Bs, which would suggest that we find predominantly heterotypic nucleosomes.

Unfortunately, it is not possible to reveal the blots in Figure 2B with the anti-H2B.8 antibody due to cross-reaction with heavy chain IgG, as the H2B.8 protein runs at the same height.

To show that heterotypic nucleosomes indeed exist we have generated another transgenic line expressing Flag-H2B.4 under control of its endogenous promoter and crossed it with the pH2B.8::Myc-H2B.8 lines. Using segregating F2 seeds, we immuno-precipitated Flag-H2B.4 containing nucleosomes which indeed can contain Myc-H2B.8. These data are now included as new *Supplementary Figure 3f, g* with the corresponding MNase digestion profiles in *Supplementary Figure 3e*.

4. Clearly, the H2B.8 mRNA and protein abundance are downregulated during seed imbibition, likely due to decreased transcription of the *H2B.8* gene. But it is difficult to say that imbibition triggers degradation of the *H2B.8* transcript (line 41). The authors have to examine the stability of *H2B.8* mRNA to make this conclusion.

We agree with the reviewer that we do not have final proof, even though mRNA degradation has been suggested to occur during seed germination (e.g. Basbouss-Serhal, et al., 5′ to 3′ mRNA decay contributes to the regulation of Arabidopsis seed germination by dormancy, Plant Physiol., 173 (2017), pp. 1709-1723). We have removed this statement from the abstract.

5. CHX blocks global protein translation and synthesis. It is confusing to me that why CHX treatment slowed down H2B.8-GFP signal decrease in Figure 6E. What's the results if the authors perform the same treatments, but detect with western blots?

Unfortunately, these experiments cannot be carried out by Western Blot, as, to ensure that the inhibitors correctly enter the seed, we must manually make holes in each seed to ensure CHX entry and this is not feasible with a high number of seeds (100mg) required for Western Blot.

We have now also analyzed seed viability by 2,3,5 triphenyl tetrazolium chloride (TTC) staining of the seeds treated with the different inhibitors. Seeds treated with Cycloheximide are no longer metabolically active (now *Supplementary Figure 14h*). Based on these results we have decided to remove the statement from the manuscript / abstract that translation inhibits H2B.8 eviction.

6. The evidence for eviction of H2B.8 is weak. The authors may need to monitor the chromatin associated H2B.8 protein levels (in Figure 4D, Figure 5A, and Figure S11B) to better reflect eviction, not just protein turnover or gene silencing of H2B.8 during imbibition. Current results clearly support the downregulation of H2B.8 protein abundance, which will of course limit its deposition. But more direct evidence for histone eviction of H2B.8 is required.

A previous study (Loyola, Molecular Cell, 2006) in mammalian cells estimated the percentage of nucleosomal histones to be 99%. Therefore, except if this ratio is completely different in seeds, with an important non-nucleosomal histone pool, we expect the large majority of H2B.8 to be chromatin-bound H2B.8. Therefore, the decrease we observe is likely the result of an eviction followed by degradation.

To confirm histone H2B.8 eviction, we carried out ChIP-qPCR in Myc-H2B.8-expressing transgenic lines in dry seeds and seeds imbibed for 48h in the dark to monitor H2B.8 enrichment dynamics during seed imbibition at 5 genes found to be enriched in H2B.8. This analysis indeed confirmed eviction of the histone variant and the data have been included in Figure 4 as panel e.

7. A previous study of H2B.8 in sperm suggests that H2B.8 does not regulate transcription (Buttress et al., 2022), the data in this study demonstrate a quite different conclusion that H2B.8 could regulate the expression of about 921 genes during imbibition. But no clear discussion or explanation could be easily found in the manuscript.

Buttress et al have evaluated gene expression in sperm, in which H2B.8 is naturally enriched. Similarly to our data in dry seed (Figure 3a), the authors do not observe changes in gene expression between WT and mutant in the sperm. Our data suggest that while H2B.8 is not required for gene expression, its eviction might facilitate transcriptional upregulation of a subset of genes. We have discussed this briefly in line 493-497.

Please note that after careful evaluation of our *spike-in* analysis procedure, we revised the bioinformatics spike-in pipeline (see Materials and Methods). While the overall profile is maintained and the overlap between H2B.8-marked and downregulated genes remains significant, this led to a reduction in the differentially regulated genes from 921 to 765 (Figure 4h and i have been modified accordingly). In response to reviewer 4, we have also compared fold changes between H2B.8-marked and H2B.8-free downregulated genes and observed that those genes marked by H2B.8 have stronger negative fold changes compared to the genes not marked by H2B.8 (now new *Supplementary Figure 12e*).

Minor

1. Figure 2C, the western blot quality needs improvement to show an even background.

The background is due to air-bubbles between membrane and plastic film during the development of the blot. We have adjusted the contrast.

2. Figure 2B and 2C is confusing, they should be put together in the same panel.

We thank the reviewer for the suggestion. Panels b and c have now been arranged in the same panel Figure 2b.

3. Could the authors compare the relative association affinity of H2B.4-H2B.4, H2B.4-H2B.8, and H2B.8-H2B.8? This could be another support for the formation of H2B.8 heterotypic nucleosomes.

We thank the reviewer for this suggestion, which brings up an important point that we would like to understand further. We are currently setting up biochemical assays in the laboratory to investigate nucleosome stability. We aim to express all 4 plant histones in *E. coli*, to reconstitute nucleosomes (with either H2B.4 or H2B.8) and to measure their stability by thermal stability assays and single molecule experiments. Since we are just setting up these experiments, it was not possible to obtain results in the time allocated for the revision.

However, to understand better the predicted nucleosome structures, we analyzed *in silico* the two sets of nucleosomes with ChimeraX by determining all residues that are predicted to lie at a distance of up to 4 Angstroms between the two molecules. Results are presented below as alignment of protein sequences between H4-H2B, H2A-H2B or between H2B and DNA, where residues in H2B.4 or H2B.8 that are predicted to make contacts are colored in green, yellow or blue respectively. The number of residues predicted to be in contact slightly varies between a nucleosome with H2B.4 or H2B.8 and can contain amino acids that differ between H2B.4 and H2B.8 (marked by stars). Additional contacts could take place between the longer C-terminal tail of H2B.8 and H2A. Furthermore, contacts may vary between the two variants and DNA. Residues marked in red were previously identified to be involved in H2A-H2B interactions or the H2B-H4 bundle (Luger, Nature, 1997). Positions of predicted α helices are underlined. If the reviewers consider this to be a pertinent addition, these predictions can be added as supplementary data.

H4-H2B bundle

```
H2B.4           MAPKAEKK------------------------------------------------------------
H2B.8           MAPRKPKVVSVTKKKKVVEETIKVTVTEEGDPCVITETANDQETQDLTFSIPVGENVTTV

H2B.4           --PAEKKPASEKPVEEKSKAEKAPAEKKPKAGKKLPK-----------------------
H2B.8           EIPVEVPDERSLPVGENVTTVKIPVDDRDESSPQPPETPVEVRDEPSPQPPETPASKSEG

H2B.4           --------EAGAGGDKKKKMKKK---SVETYKIYIFKVLKQVHPDIGISSKAMGIMNSFI
H2B.8           TLKKTDKVEKKQENKKKKKKKKRDDLAGDEYRRYVYKVMKQVHPDLGITSKAMTVVNMFM

H2B.4           NDIFEKLAQEASKLARYNKKPTITSREIQTAVRLVLPGELAKHAVSEGTKAVTKFTSS--
H2B.8           GDMFERIAQEAARLSDYTKRRTLSSREIEAAVRLVLPGELSRHAVAEGSKAVSNFVGYDSRKR
                                 *            *
```

H2A-H2B interaction

```
H2B.4           MAPKAEKK------------------------------------------------------------
H2B.8           MAPRKPKVVSVTKKKKVVEETIKVTVTEEGDPCVITETANDQETQDLTFSIPVGENVTTV

H2B.4           --PAEKKPASEKPVEEKSKAEKAPAEKKPKAGKKLPK-----------------------
H2B.8           EIPVEVPDERSLPVGENVTTVKIPVDDRDESSPQPPETPVEVRDEPSPQPPETPASKSEG

H2B.4           --------EAGAGGDKKKKMKKK---SVETYKIYIFKVLKQVHPDIGISSKAMGIMNSFI
H2B.8           TLKKTDKVEKKQENKKKKKKKKRDDLAGDEYRRYVYKVMKQVHPDLGITSKAMTVVNMFM
                                              *    *   *      *  *     **   *

H2B.4           NDIFEKLAQEASKLARYNKKPTITSREIQTAVRLVLPGELAKHAVSEGTKAVTKFTSS--
H2B.8           GDMFERIAQEAARLSDYTKRRTLSSREIEAAVRLVLPGELSRHAVAEGSKAVSNFVGYDSRKR
                  *   *    *         * **      *                   *    *  ***  *
```

H2B interaction with DNA

```
H2B.4           MAPKAEKK----------------------------------------------------
H2B.8           MAPRKPKVVSVTKKKKVVEETIKVTVTEEGDPCVITETANDQETQDLTFSIPVGENVTTV

H2B.4           --PAEKKPASEKPVEEKSKAEKAPAEKKPKAGKKLPK-----------------------
H2B.8           EIPVEVPDERSLPVGENVTTVKIPVDDRDESSPQPPETPVEVRDEPSPQPPETPASKSEG

H2B.4           --------EAGAGGDKKKMKKK---SVETYKIYIFKVLKQVHPDIGISSKAMGIMNSFI
H2B.8           TLKKTDKVEKKQENKKKKKKKRDDLAGDEYRRYVYKVMKQVHPDLGITSKAMTVVNMFM
                                   *  **        *  *            *

H2B.4           NDIFEKLAQEASKLARYNKKPTITSREIQTAVRLVLPGELAKHAVSEGTKAVTKFTSS--
H2B.8           GDMFERIAQEAARLSDYTKRRTLSSREIEAAVRLVLPGELSRHAVAEGSKAVSNFVGYDSRKR
                                   **
```

4. Molecular weight markers are missing in several WB results, such as Figure 4D.

Molecular weight markers are now indicated.

5. In Figures 1F–G. Please, specify in the Materials and Methods how “H2B.8-marked” and “H2B.8-free” genes were defined, including any thresholds applied.

This information had been added: “All genes for which the gene body overlaps with at least one nucleotide corresponding to a H2B.8 peak were considered H2B.8 marked. All other genes were considered H2B.8 free.”

6. Figure 4A: H2B.4 is used in the AlphaFold nucleosome model, whereas the RT-qPCR uses H2B.2. Why?

We have redone the RT-qPCR experiments in Figure 4a to show expression of *H2B.4*. We thank the reviewer for the comment, this is indeed more coherent with the GUS lines presented in Figure 4c.

7. Line 253, the claim that global chromatin accessibility may require ATAC-seq or MNase-seq in addition to agarose gels.

We have toned down the statement but kept the figure as it was requested by reviewer 4.

8. Figure 5A: The H3 loading control varies across lanes. Please optimize the blot.

We have carried out an additional independent biological replicate and replaced the blot. The former blot is still present in the “*Unprocessed Western Blots and images*” file in the Source data folder.

9. Figure 6E, middle panel, “hypocotyl”, not “hypocot”?

This has been corrected.

Reviewer #2

The manuscript by Simon et al. entitled "Replication-independent eviction of H2B.8 reveals chromatin reprogramming during seed imbibition" identifies and addresses the role of the histone variant H2B.8 in the seed-to-seedling transition. The histone variant H2B.8 has previously been connected to sperm cell compaction in flowering plants (Buttress et al. 2022). In this manuscript, the authors show that H2B.8 is expressed during seed maturation, contributes to chromatin organization in dry seeds and is transcriptionally repressed and removed during seed imbibition. Under lab conditions, the described presence and dynamics of H2B.8 does not seem to affect seed germination. Using ChIP-seq, RNA-seq and Hi-C of dry seeds, the authors show that H2B.8 marks inactive genes and chromosome-arm TEs and contributes to spatial contacts of specific euchromatic domains. While the absence of H2B.8 (in *h2b.8* mutant) has almost no impact on gene transcripts in dry seeds, it contributes to the transcriptional activation of a subset of H2B.8-marked genes during seed imbibition. Using promoter swaps, the authors demonstrate that H2B.8 can be deposited by general histone chaperone systems and that the histone eviction during seed imbibition is not exclusive to H2B.8 but involves also H3.3. Finally, using different chemical treatments, the authors show that H2B.8 is evicted in a manner that is independent on DNA replication but involves proteasome degradation and it promoted by transcription and protein translation.

This is a well written manuscript, summarizing carefully designed, conducted and critically interpreted experiments. Given the absence of physiological phenotypes, the role of H2B.8 in the seed and during seedling emergence is not entirely clear and the phenotypes are mostly correlative. Nevertheless, the study is in my opinion of high value, newly identifying the involvement of H2B.8 in seed development, providing novel insights into histone dynamics during the seed-to-seedling transition.

We thank the reviewer for the positive evaluation of our work.

I have several comments and questions to the authors:

1. It is not clear whether H2B.8 contributes to general chromatin structure in the seed and/or may have a regulatory role. While some results indeed point to a repressive role (e.g. Figure 1I), possible regulatory role is suggested by selective targeting to a subset of genes (Fig 1E, Fig.S2); progressive activation of H2B.8-marked genes (Fig. 4F) and downregulation of H2B.8-marked genes in *h2b.8* (Fig. 4G).

What genes are affected, and may this support a regulatory role of H2B.8?

We thank the reviewer for this suggestion. We analyzed the GO terms associated with H2B.8-marked up- and down regulated genes. Downregulated genes are enriched among genes linked to stress and defenses response as well as hypoxia and low-oxygen response. We added this analysis to Supplementary Figure 12 as panel f. Oxygen is a key regulator of seed germination. Here the reduced expression of genes annotated for responses to hypoxia and response to oxygen may indicate an altered activation (a reduced perception of oxygen limitation) of the low-oxygen or redox-related transcriptional programs during early germination. Since germination rate and timing are unchanged in *h2b.8* mutants, it could reflect a physiological state change, and not a developmental one.

2. The authors elegantly use tools that prevent the depletion of H2B.8 upon imbibition (e.g. proteasome inhibitors – Fig. S13) or even promote re-establishment of H2B.8 after germination (pHTR5::H2B.8 – Fig. 5A). What is the impact of these manipulations on plants and H2B.8-marked gene expression?

We thank the reviewer for this question as this points to the epigenetic plasticity observed in Arabidopsis plants. Plant ectopically expressing H2B.8 under the H3.3 promoter (this study) or under the viral 35S promoter (Buttress et al. 2022) show indeed no obvious developmental alterations. In (Buttress et al. 2022), the authors even challenged these plants in salt stress conditions but did not observe alterations in phenotypes or gene expression. We have discussed this in lines 504-507.

A possibility is that plants can manage excessive H2B.8 levels by removing it from genes e.g. during transcription as suggested in Buttress et al. in a context where other H2B variants are available. We have set up experiments in the laboratory that aim to create a plant line that is expressing H2B.8 (under a ubiquitous promoter) as only (or main) source of H2B histones by deleting all other H2Bs via CRISPR. This is however a very long-term and tedious task, and we will not be able to provide any results in the time granted for the revision.

Unfortunately, we could not carry out RT-qPCR in the seeds treated with inhibitors, as we had to make a hole in each individual seed with a needle to ensure entry of the inhibitors.

We have however attempted an RT-qPCR experiment in emerging seedlings ectopically expressing (EE) H2B.8 under the *H3.3* promoter at 48hD48hL, when H2B.8 levels increase again in this line (Figure 5a). We chose 6 H2B.8 target genes, which are up (*At2g44460, At5g26220, red*) or down (*At5g24110, At4g21850, blue*), or for which transcript levels are unchanged (*At3g04190, At5g03090, grey*) in *h2b.8-3* mutants compared to WT at 24hD.

We enclose the data here for the reviewer. We could not detect any significant change in expression for these genes at 48hD48hL.

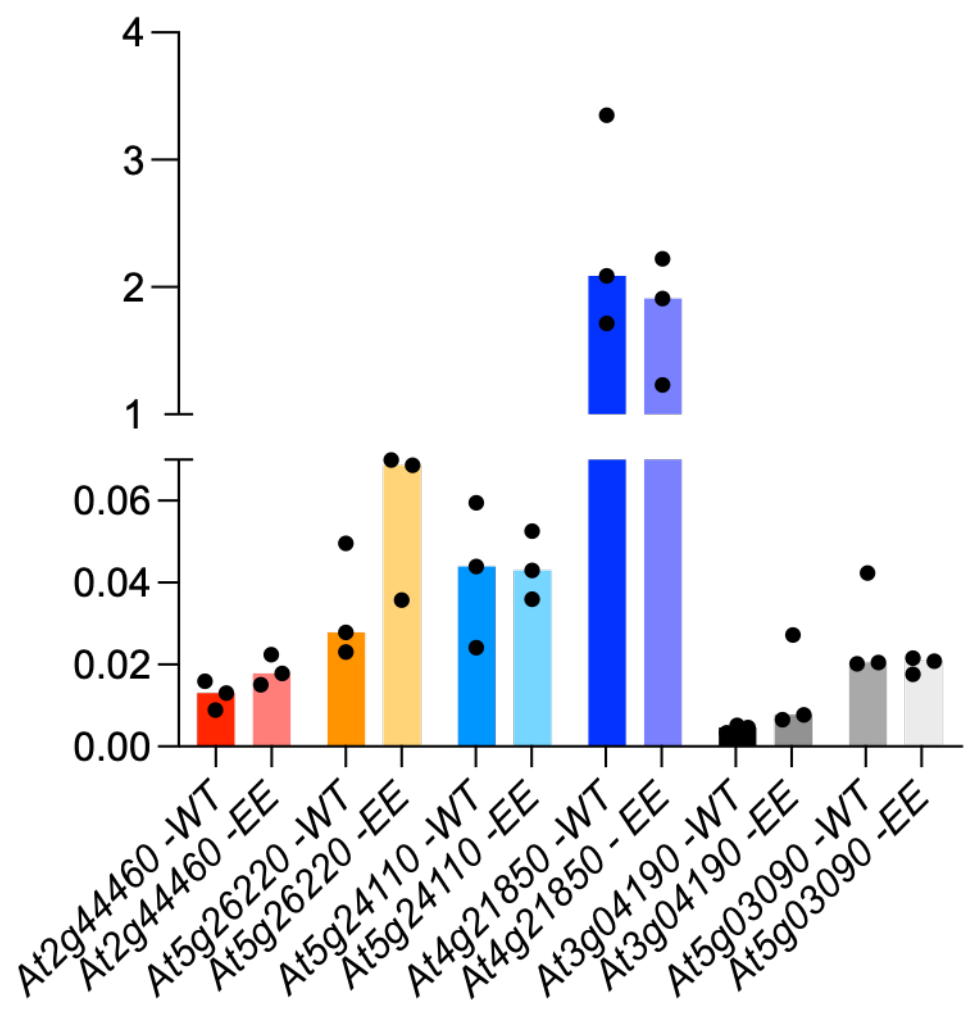

3. Lines 237 – 255, Figures 3 and S6: The volume of the nucleus is moderately increased in *h2b.8* and *hira*, and the phenotype is additive in the combined mutant. The volume of intensely stained regions is increased in *h2b.8* and the combined mutant but reduced in *hira*. The impact of *h2b.8* is explained as being dominant over *hira* (Lines 254 – 255). Higher volume of intensely stained regions may indicate higher tendency to form the compact regions but also their loosening (incomplete/disabled compaction), i.e. two seemingly opposite phenomena. Hence, one could also interpret the results a rescue of *hira* by h2b.8. Can the authors clarify the interpretation of the phenotype?

We thank the reviewer for raising this. Indeed, at this point of the analysis, it is difficult to unambiguously conclude whether heterochromatin (HC) is loosening in the mutant contexts. However, we also scored the number of chromocenters in the 4 genotypes and found a tendency for higher chromocenter numbers in the absence of H2B.8 (in both WT and *hira* background), which would argue that more chromocenters are detectable rather than that the chromocenters are loosened and therefore larger.

Our interpretation of the data is that HIRA and H2B.8 act independently. Our Hi-C data show that H2B.8 leads to clustering of euchromatic (EC) regions, therefore condensing euchromatin. As a result, in presence of H2B.8, we suggest that it is more difficult to segment the AT-rich and Hoechst-bright heterochromatic regions from the remaining condensed euchromatin. Upon H2B.8 depletion, these euchromatic regions decondense moderately (Figure 3f) and the nuclear volume slightly increases, and it is therefore easier to segment the brighter heterochromatin from the euchromatin. This could happen independently of the genetic background (WT or *hira*). The presence of H2B.8 could also modify H1 and/or H2A.W occupancy genome-wide, which could impact chromatin condensation.

In *hira* mutants, nucleosome occupancy is reduced (Layat, 2021), which could influence chromatin organization, preventing heterochromatic regions to be detected easily. We could speculate that reduced nucleosome occupancy leads to a reduction in or redistribution of other variants including H2A.W or H2B.8 (while the global H2B.8 / H3 ratios are not significantly altered, see Figure 5b), which ultimately affects chromatin organization rendering it even more difficult to segment heterochromatin from the condensed heterochromatin in the *hira* mutant background. Similar to the WT situation, loss of H2B.8 and the global euchromatin de-condensation with slighter larger nuclei could facilitate segmentation of heterochromatic regions.

To confirm the differences in chromatin organization between the 4 genotypes, we also analyzed another parameter, not only based on volumes, but also on the intensity ratios of the segmented Hoechst-bright regions and the whole nucleus, which is the Intensity RHF (RHF = relative heterochromatin fraction, defined as pixel intensity within the segmented Hoechst-bright regions and the total volume). This parameter considers the subtle nuclear volume changes. The Intensity RHF confirms differences in chromatin organization in the *h2b.8* mutants. We further find that the mean values for the double mutant lay in between the single *h2b.8* mutant and *hira*, which is consistent with our interpretation. The results for the intensity RHF are now added to Supplementary Figure 6.

4. The level of H2B.8 marking positively correlates with local chromatin contacts (Fig. 3F). Is this reflected in differential activation of the underlying genes upon imbibition/germination?

This is a very interesting question and something that we had also expected. Indeed, we did observe shifts from A to B and B to A compartments when comparing WT and *h2b.8* dry seed embryo nuclei, however we could not establish any significant correlation between compartment shifts and differentially regulated genes at 24h imbibition.

We will prepare a follow-up study, that will include Hi-C analyses in germinating seeds and in which we hope to link the organization in dry seeds to future transcriptional regulation.

Other comments:

5. Line 97 – "Besides sperm nuclei…" – instead of "Besides in sperm nuclei…"

Thanks for spotting this mistake. It has been corrected.

6. Line 139 – "strongly visible?" instead of "strongly enriched"? Enrichment suggests comparative amount in relation to something else….

Corrected.

7. Line 129 – Figure 1a – H2B.8 seems depleted form the SAM (compared to its level in other tissues and to H3.3) – is this so and can the authors comment on this?

Indeed, this is something we also noticed. To confirm this, we imaged additional H2B.8-GFP expressing nuclei and in none of them, we could observe staining in the SAM. We modified the statement at the beginning of the results chapter. It now reads: "*Like H3.3-GFP, H2B.8-GFP is strongly visible in most of the small nuclei of the dry seed embryos, except in the shoot apical meristem (Figure 1a).*"

8. Line 139 - Figure S1A and B: the localization of H2B.8 seems to be peripheral using the Myc-H2B.8 (A) but more centrally located in the H2B.8-Flag line (B). Do the differently tagged H2B.8 give different distribution pattern and why? Can the authors comment on this?

Here we detected tagged H2B.8 with two different antibodies with different affinities which might explain the differences in the chose images. We have now provided an additional set of nuclei in Supplementary Figure 1A -B for comparison.

Confocal images of immuno-stained nuclei have been added to the image datasets on Omero.

9. Lines 150-161 – There seem to be some discrepancies in the numbers of genes targeted by H2B.8: Text (line 150) states 7749 genes (same in Fig S2c,e and Fig 1e lower venn) but Fig. 1E upper venn depicts 8179 genes, and legend to Fig. 1f,g states 7549 genes). Can you explain/correct this?

We sincerely thank the reviewers for spotting these discrepancies that escaped our attention. We have verified one more time all numbers and corrected them.

The discrepancy between 7749 genes marked with H2B.8 and the 7549 genes came from the different annotations we and the authors of the Narsai 2017 paper used for the data analyses (200 genes in our dataset are not present in the Narsai gene list). We have indicated now in the legends where these numbers differ.

10. Figure 1f, g – It is not entirely clear from the figures of the legends what is depicted: is it all annotated genes separated by being/or not marked by H2B.8 OR H3K27me3/H2A.Z targets separated by being or not marked by H2B.8? The logic suggest it is the former/first option, but it should be made clear by the legend, including stating the number of genes that are NOT H2B.8 targets.

It is the first option: Figure 1f and g depict H3K27me3 and H2A.Z enrichment of all (7749) genes that are H2B.8-marked and a random group of 7749 genes not marked by H2B.8. We have now stated this clearly in the legend.

11. Figure 1f – the enrichment profile lines are relatively thin and poorly visible.

We have improved this.

12. Line 1072 (Legend and Fig 2b,c) – (i) it is not clear what input 1/5 or 1/10 means, (ii) it would ease reading the figure if the antibodies used for detection were specified in the figure and (iii) formulation: "Revelation with anti-Myc…reveals" – instead "Detection using anti-Myc or other noun?

We have explained the dilution of the input to 1/5th or 1/10th in the legend. We now indicated the antibody used in this figure and have homogenized this over all other figures. The legend was also corrected as suggested by the reviewer.

13. Line 314 – 315: "Given the substantial co-enrichment of H2B.8 and H3K27me3…." – the statement refers to Fig. 1E? Please clarify/provide reference.

Yes, this refers to Figure 1e and f. We added a reference to these Figure panels.

14. Line 327 should refer to Fig S12C instead of S13C and the figure reference would be better suited after the previous sentence on line 326. For clarity, Figure 4G would be better referred to upon first mentioning of the numbers of genes changed (line 327).

Thanks for noticing this error. We have replaced S13c with S12c and refer to Figure 4g (now Figure 4h) at the same time.

15. Line 388: "…exposed imbibed seeds TO continuous light"

We have corrected this.

16. Line 388: Evidence/reference to figure is missing to support the statement that "….,25 – 57% of the seeds germinated after 48h across 3 biological replicates."

We have added a small table to the supplementary Figure, now Figure S14a.

17. Line 523: "….C-TERMINAL lysine"

Thanks for spotting this. This has been corrected.

18. Line 1189 – legend to Fig S2: "H3K4me3-marked genes"

Thanks for spotting this. This has been corrected.

Reviewer #3

I co-reviewed this manuscript with one of the reviewers who provided the listed reports. This is part of the Nature Communications initiative to facilitate training in peer review and to provide appropriate recognition for Early Career Researchers who co-review manuscripts.

Reviewer #4

There are two main messages in this manuscript by Simon et al. The first one is focused on analyzing the nature and functions of the H2B.8 variant, which has a longer amino terminal domain as well as some amino acid changes and is abundant in seeds, both in dry seeds and during the seed-to-seedling transition. The other part of the manuscript investigates the chromatin reprogramming during the seed-to-seedling in Arabidopsis.

I will start highlighting the strengths of the manuscript: the experimental part has been thoroughly designed with a clear interest in detail and overall presenting an array of well performed experiments (see below for more specific comments that need to be addressed). The authors profile the seed-enriched histone variant H2B.8 using tagged lines, a custom antibody, ChIP-seq, Hi-C, imaging/segmentation, RNA-seq (with spike-in at 24hD), and S-phase timing by EdU/FACS. The manuscript is in general well written, with only a few details that will benefit from some polishing (see below in minor comments). The original ideas, understanding the role of H2B.8 in seeds and the seed-to-seedling nuclear reprogramming, and their crosstalk, are attractive and will be of interest not only for the plant community, but also for a broader scientific community focus on understanding how transcriptional changes linked to development are governed. Despite these undeniable strengths, the manuscript deals with an important caveat: the lack of functional results for H2B.8 that prevents the manuscript from driving final conclusions and answering the following question: why do Arabidopsis seeds need to accumulate this histone variant? The authors showed that H2B.8 accumulates in dry embryos, forms heterotypic nucleosomes (I find this part and the biochemical analysis of the H2B.8 variant particularly compelling!), maps to lowly expressed genes and euchromatic TEs (often co-marked by gene-body H2A.Z and H3K27me3), and that H2B.8-rich regions cluster in 3D. Upon seed imbibition, H2B.8 transcripts drop and the protein disappears from the nuclei in a tissue-ordered manner and at 24hD, a subset of H2B.8-marked genes fails to activate in *h2b.8*. Still, all these results are descriptive and insufficient to address that initial question for the need to accumulate this variant in seeds. On the other hand, the results allow them to propose a model where water uptake triggers replication-independent histone turnover that reconfigures 3D architecture to enable early gene activation.

Here some more specific details that I would like to discuss with the authors and that, whether addressed, may help to improve the manuscript:

We thank the reviewer for the insightful comments.

Point 1:

Why do the authors describe the reduction of H2B.8 from the chromatin as eviction? I don't think that they present data to check whether the protein is evicted from the chromatin during the transition from seed to seedling. However, they present robust data that shows that 1) the *H2B.8* transcript amount decreases during germination (even showing an explanation for this decline due to H3K27me3 high signal on the *H2B.8* locus) and 2) the H2B.8 protein levels decline most probably degraded by the proteasome, which may be key for the histone turnover during this developmental transition. To check for eviction, so removal from the chromatin, the authors would have needed to separate the pool of H2B.8 associated to the chromatin from the free pool, something that they didn't do and I don't think they need to check because they have enough results to understand why H2B.8 levels decline during germination. Please, reconsider the wording avoiding the term "eviction". In addition, considering that protein degradation seems to have an important effect on H2B.8, I would strongly recommend moving supplementary figure 13A-B into main figure 6. Indeed, a possibility that cannot be discarded is that protein synthesis/transcriptional onset is indeed needed for synthesis of the protein degradation machinery to allow H2B.8 degradation.

We thank the reviewer for these suggestions.

The point of H2B.8 eviction has also been raised by Reviewer 1. As pointed out above, we expect from the literature from mammalian cells (Loyola, Molecular Cell, 2006) that the large majority (99%) of all cellular histones are nucleosomal histones. Therefore, except if this ratio is completely different in seeds, with an important non-nucleosomal histone pool, we expect the large majority of H2B.8 to be chromatin-bound H2B.8. Therefore, the decrease we observe is likely the result of an eviction followed by degradation.

To confirm histone H2B.8 eviction, we carried out ChIP-qPCR in Myc-H2B.8-expressing transgenic lines in dry seeds and seeds imbibed for 48h in the dark to monitor H2B.8 enrichment dynamics during seed imbibition at 5 genes with H2B.8 peaks in dry seeds. This analysis indeed confirmed eviction of the histone variant and the data have been included in Figure 4 as panel e.

We have moved the MG132 treatment to the main Figure 6 as suggested by the reviewer.

Point 2:

My next comment is related to the previous one. I think that the authors had the hypothesis that histone eviction mediated by histone chaperones played a major role in H2B.8 abundance. I do agree that their results with the *ssrp1-2* mutant partially supports this idea (there is only a partial reduction in H2B.8 levels). Therefore, this could indicate that FACT is important for its deposition on the chromatin, but still, it does not explain how the decline in its levels occur during germination. I also think that the authors focused too much on the idea of eviction/turnover and miss a clear opportunity to understand the proteolytic regulation of H2B.8. Furthermore, what happens with the Flag-H3.3 binding to the chromatin during imbibition? I agree that its amount slightly decrease probably due to the H2B.8 promoter being repressed, but it doesn't seem to decline as much as H2B.8 protein levels. Therefore, I would expect that the Flag-H3.3 protein can still be detected during imbibition by ChIP-PCR to fully understand how much de novo deposition occurs. This would indicate that there is another factor in the equation: protein degradation that requires the H2B.8 protein and doesn't occur efficiently in the Flag-H3.3 line.

Our preliminary ChIP-qPCR experiments with the pH2B.8::Flag-H3.3 line indicate that H3.3 deposited during maturation is indeed evicted at some loci, while we also noticed enrichment at 1-2 loci after imbibition suggesting that some of the evicted H3.3 histone variants could also be recycled in cis and in trans. We would like to follow up these observations in a different manuscript, as it merits deeper analysis using ChIP-seq.

Point 3:

3.1 The argument that "eviction" precedes DNA replication is very interesting but requires stronger quantitative backing. In Fig. 6A, please provide replicate counts, gating strategy, coefficients of variation for the 2C peak, and quantitative cell-cycle parameters (e.g., %G1, %S, %G2 ± CI) at each timepoint.

We have now provided an example of the gating (Supplementary Figure 13a) in dry and germinating seeds and added quantitative values of the 2 independent replicates we conducted into supplementary Figure 13b.

Clarify how endoreduplication is distinguished from mitotic S-phase in this context.

In this context we cannot distinguish replication from endoreduplication. While this is an interesting question, it would, in our understanding, not influence our conclusion that H2B.8 loss is due to dilution of parental histones (and incorporation of new histones, including other H2B variants) that would occur during both replication and endoreduplication.

3.2 For Fig. 6B, report the % of EdU nuclei per tissue per timepoint, with embryo and nucleus counts and appropriate statistical tests. Together, these data would make the timing of S-phase onset explicit.

We have now quantified the EdU positive nuclei at each time point. Data have been added to Supplementary Figure 13 as panel c.

3.3 Finally, authors could include a one-sentence conclusion in the Results stating clearly that the decline in H2B.8 (≤24hD) occurs before the first detectable EdU^+ nuclei (48hD24hL), thereby preceding DNA replication.

We thank the reviewer for this suggestion and included a sentence.

3.5 Still related with H2B.8 "eviction", in figure 4 several results are only partially quantified. In panel A, transcript dynamics are shown, and a Friedman's test is reported ($p<0.05$), but the number of biological replicates (n), the definition of error bars (SD vs. SEM), and exact p-values are not provided.

We have now added this information to the legend of Figure 4.

3.6 In panel D, the quantification suggests four biological replicates, but only three uncropped blots are shown in the supplementary, replicate numbers per condition are inconsistent (3–4 dots), and the stability of the H3 loading control is not addressed. Furthermore, only normalized values are presented, without effect sizes or confidence intervals to define the magnitude and precision of the ~50% reduction at 24hD.

We are sorry, there seem to be indeed a discrepancy between the legend, which stated 4 and the graph that included only 3 biological replicates. We have now redone a 4th biological replicate, which was included in the graph and replaced the blots shown in panel d with the 4th biological replicate with a more homogenous H3 loading.

Details of the statistical analyses conducted in the manuscript can now be found in a new “Statistical analyses” file in the Source data folder.

3.6 In panel E, GFP imaging is descriptive, lacking per-nucleus quantification, embryo/nucleus counts, and statistical testing. Finally, the term “rapid” is used, but not defined quantitatively (for example “≥50% reduction in cotyledon nuclei by 24hD”). Providing per-tissue quantification across time points, with exact replicate numbers, effect sizes, confidence intervals, and explicit thresholds for “rapid eviction”, will be essential.

We have now determined the number of GFP-positive nuclei using a randomized double blind set up. The data are now presented as positive nuclei per μm^2 tissue in Supplementary Figure 11c, further details of the statistical analysis including effect size and confidence intervals can be found in the “Statistical analyses” file in the Source data folder.

We have removed the term ‘rapid’ from the manuscript and replaced it with more precise time information.

Point 4:

It is indeed very unfortunate that neither the *h2b.8* mutant nor its transgenic lines (overexpressing line or under the H3.3 promoter) showed any obvious phenotypic changes under the analyzed conditions. However, I think that the authors could have checked a few more things. For instance, in the discussion they commented that “deficient germination or reduced seed longevity might only be observed in nature”. However, this can be easily mimicked and analyzed in the lab to check if seed longevity is affected in the *h2b.8* mutant or the overexpressing lines. They also mentioned that “when seeds are imbibed under suboptimal conditions and enter secondary dormancy, H2B.8 expression is induced”, did they check dormancy in the mutant or overexpressing lines? In addition, repeated imbibition – desiccation, or suboptimal germination conditions (in the presence of GA inhibitors or chemicals) could have been easily tested.

Indeed, we agree with the reviewer that this lack of phenotype is quite disconcerting – we have tried hard to find a germination phenotype, but we have been failing to find anything reproducible and convincing under laboratory conditions. We have now performed a seed aging assay as well as one type of seed secondary dormancy test. These data are now included in Supplementary Figure 4 as new panels e and f. We have modified the results section and the discussion accordingly. We have also tested GA inhibitors however with non-

conclusive results as we observed variations in the seed responses among seed lots. For ABA treatments we did not observe any difference between the WT and the *h2b.8-3* mutants seeds:

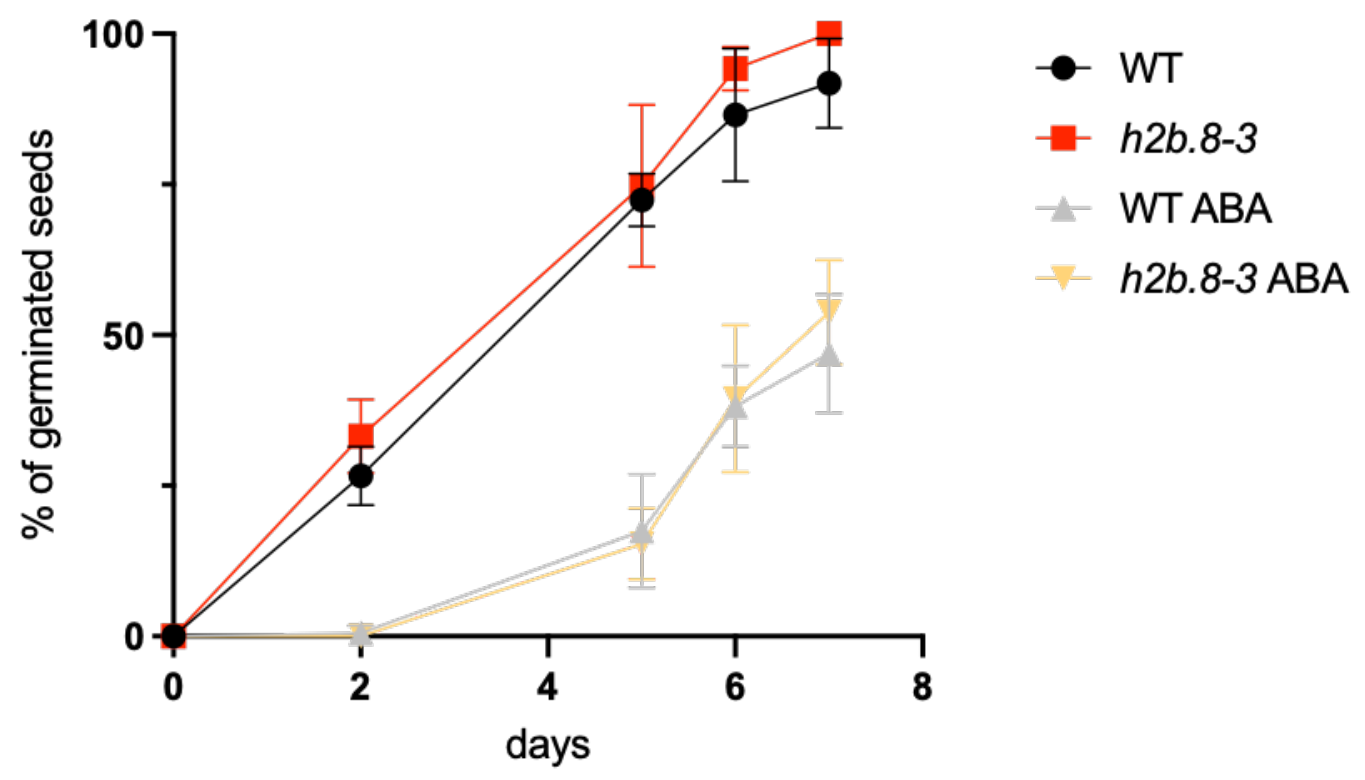

Further experiments to induce secondary dormancy by different means and further inhibitor experiments will be conducted in the future.

As an argument for the lack of obvious phenotype they discussed about "The absence of a germination phenotype is consistent with the *h2b.8* mutants being fertile, and the specific phenotypic differences observed for the loss of other histone variants under standard growth conditions such as the sperm-specific H3.10 or H3.14 and H3.15, which are induced in roots upon stress or wounding". However, as far as I know, when mutants affected in these variants are grown under specific conditions, there are observable phenotypic changes.

To our knowledge, no fertility phenotype was observed for *h3.10* mutant plants nor growth defects for the *h3.15* mutant plants (they observed impact on callus growth in the over-expressing lines). Plants lacking H3.14 are also phenotypically identical to wildtype under standard growth conditions but show enhanced root growth under NaCl.

We have modified the sentence. It now reads: "The absence of a germination phenotype is consistent with the *h2b.8* mutants being fertile, and the absence of altered phenotypes for mutants in other histone variants such as the sperm-specific H3.10 [24], H3.14 [56] or H3.15 [57] under standard growth conditions."

Therefore, this will indeed indicate that the authors are missing the right conditions to observe the *h2b.8*-related phenotype that would be crucial to understand its molecular functions.

We remain convinced that there is a specific function for the H2B.8 variant that allowed its evolutionary conservation and expression in seeds– but which might be difficult to visualize under laboratory conditions or is rather linked to different growth conditions of the mother plant, an aspect that we have not yet explored. Phenotypes might be also less easy to spot in Arabidopsis, which shows extraordinary epigenetic plasticity. We are now in the process of mutating the two *H2B.8* orthologs in rapeseed, which, given the bigger and allopolyploid genome, might reveal novel functions for H2B.8.

Point 5:

5.1 The RNA-seq analysis shows that H2B.8-marked genes tend to activate upon imbibition and that their induction is impaired in *h2b.8* mutants.

However, the time course currently comes from an external dataset, while the genotype effect is presented only at 24hD. To strengthen the narrative under your own growth conditions, please include a short RT-qPCR panel across DS → 24hD → 48hD (±48hD24hL) for some H2B.8 target genes.

We have performed RT-qPCR across time points during the seed-to-seedling transition for 4 H2B.8 targets, two genes (*At2g44460, At5g26220*), which are up- and two genes (*At5g24110, At4g21850*), which are downregulated in *h2b.8-3* mutants compared to WT at 24hD. We added these data as Supplementary Figure S12c.

5.2 In addition, to establish that the overlap in Fig. 4G–H is not incidental, provide enrichment statistics (odds ratio with 95% CI, Fisher's exact p) and a distribution-level test (log2FC shift, Wilcoxon). This will demonstrate that the overlap between down-regulated genes and H2B.8-marked genes is more than expected by chance.

We thank the reviewer for these suggestions. We have performed a Fisher's exact test, which shows statistically significant enrichment in H2B.8-marked genes among the downregulated genes. This information has been added to this Figure panel, which is now Figure 4h.

We have further carried out a Wilcoxon test as suggested by the reviewer to compare the fold changes of H2B.8-marked and H2B.8-free downregulated genes revealing that H2B.8-marked genes have a stronger negative fold change compared to H2B.8-free genes. These new data have now been added as Supplementary Figure 12e.

5.3 It is interesting that despite the impairment of that set of genes during germination, the mutant still germinates well. Can the authors analyze GO terms for both H2B.8 target genes and H2B.8 mis-regulated target genes? This may provide some clues about its functional role and perhaps the most appropriated conditions to further test for phenotype.

We have carried out GO analysis of H2B.8-marked down- and upregulated genes (now in Supplementary Figure 12f). Downregulated genes are involved in stress and defense as well as hypoxia and low-oxygen response. This observation is interesting as oxygen is a key regulator of seed germination. Reduced expression of genes annotated for responses to hypoxia and response to oxygen may indicate an altered activation (a reduced perception of oxygen limitation) of the low-oxygen or redox-related transcriptional programs during early germination. Since germination rate and timing are unchanged in *h2b.8* mutants, it could reflect a physiological state change, and not a developmental one.

This might be indeed a clue to set up new experiments in the future for example to test the germination of seeds under low oxygen conditions.

Point 6:

6.1 Figure 6E shows that H2B.8 "eviction" is impaired by CHX and α-amanitin, but the evidence is qualitative. To support the conclusion that this requires *de novo* transcription/translation, please provide per-nucleus retention metrics stratified by tissue and timepoint, with embryo/nucleus counts, effect sizes, confidence intervals, and exact p-values.

We have quantified the number of H2B.8-positive nuclei per μm^2 tissue for the images shown. These data have now been added to a new Supplementary Figure panels S14 c-g. Further information on the statistical analysis can now be found in the "Statistical analyses" file in the Source data folder.

Because both inhibitors can impair general physiology, viability/germination controls, experiments are needed to exclude nonspecific toxicity. Finally, since α-amanitin has specificity and uptake caveats, a second transcription inhibitor would strengthen the claim; if not feasible, this limitation should be noted with justification of dose and conditions.

We thank the reviewer for this remark. In contrast to a previous study, to which we have referred (Rajjou, Plant Physiology, 2004), which used *testa* (*tt2-1*) mutants, we have proceeded by adding a hole with a small needle into each seed so guaranteeing that all substances are properly taken up. To ensure that the seeds remain metabolically active after treatment, we have tested this by 2,3,5 triphenyl tetrazolium chloride (TTC) staining. All seeds, except those treated with Cycloheximide remain metabolically active (new Supplementary Figure 14h). In light of the results that Cycloheximide-treated seeds are no more metabolically active under our conditions, we prefer to remove the statement that translation is required for H2B.8 eviction from the manuscript. For the α-amanitin treatment we have tested another, lower concentration with similar results (*Figure 14g, h*).

6.2 the authors discussed that more *in vitro* studies will be needed to fully understand the impact of H2B.8 on the nucleosome. However, could the Hi-C data help to support any of the two possible scenarios, that H2B.8 positively or negatively affects DNA-octamer interactions?

We thank the reviewer for this suggestion. Unfortunately, we do not see a way how the Hi-C data could help to support either scenario. As discussed in the response to reviewer 2 and to better understand the predicted nucleosome structures (with and without H2B.8), we analyzed *in silico* the two sets of nucleosomes with ChimeraX by determining all residues that are predicted to be at a distance of up to 4 Angstroms between the two molecules (H4-H2B, H2A-H2B or H2B with DNA, see data in response to reviewer 2). The number of residues in contact slightly varies between a nucleosome with H2B.4 or H2B.8 and can contain amino acids that differ between H2B.4 and H2B.8. Additional contacts could take place between the longer C-terminal tail of H2B.8 and H2A. Furthermore, contacts may vary between the two variants and DNA. We believe however that the direct effect on nucleosome stability can only be addressed by further *in vitro* experiments. We are currently setting up those experiments in the laboratory with the aim to test nucleosome stability and DNA unwrapping at a single-molecule level; however, these experiments will take investment and time to be set up.

6.3 Did they try micrococcal nuclease analyses in the WT vs the mutant to see whether access to nucleosomal DNA could be affected? I do agree that for other functional analyses (e.g. use of deletions and point mutations) deeper and time-consuming approaches will be needed.

We have analyzed chromatin accessibility globally by MNase digestion (see Supplementary Figure 7). This very broad analysis did not reveal any differences between WT and mutant. We have toned down our statement, in request of reviewer 1.

Point 7:

-I would strongly recommend unifying the experimental timeline and conditions across the paper. Readers, like this reviewer, will follow the biology more easily with a single, consistent timeline from dry seeds through imbibition and transfer to light. Please add a schematic used consistently in Figs. 4–6: DS → 24hD → 48hD → transfer to light → 48hD24hL → 48hD48hL → 48hD72hL, plus the continuous-light (cL) branch.

Define hL ("hours after transfer to long-day light") and cL ("continuous light") at first use in the main text and repeat in relevant legends.

We thank the reviewer for the suggestions. We have now included schematics to Figure panels 4a, 5a, 6a and 6c and added the definition of hL and cL.

Mark the transfer-to-light boundary directly on plots that span dark→light. Also resolve the inconsistency "48h at 4 °C" (Results) vs "48h at 6 °C" (Methods) and propagate the chosen value throughout text, legends, and Methods.

We have visualized the dark – light boundary on plots in Figure 4 and Supplementary Figure S12b and have corrected the inconsistency between 4 °C and 6 °C.

Other minor comments:

1. Textual edits

• Ensure consistent temperature notation and spacing: 23 °C, 4 °C (number + thin space + °C).

This has been corrected.

• Supplementary Data numbering: "Supplementary Data 6" appears twice—please renumber to avoid duplication.

We thank the reviewer for noticing this mistake. This has been corrected.

• Introduction line 78: "FACILTATES CHROMATIN TRANSCRIPTION (FACT) ensure" → "FACILITATES CHROMATIN TRANSCRIPTION (FACT) ensures."

This has been corrected.

• Fig. 2 text: “b-sheets” → “β-sheets”; “alpha” → “α”. Please, Use standard Greek letters in text (α-amanitin).

This has been corrected.

• Discussion line 524: “may may favor H2Bub enrichment” → “may favor H2Bub enrichment.”

This has been corrected.

• Suppl. Fig. 12 legend: “genes that loose or gain H3K27me3” → “genes that lose or gain H3K27me3.”

This has been corrected.

• Methods line 554 and fig. 6 legend-Imbibition/Inhibitors: “Cycloheximidine” → “Cycloheximide” (match earlier Results spelling).

We thank the reviewer for noticing this spelling mistake. This has been corrected.

• Methods line 709- Chromatin shearing: “chromatin was shared using a Covaris” → “chromatin was sheared using a Covaris.”

We thank the reviewer for noticing this mistake. This has been corrected.

• Discussion lines 473-476: this should be at least mentioned in the results part. As a reader, I thought this information was missing from the results.

A sentence has been added to the results section when describing Figure 5a.

• Discussion lines 475-476: please, add “this finding indicates that, under our standard growing conditions,…”

This has been added.

• Discussion line 460-461: It doesn’t read well. Please, change to “…with the H2A.Z and H3K27me3 co-enrichment in gene body,...'.

This sentence has been modified.

• Results lines 326-328: authors mentioned Figure S13c. Do they mean Figure S12c?

Yes, indeed, we have modified this.

• Results line 323: “…a subset of genes” reads vague. I know that the specific numbers are in the figure, but it would be more informative to add them to the text.

This information has been added.

• Figure 4b: it took me a while to realize, which gene corresponds to H2B.8 as, I think, this is the first time it is mentioned with the code. It would also be nice to indicate the orientation of the genes to understand which intergenic region corresponds to the promoter.

We have modified this figure panel adding triangles to show the direction of the genes as well as the name H2B.8 under the gene code.

• Add a short, first-use definition of “heterotypic nucleosome”.

This has been added to the introduction.

• Maintain consistent unit spacing (e.g., 100 μM, 20 μm, 25 °C) and symbol usage across main and Supplementary.

This has been corrected.

2. Statistics, legend

• For every figure/panel, state exact n (distinguish biological vs technical), define error bars (SD/SEM/95% CI), and report exact p-values with the named test (and any multiple-testing correction).

We have added this information to each legend. In all figures we only present biological replicates. We have now added a “Statistical analyses” file in the Source data folder in which information on the statistical analyses can be found.

• Where summaries are shown, add per-replicate dots and effect sizes with 95% CIs.

Information on the statistics has been added as a “Statistical analyses” file in the Source data folder.

3. Data

• Provide a figure-to-data map (table: panel → raw data files → analysis script/software + version) and link it to the existing OMERO/GEO entries. In Data availability, if it is pointed towards readers to this mapping table as the single place to find all files, it might be very easy and helpful.

We have added the “Figure-to-data map” file in the Source data folder. We also included original values used in boxplots as ”Raw data for boxplots and violin plots” file in the Source data folder.

• Lines 182-185: “Both fusion proteins migrate at a similar molecular weight and slower compared to the H2B.4 variant (Figure S3b), revealing that, in dry seeds, the reduced electrophoretic mobility of H2B.8 is conferred by the tail sequence rather than by post-translational modifications”. Why? Can one fully discard the possibility that well conserved post-translational modifications occur in yeast? Or am I missing something?

We thank the reviewer for this remark that has also been raised by reviewer 1. We have expressed a GST-tagged H2B.8 tail also in bacteria and could show that it migrates at an unexpected molecular weight. These new data have been added to Supplementary Figure 3 as panel C.

We thank the reviewers for the constructive suggestions throughout the reviewing process that has helped us to substantially improve the manuscript.

Please find enclosed the detailed response to the reviewers' comments.

Reviewer #1:

The authors have addressed most of my concerns. The revised manuscript provided substantial evidence, linking the behavior of H2B.8 to epigenetic reprogramming and gene expression during seed imbibition. Again, the manuscript is well-written and most of the conclusions are supported by the data. Revisiting my previous concerns, I still recommend the authors to pay attention to the conclusion that H2B.8 forms heterotypic nucleosomes. After which, I think the conceptual advance of this work merits its acceptance for publication at Nature communications.

Here is my remaining concern:

It is still not convincing enough for me that H2B.8 forms heterotypic nucleosomes. I mean, yes, it could form heterotypic nucleosomes; but is it the only form of nucleosomes that H2B.8 forms? The current evidence did not exclude the possibility that it could form homo. However, in the results part (Lines 195-207), the description gave the impression that it only forms heterotypic nucleosomes, which is very strong. I noticed the IP/input ratio, but have different opinion based on the data in Figure 2b. It looks like for me that the IP/input ratio for H2B is smaller than H3, by eye. In Osakabe et al., 2018, the authors' conclusion is the majority of H2A nucleosomes are homogenic, but they also observed the potential heterogenic forms for H2A-H2A.W and H2A.X-H2A.W as well as H2A.W.7-H2A.W.6. Taking that into account, I think a more inclusive conclusion would be more acceptable. For experiments that I previously requested, to avoid heavy chain interference, the authors may consider using anti-Myc antibody from a different species than that used for generation of anti-H2B.8 for IP or they may want to use a secondary antibody that only recognizes the light chain.

Otherwise, the authors should try to avoid such strong claim in Abstract, sub-titles, and Figure titles.

We thank the reviewer for the constructive comments. We had tried to use the anti-Myc antibody from a different species but still observed some limited cross-reactivity and could therefore not find a clear answer whether homotypic nucleosomes also exist for H2B.8.

In response to the reviewer's suggestion, we have toned down the conclusions in the abstract, sub-title, results and Figure title.

Others:
Line 45-46, Abstract, I believe this sentence is grammatically incorrect.

Thanks for noticing, indeed an editing error slipped through our attention. This has been corrected.

Reviewer #2 (Remarks to the Author):

This is a revised version of a manuscript that I had reviewed before. I have read through the manuscript and confirm that all my comments have been addressed to my satisfaction and to what I think is within the scope of this work.

Reviewer #3 (Remarks to the Author):

I co-reviewed this manuscript with one of the reviewers who provided the listed reports. This is part of the Nature Communications initiative to facilitate training in peer review and to provide appropriate recognition for Early Career Researchers who co-review manuscripts.

Reviewer #4 (Remarks to the Author):

I would like to thank the authors for their efforts to reply to my and other reviewers' concerns. I can confirm that they have carefully considered all reviewers' comments to provide an improved version of their manuscript:

• One of my main concerns regarding the claim of H2B.8 eviction is now better supported by ChIP-qPCR experiments of selected candidate genes showing loss of chromatin-associated H2B.8 during imbibition.

• The replication-independence claim is now backed with quantified FACS/EdU support in Supplementary Figure, and the text explicitly states that H2B.8 decline precedes the first detectable EdU-positive nuclei.

• The inhibitor experiments are no longer just qualitative: they now quantify retention under MG132 and α-amanitin, add TTC viability controls, and they have removed the stronger cycloheximide-based translation claim once viability failed.

• They also strengthened the transcriptional part by adding enrichment/GO support for the H2B.8-marked downregulated genes.

• I still consider that the lack of phenotype of the mutant and transgenic lines makes very difficult to drive final conclusions, but again this is not due to the lack of efforts from the authors. Future experiments as the one they mention (functional analyses in other species such as *B. napus* and more physiological analyses such as the response to hypoxia) may help to set some light. However, I do consider that this caveat still needs to be clearly stated and discussed in the manuscript.

We now clearly state this limitation in the discussion.

• Although the H3.3 / global chromatin reprogramming angle is improved, I still feel that this part is less mechanistically developed than the H2B.8 story. For example, the manuscript shows that seed-deposited Flag-H3.3 declines during imbibition and that total H3 occupancy is maintained at tested loci, but it still does not directly resolve how much of this reflects true H3.3 eviction versus recycling/redeposition. In their response, the authors indicate that deeper analysis of H3.3 dynamics will be part of future work. I therefore think that the broader conclusions regarding H3.3/global chromatin **reprogramming should be toned down further** so that they remain fully aligned with the data currently shown.

We have modified the results part describing Figure 5c and d as well as the corresponding discussion, from line 922 on, to incorporate the notion that H3.3 can be also recycled and redeposited.

• There are still a few editorial/polish issues. For example, the abstract still contains the wording error "thereby initiating and therefore initiates." I would also recommend one more careful language edit throughout, as there are still a few sentences that read as slightly overcomplicated or repetitive.

Thanks for noticing, indeed an editing error slipped through our attention. This has been corrected.

We ran the manuscript through DeepL to identify grammar, spelling, and orthographic errors, and we made two changes to the figure arrangement to improve readability.
