# Supplementary material for: Replication-independent eviction of the histone variant H2B.8 reveals chromatin reprogramming during seed imbibition
Source: Nat Commun. 2026 Jun 29;17:8102. doi: 10.1038/s41467-026-74230-6 (PMC13458839; doi:10.1038/s41467-026-74230-6)
Supplement: Supplementary file 4 [file 41467_2026_74230_MOESM10_ESM.zip › Source_Data/Unprocessed Western Blots and images.pdf]

Figure 2a

Control of H2B.8 antibody specificity

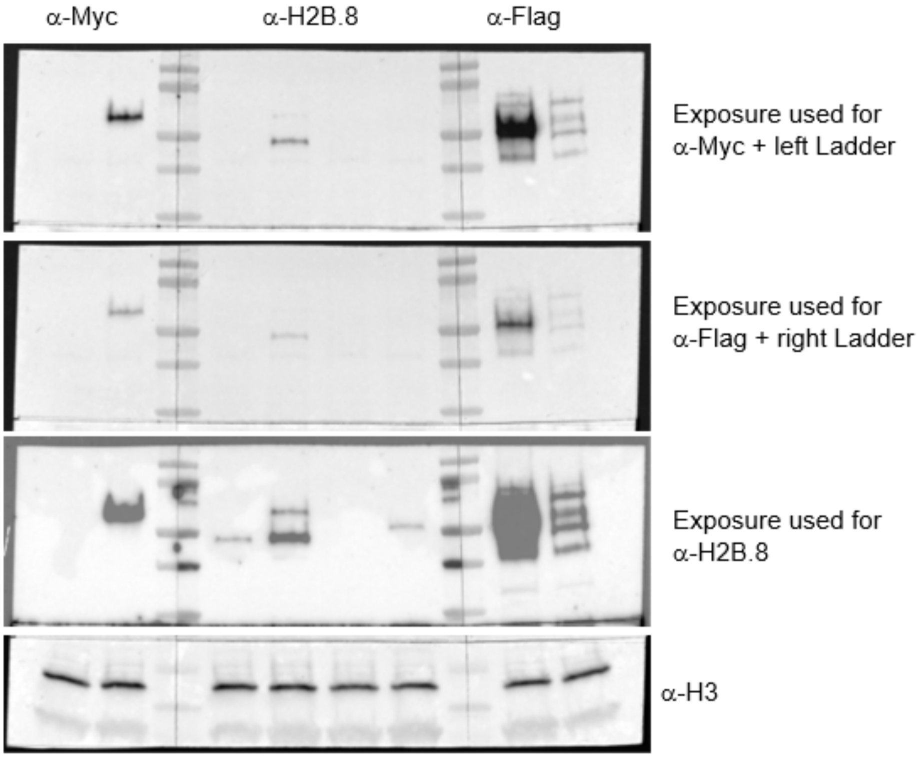

Figure 2b

Immunoprecipitation of Myc-H2B.8 containing nucleosomes

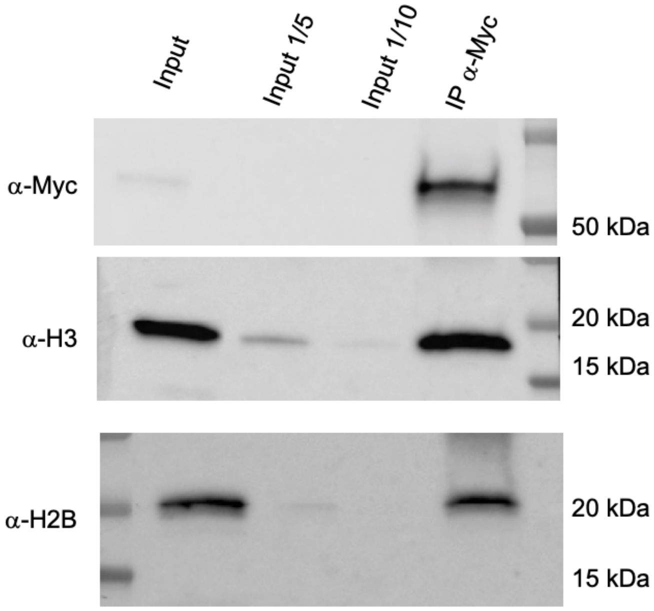

Figure 2b

Negative control, immunoprecipitation using the anti-Myc antibody in WT plants

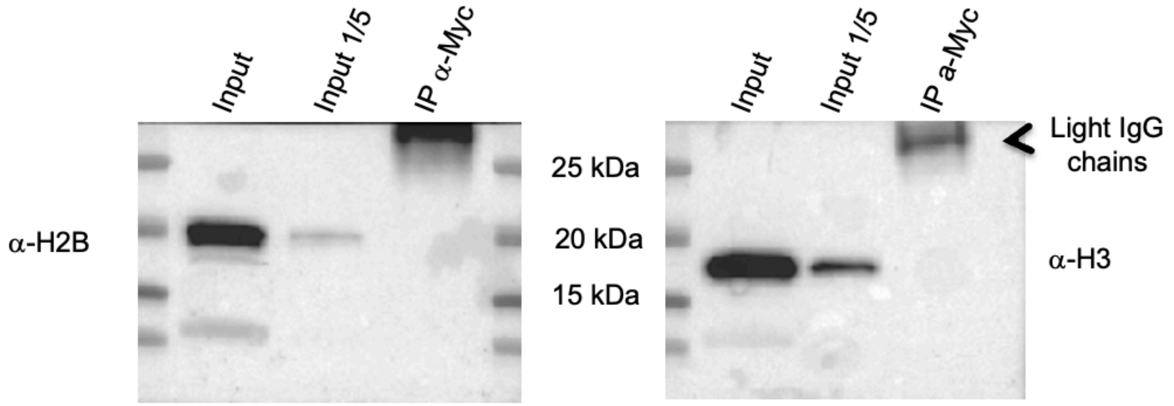

Figure 4d

Replicate 1

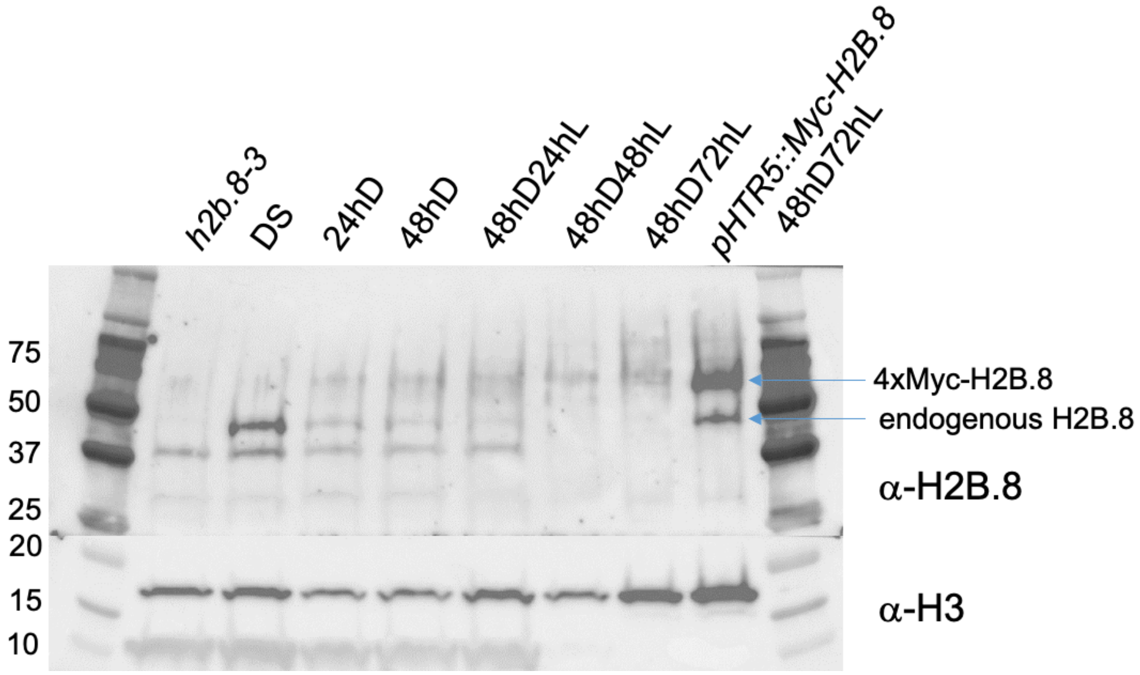

Replicate 2

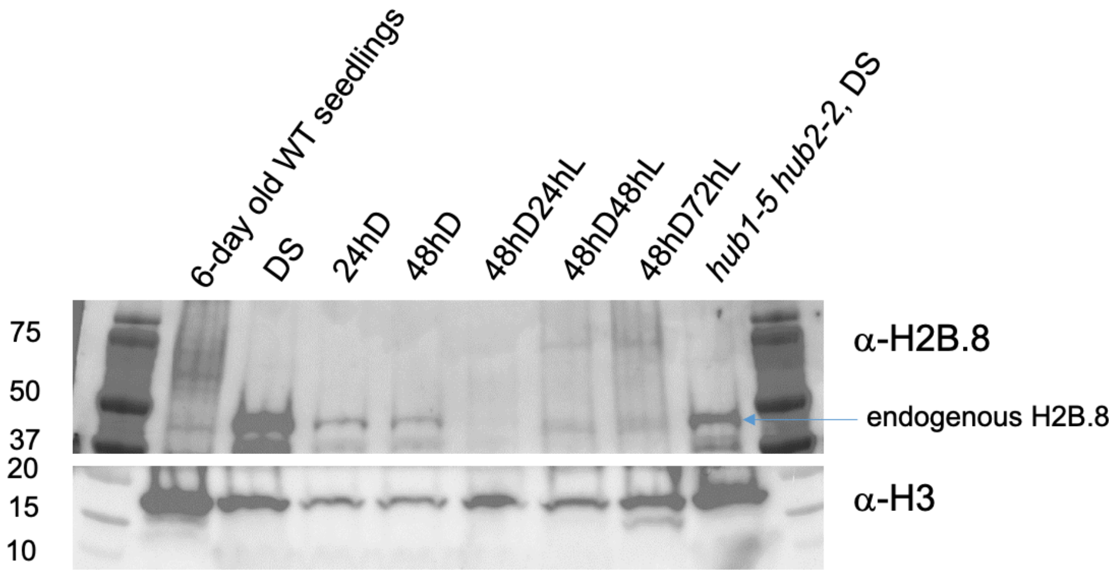

Replicate 3

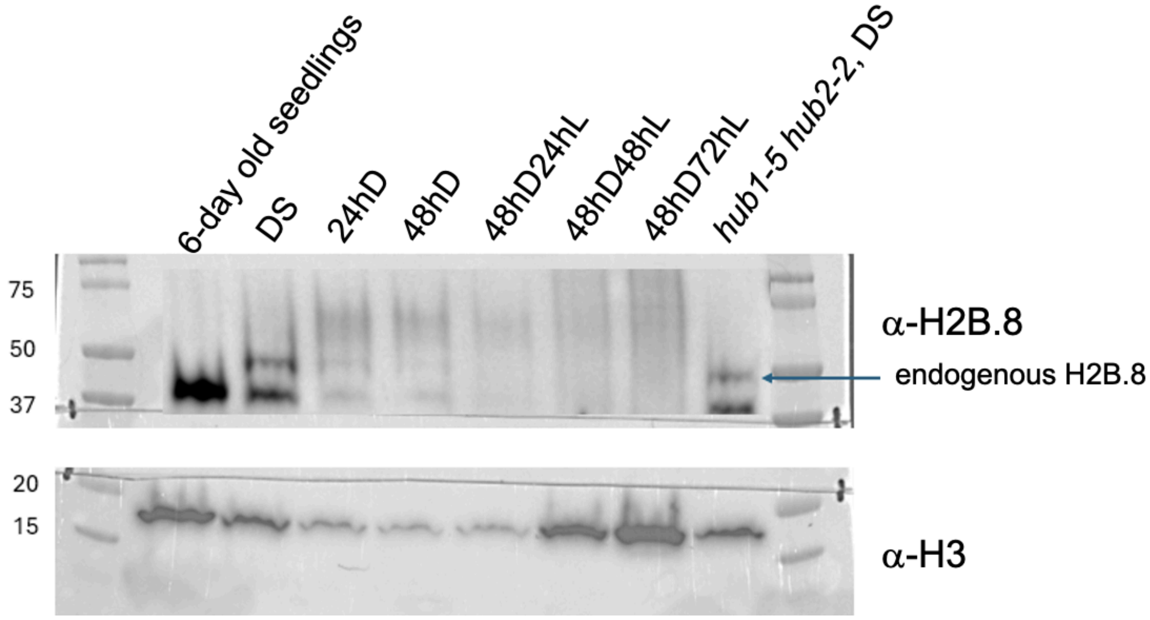

Replicate 4 (used in Figure 4d)

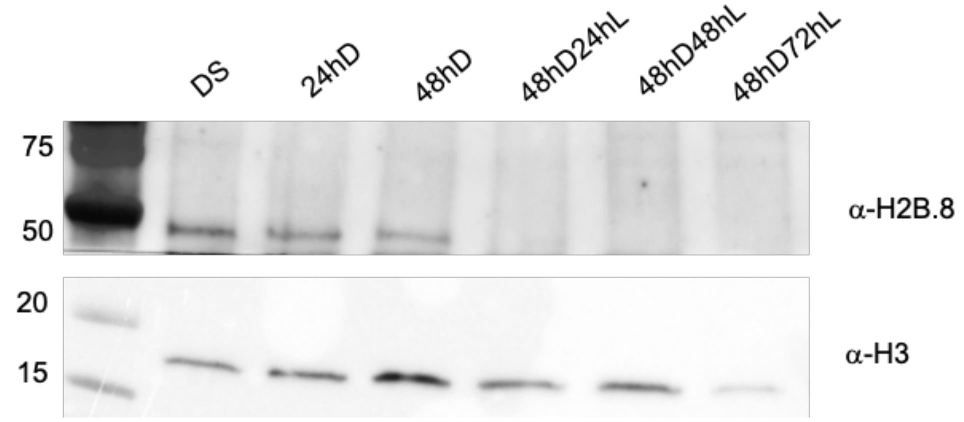

Figure 5a

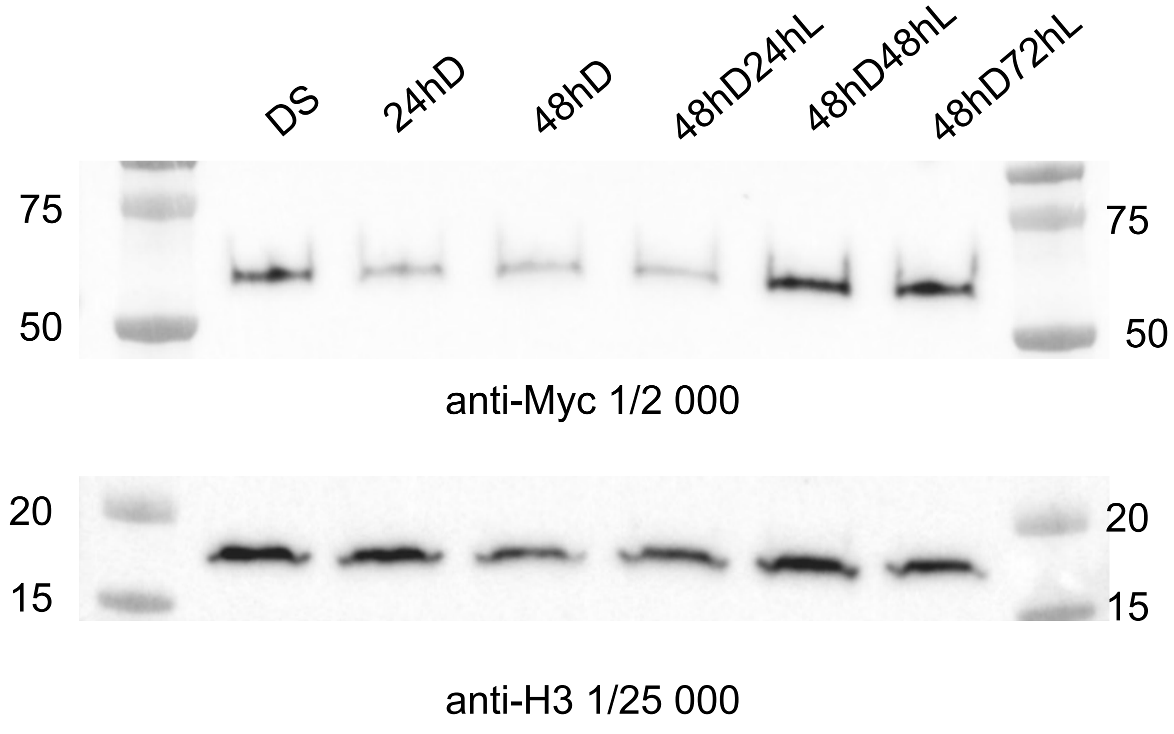

Independent biological replicate of experiment from Figure 5a – not used in the Figure.

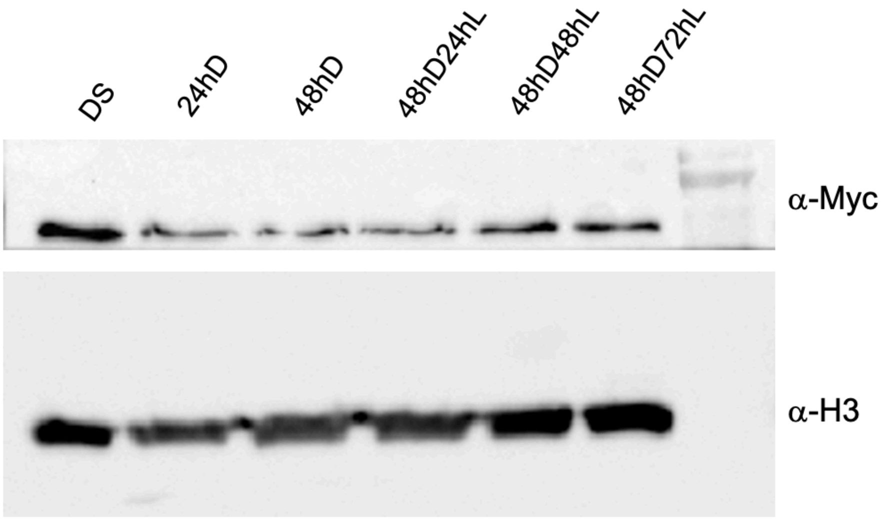

Figure 5b

Dry seeds in WT and *hira-1* mutants, 3 independent seed lots

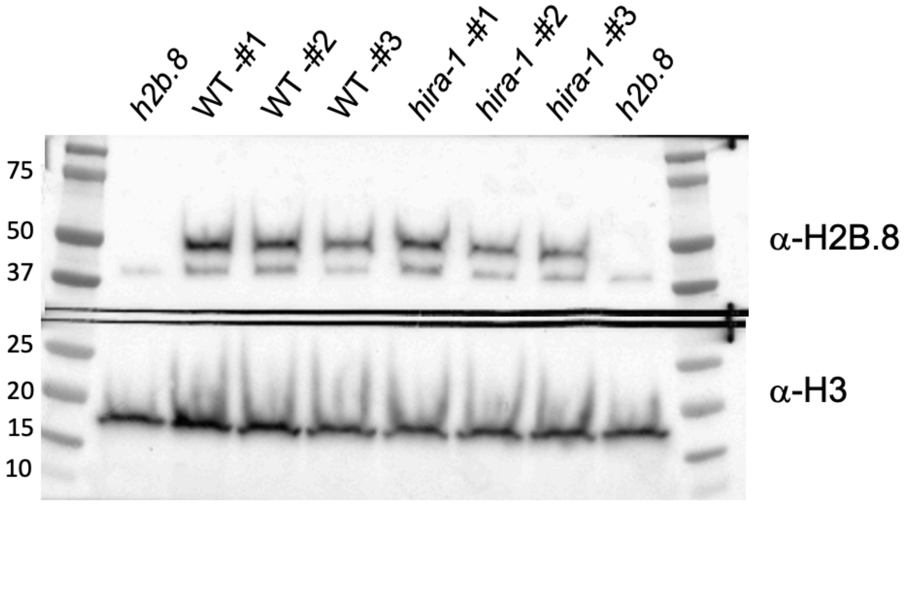

Dry seeds and 48hD (not used) in WT, *hira-1* and *ssrp1* mutants

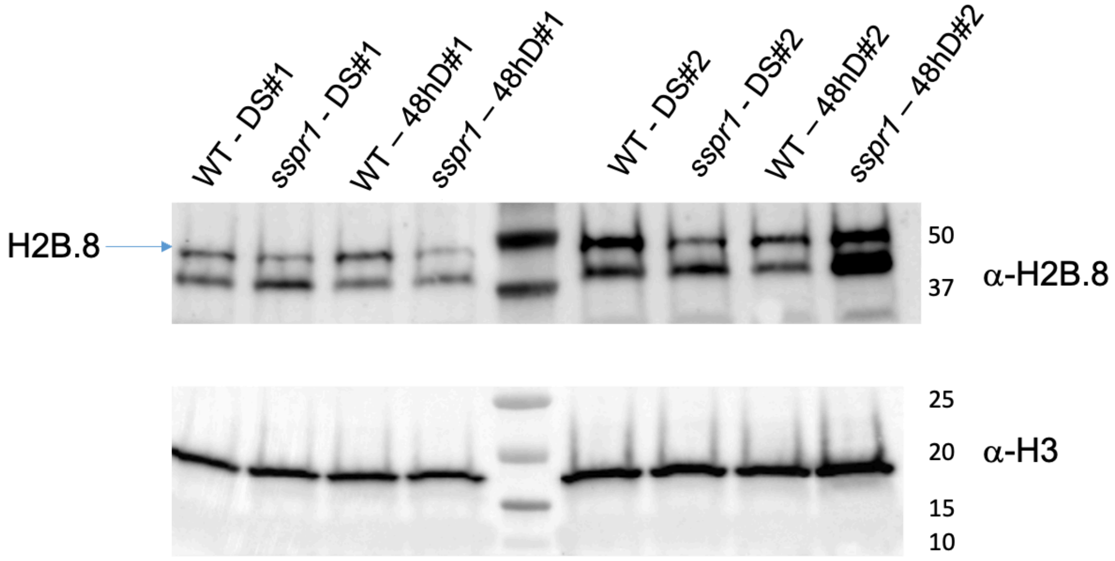

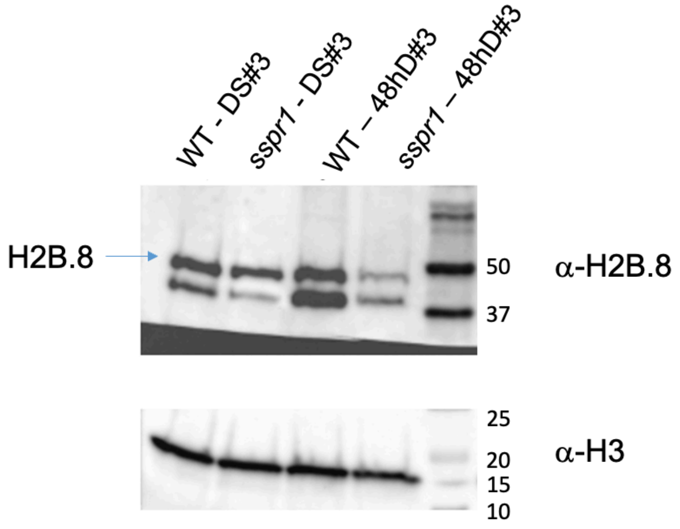

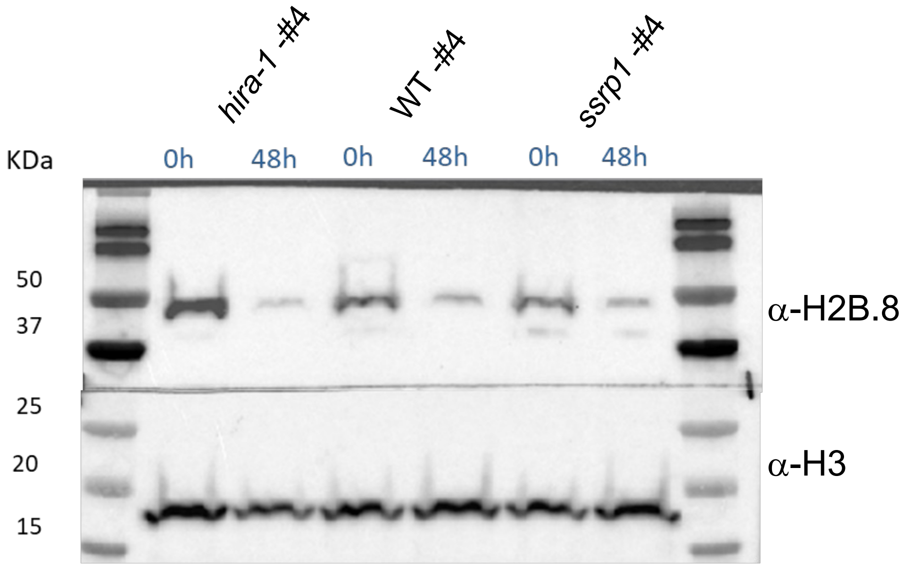
hira-1 -#4
WT -#4
ssrp1 -#4
KDa
0h
48h
0h
48h
0h
48h
50
37
25
20
15
α-H2B.8
α-H3

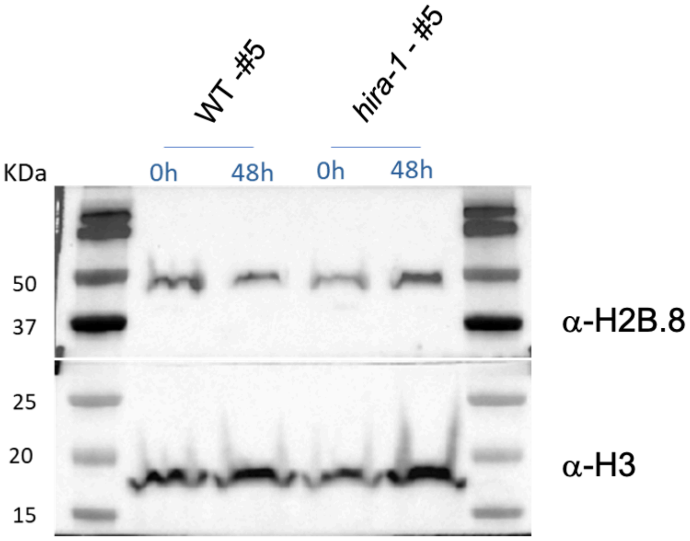
WT -#5
hira-1 - #5
KDa
0h
48h
0h
48h
50
37
25
20
15
α-H2B.8
α-H3

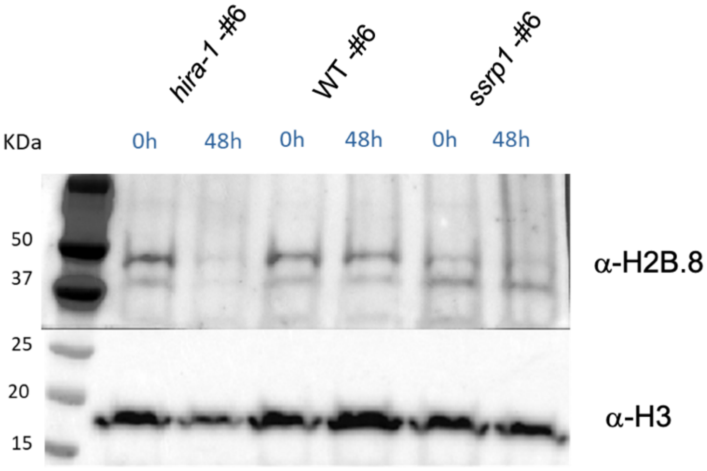
hira-1 -#6
WT -#6
ssrp1 -#6
KDa
0h
48h
0h
48h
0h
48h
50
37
25
20
15
α-H2B.8
α-H3

Figure 5c

Control Western Blot to confirm the correct position of the Flag-H3.3 band,

* Indicates nonspecific band revealed by the anti-Flag antibody in seeds

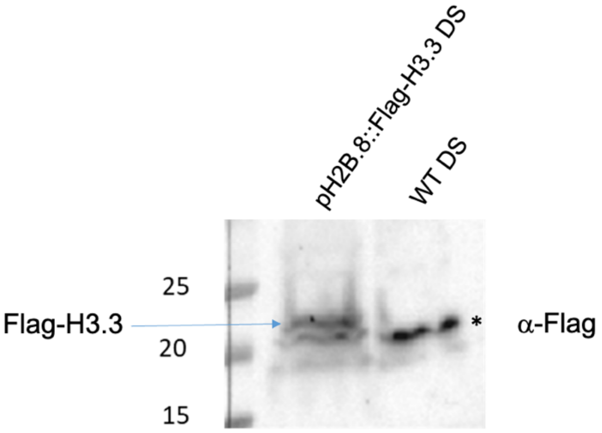

6 different independent transgenic lines expressing Flag-H3.3 under the *H2B.8* promoter, in dry seeds (DS) and at 48h imbibition (48hD)

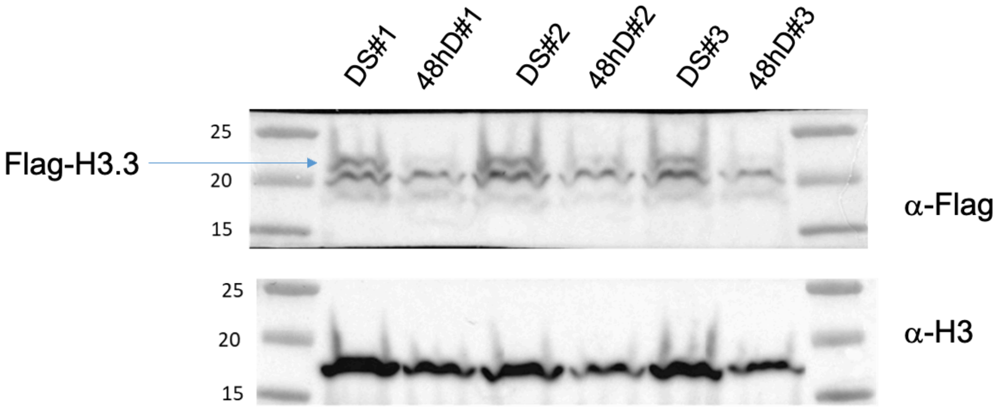

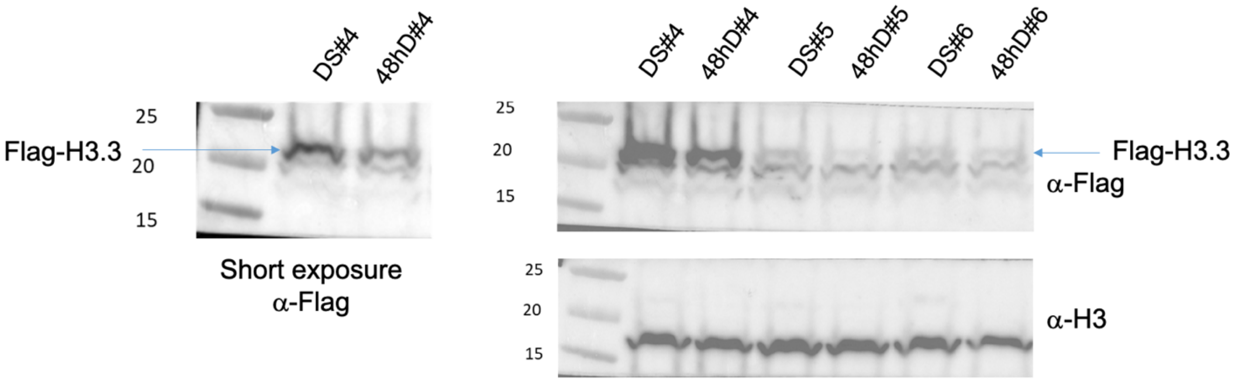

Figure 6d

H2B.8 eviction and degradation under continuous light

3 different WT seeds lots imbibed for 24h or 48h under continuous light

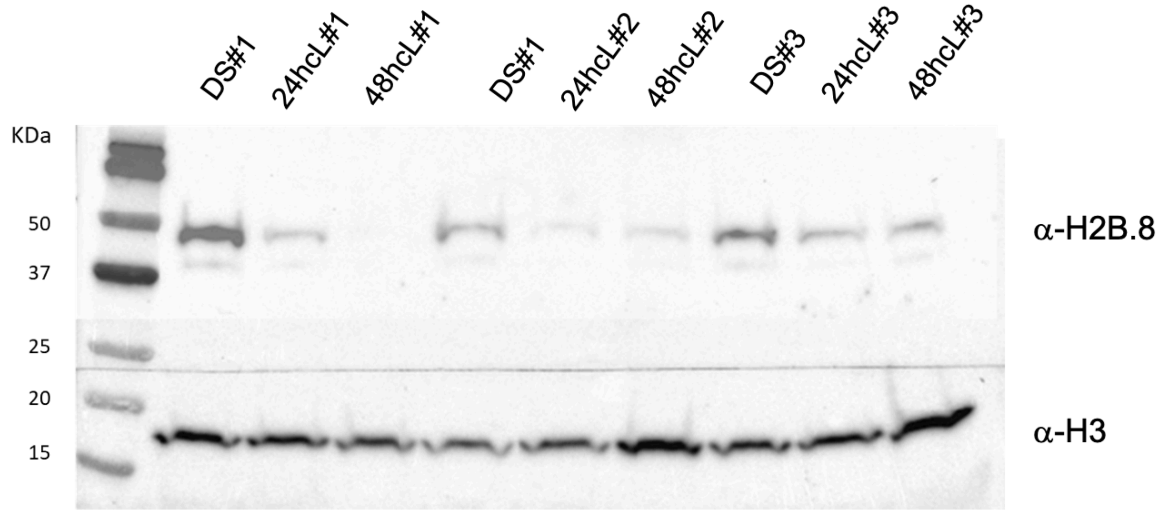

Supplementary Figure 3b

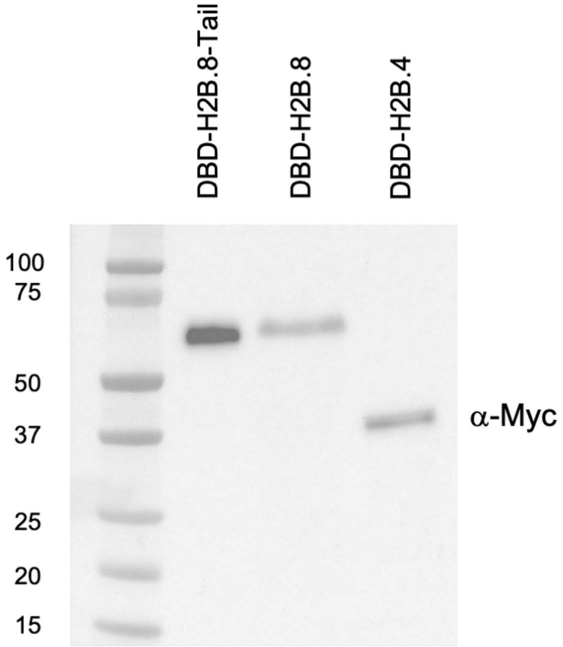

Supplementary Figure 3c

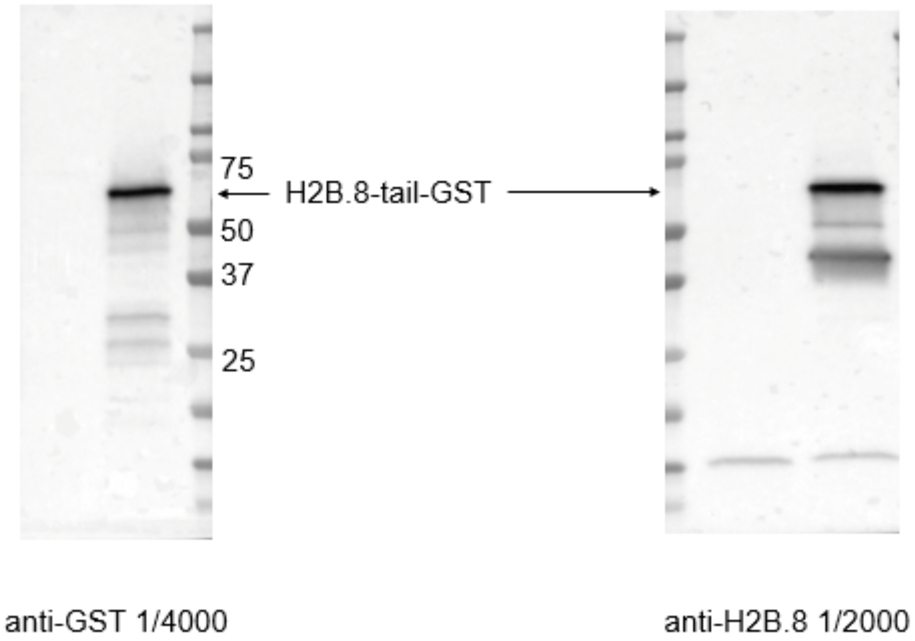

Supplementary Figure 3f

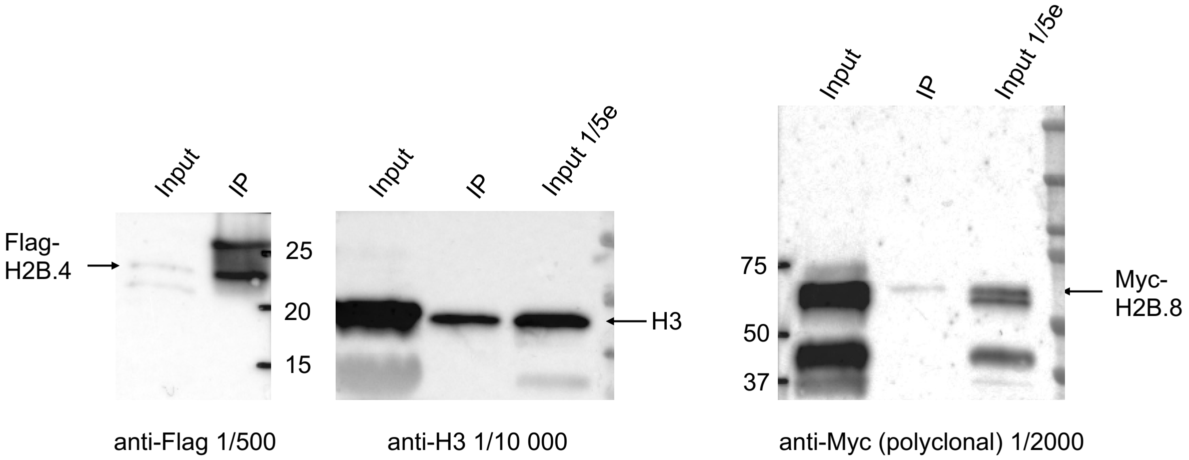

Supplementary Figure 3g

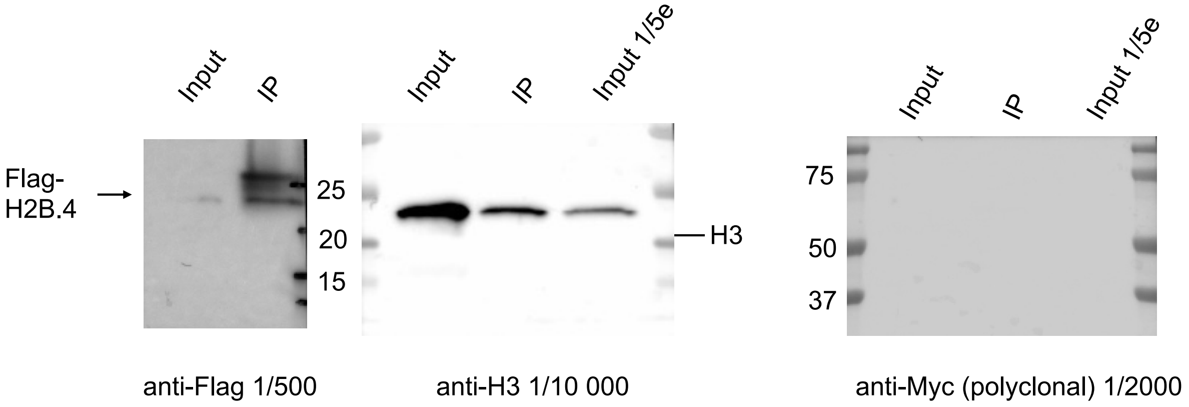

Supplementary Figure 11b

Seeds from transgenic plants expressing Myc-H2B.8 under its endogenous promoter

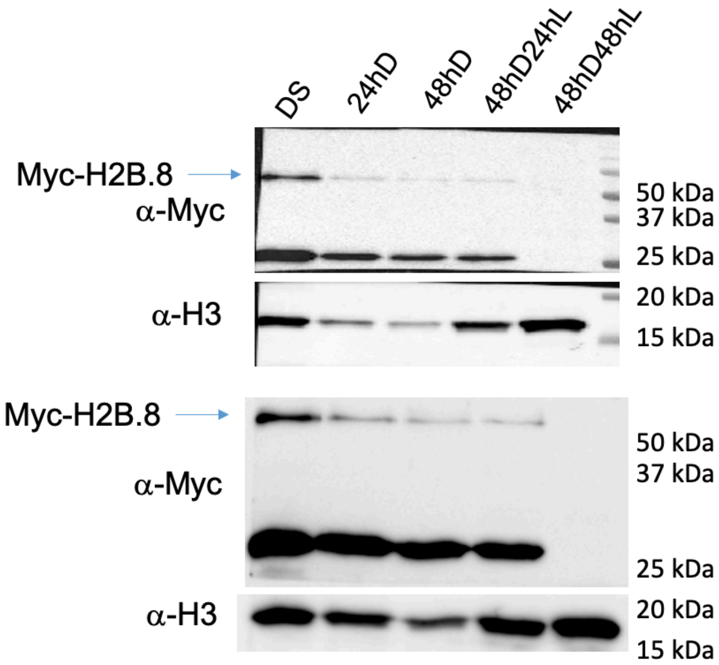

Original images of TTC treatment in 3 replicates (Supplementary Figure 14h)

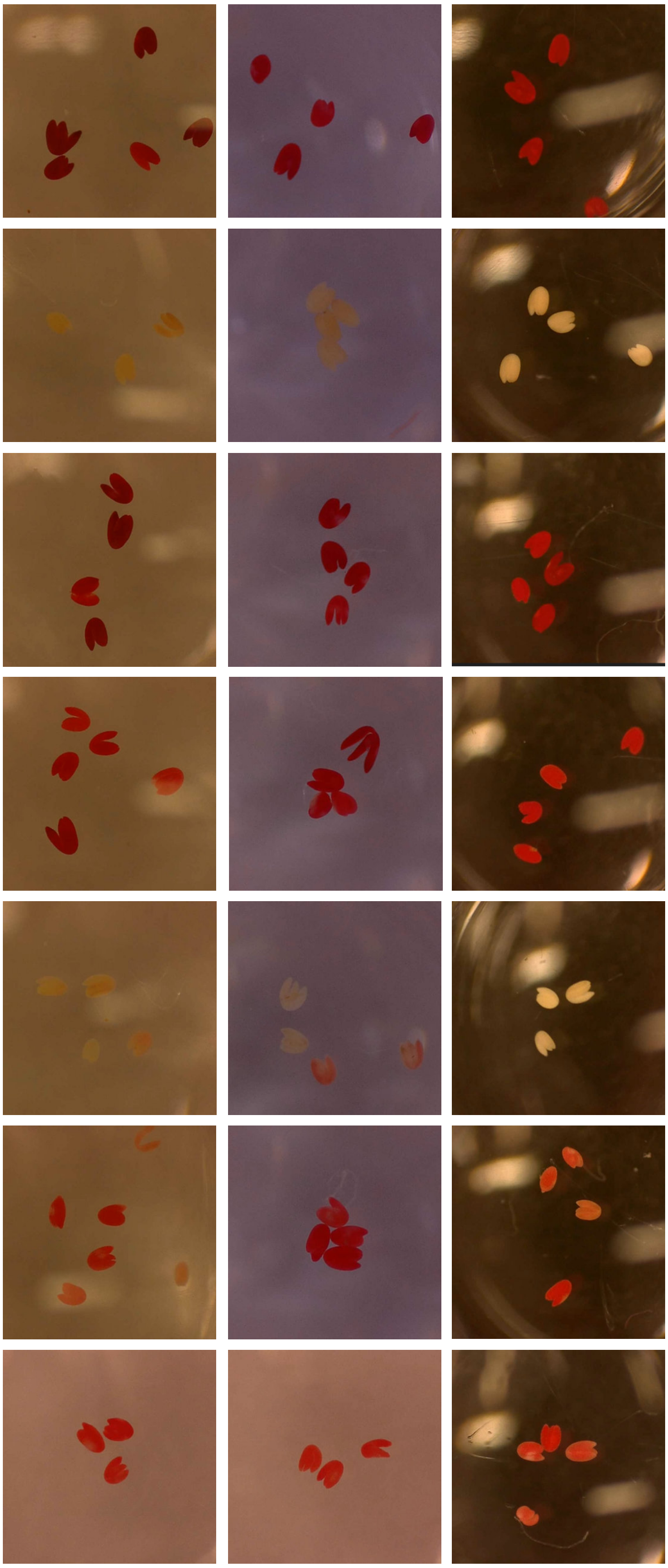

Water

Control - dead embryos

MG132

MG132 + Bortezomib

Cycloheximide

α-amanitin, 500 μM

α-amanitin, 250 μM
